# Supplementary material for: Tunable and functional deep eutectic solvents for lignocellulose valorization
Source: Nat Commun. 2021 Sep 14;12:5424. doi: 10.1038/s41467-021-25117-1 (PMC8440657; doi:10.1038/s41467-021-25117-1)
Supplement: Supplementary file 1 — Supplementary Information [file 41467_2021_25117_MOESM1_ESM.pdf]

Supplementary Information

Tunable and functional deep eutectic solvents for lignocellulose valorization

Yongzhuang Liu[1,2], Noemi Deak[3], Zhiwen Wang[4], Haipeng Yu[1], Lisanne Hameleers[5], Edita Jurak[5], Peter J. Deuss[4] & Katalin Barta[2,3*]

Correspondence should be addressed to: Katalin Barta; k.barta@rug.nl and katalin.barta@uni-graz.at

Table of Content

Supplementary Methods 4

1.1 Materials and Methods 4

1.2 Composition analysis of the cellulose residue and DP-DES or CS-DES fractionated lignin 7

1.3 Milled wood lignin isolation procedure 7

1.4 Enzymatic hydrolysis of the DES fractionated cellulose residues 7

Supplementary Notes 8

Supplementary Note 1 Related ternary DES systems in the literature 8

Supplementary Note 2: Cost of DES calculation 9

2.1 Discussion about the price and availability of the used DES 9

2.2 Synthetic routes for the preparation of the described DES from petrochemicals versus renewables 10

Supplementary Note 3: Model compound studies 10

3.1 Procedure for lignin model compound synthesis and characterizations 10

3.1.1 Compound 2-(2-methoxyphenoxy)-1-(4-methoxyphenyl)ethanone: 10

3.1.2 Compound 2-(2-methoxyphenoxy)-1-(4-methoxyphenyl)ethanol (1a) 12

3.1.3 Compound Erythro/Threo-2-(2-methoxyphenoxy)-1-(4-methoxyphenyl)propane-1,3-diol (1b) 14

3.1.4 Compound 2-(2-(2-methoxyphenoxy)-1-(4-methoxyphenyl)ethoxy)ethanol (4aa) 17

3.1.5 Compound 3-(2-hydroxyethoxy)-2-(2-methoxyphenoxy)-3-(4-methoxyphenyl)propanol (4ba) 19

3.1.6.Compound3-(1-(4-hydroxy-3-methoxyphenyl)-2-(2-methoxyphenoxy)ethoxy)propane-1,2-diol (4ab) and 2-(1-(4-hydroxy-3-methoxyphenyl)-2-(2-methoxyphenoxy)ethoxy)propane-1,3-diol(4ab′) 21

3.1.7Compound4-(3-hydroxy-1-(2-hydroxyethoxy)-2-(2-methoxyphenoxy)propyl)-2-methoxyphenol (4da) 25

Supplementary Note 4: In-depth studies regarding the nature of DES 27

4.1 Elucidating the nature of interactions between the ternary DES compositions 27

4.1.1 Analysis of three different DES compositions by diverse NMR spectroscopy techniques 27

4.1.2 IR measurements of DES 29

4.2 Computational studies related to different DES compositions 29

4.2.1 DFT calculation of DESs 29

4.2.2 Molecular dynamic simulation of DESs 30

Supplementary Note 5: DES for lignocellulose fractionation studies 30

5.1 Thermal behaviour and characterization of the obtained lignins 30

5.1.1 Thermal degradability 30

5.2.2 Pyrolysis GC-MS 31

5.2 Control experiments related to SEM imaging of condensed lignin. 31

5.3 In depth sugar analysis 31

5.3.1 Analysis of the behavior of xylan and MCC in DES 31

5.3.2 In–depth composition analysis of the obtained CR 32

Supplementary Note 6: DES recycling 33

6.1 DES recycling 33

6.1.1 Quantification of losses of ChCl and EG during recycling by ^{1}H NMR spectroscopy 33
6.1.2 Quantification of losses of Oxalic acid (OA) during recycling 33
6.1.3 Investigation of purity of the recycled DES and its effectiveness for further fractionation 33
Supplementary Note 7: Characterization of CRs by XRD and BET 34
Supplementary Figures 35
Supplementary Tables 61
Supplementary References 75

Supplementary Methods

1.1 Materials and Methods

Materials: All the chemicals were used as received from TCI or Sigma Aldrich without any purification. Choline chloride (ChCl), oxalic acid dihydrate (OA) and ethylene glycol (EG) used for deep eutectic solvents (DESs) preparation and *n*-octadecane and 3, 5-dimethylphenol were used as an internal standard were purchased from Sigma-Aldrich. Lignin model compounds were prepared as previously reported (Supplementary Section 4.1). Birch lignocellulose was kindly provided by A.Bakker, Harkstede, and used after soxhlet extraction. Tetrahydrofuran (THF), dichloromethane (DCM), ethanol (EtOH), methanol (MeOH) and distilled water were used for extraction workup.

Column chromatography was performed using Merck silica gel type 9385 230-400 mesh and typically ethyl acetate and pentane or methanol and dichloromethane as an eluent.

TLC: Merck silica gel 60, 0.25 mm. The components were visualized by UV or $KMnO_4$ staining.

Gas Chromatography was used for product identification as well as determination of conversion and selectivity values. Product identification was performed by GC-MS (Shimadzu QP2010 Ultra) with an HP-1MS column, and helium as a carrier gas. GC-MS method for hydrogenolysis analysis: the temperature program started at 40 °C for 10 min, heated by 10 °C/min to 90 °C and held for 5 min, then heated by 15 °C/min to 260 °C and held for 5 min, after heated by 25 °C/min to 290 °C and held for 0 min. GC-MS method for acidolysis analysis and model compound reaction analysis: the temperature program started at 45 °C for 0 min, heated by 12 °C/min to 300 °C and held for 10 min.

Conversions and product selectivities were determined by GC-FID (Shimadzu GC-2014) with an HP-5MS column using nitrogen as a carrier gas. GC-FID analysis method for hydrogenolysis: the temperature program started at 40 °C for 10 min, heated by 10 °C/min to 90 °C and held for 5 min, then heated by 15 °C/min to 260 °C and held for 5 min, after heated by 25 °C/min to 290 °C and held for 0 min. For GC-FID analysis method for acidolysis and model compound reaction analysis: the temperature program started at 40 °C for 5 min, heated by 10 °C/min to 140 °C and held for 10 min, then heated by 10 °C/min to 290 °C and held for 0 min.

The lignins ChCl/OAL and DPL10 were further characterized by Agilent PY-3030D/7890B-5977A pyrolysis GC-MS (Agilent Technologies Co. Ltd, USA). The pyrolysis was performed at 500°C for 30s, the oven temperature was programmed from 40°C to 220°C at 5°C/min, Helium (99.999%, 1mL/min) was used as carrier gas, all the compounds were identified by comparing the mass spectra with those of the Wiley and NIST libraries.

Mass spectrometry: Mass spectra were recorded on an AEI-MS-902 mass spectrometer (EI^+) or a LTQ Orbitrap XL (ESI^+).

NMR spectroscopy: ^1H and ^{13}C NMR spectra were recorded on a Varian Mercury Plus 400, Agilent MR 400 (400 and 100.59 MHz, respectively) and Bruker Advanced NEO 600 (600 and 150.92 MHz, respectively) using $CDCl_3$ or acetone-d_6 as a solvent. ^1H and ^{13}C NMR spectra were recorded at room temperature. Chemical shift values are reported in ppm with the solvent resonance as the internal standard ($CDCl_3$: 7.26 for ^1H, 77.2 for ^{13}C; acetone-d_6: 2.05 for ^1H, 29.84 and 206.26 for ^{13}C). Data are reported as follows: chemical shifts, multiplicity (s = singlet, d = doublet, t = triplet, q = quartet, br. = broad, m = multiplet), coupling constants (Hz), and integration. The NMR experiments realized for elucidating the DES structures were recorded on a Bruker Avance III 500 MHz and a Bruker Avance III 700 MHz spectrometer.

Thermogravimetry-Infrared Spectroscopy (TG-FTIR): The pyrolysis behaviour of the obtained lignins was characterized by TG-FTIR (PerkinElmer, STA 6000-SQ8, USA). The pyrolysis process was carried out under N_2 (over 99.99%) atmosphere. The temperature program started from room temperature to 800 °C with a heating rate of 20 °C/min.

High performance anion exchange chromatography (HPAEC): The hemicellulose retention and composition residues after different DES treatments were analyzed with HPAEC-PAD on a Dionex Ultimate 6000 system (Thermo Scientific, Sunnyvale, CA, USA) equipped with a CarboPac PA-1 column (2 mm x 250 mm ID) in combination with a CarboPac PA-1 guard column (2 mm x 50 mm ID) and PAD detection. System was controlled by the Chromeleon 7.2.9 software (Thermo Scientific, Sunnyvale, CA, USA).

High performance liquid chromatography (HPLC): The yield of glucose and xylose after hydrolysis were determined by HPLC (Agilent 1260 series, Agilent Technologies, USA) with an autoinjector and RID detector. An HPX-87H (BIO-RAD, USA) with a 4 mM H_2SO_4 eluent at 0.6 ml/min was used.

Field emission scanning electron microscope (FE-SEM): The morphology of the fractionated CRs was observed by FE-SEM (FEI, Apreo S HiVac) at 10-15 kV. All the samples were dried at 60°C overnight and coated with platinum using a vacuum sputter coater before observations.

Molecular simulations were used to further investigate the hydrogen bond interaction in the DES systems. Gromacs 2016.1 software package was used for the simulations with a temperature of 373K using a time step of 2fs. The V-rescale thermostat[1] and Parrinello–Rahman barostat[2] were used for controlling the temperature and pressure. Hydrogen containing atoms were constrained using LINCS algorithm.[3] Periodic boundary conditions were used, the Particle Mesh Ewald (PME) algorithm was used for long-range electrostatic interactions, the switch function for the van der Waals and electrostatic interactions was 1.0 nm. Gromos54A7 field was used for choline chloride (ChCl), oxalic acid (OA) and ethylene glycol (EG) from ATB library and SPC water model was employed.[4] Typically, two binary DES systems (100 ChCl and 200 EG or 100 ChCl and 20 OA molecules in the box, respectively) and a ternary DES system (100 ChCl, 200 EG and 20 OA molecules in the box, representing DP-DES10) were created by Packmol package and subsequently simulated, respectively. After energy minimization, the simulations were run for 500 ns under the NPT system 373 K and ambient pressure, the last 100ns simulation was extracted for analysis. All the data were analyzed and visualized with the help of Gromacs' tools and Visual Molecular Dynamics (VMD) package.

The density functional theory (DFT) calculations were used to investigate the interaction between HBA and HBD in DES. All DFT calculations were performed at the Gaussian 16 software package. The geometries of DESs were fully optimized using B3LYP/6-311+G** basis set.[5]

1.2 Composition analysis of the cellulose residue and DP-DES or CS-DES fractionated lignin

The composition of the solid samples was determined according to the NREL procedure.[6] The monosaccharide was determined by HPLC (1260 Agilent Technologies, USA) with a refractive index detector (RID). An aminex column HPX-87H (Bio- Rad, USA) was used to analyze the monosaccharides at 50 °C with 5 mM sulfuric acid at a flow rate of 0.6 mL/min. In these condition, xylose, mannose and galactose was eluted at the same retention time which were integrated at a single peak. The composition of birch is: 39.4% cellulose, 27.9% hemicellulose, 19.3% acid insoluble lignin (AIL) and 1.1% acid soluble lignin (ASL). Elemental analysis (C, H and N) of protected lignin was performed using an Elementar VarioMICRO Cube, 3-4 mg of protected lignin was used to determine the C, H and N content. Typical DP-DES extracted lignin DPL10 contains: C: 60.12 %, H: 6.77%, N: 0.25%, the result was comparable with typical organosolv lignin.[7]

1.3 Milled wood lignin isolation procedure

The ball wood lignin was extracted by following from published steps.[8] 20 g wood powders were suspended in 400 mL dioxane/water (v/v, 96:4) and stirred for 24 h under dark, and the residue was collected by centrifugation and extracted by fresh solvent for another 24 h. The liquid was combined and condensed to approximately 30 mL, and further precipitated in 3x the volume of 96% ethanol, the ethanol phase was condensed to around 30 mL (this step was repeated) and precipitated in 10 volume times acid water (pH=2), the isolated lignin was further washed with acidic water and freeze-dried.

1.4 Enzymatic hydrolysis of the DES fractionated cellulose residues

Enzymatic hydrolysis of the cellulose residues was performed as follows: 7.5 mL of 4% (w/v) solid samples (0.3 g) in a buffer (sodium acetate, 50 mmol/L, pH 5.5, 2.88 mg tetracycline chloride) was added into a 50 mL tube with cap, and the tubes were kept at 50 °C in a VWR incubating orbital shaker (Model 3500 L) at 250 rpm for 72 h. Enzymatic hydrolysis was conducted with CTec2 (Novozymes, Denmark, 0.1 mL/g substrates) and 0.3 mL of the hydrolysate was sampled periodically to determine the released sugar amount. After the inactivation of the enzyme (5 min 100 °C), the glucose and xylose were determined by HPLC (Agilent 1260 series, Agilent Technologies, USA) with an autoinjector and RID detector. An HPX-87H (BIO-RAD, USA) with a 4 mM H_2SO_4 eluent at 0.6 ml/min was used.

Supplementary Notes

Supplementary Note 1: Related ternary DES systems in the literature[9–18]

- **Ref 9:** Tang, W., et. al. Evaluating ternary deep eutectic solvents as novel media for extraction of flavonoids from Ginkgo biloba. *Separation Science and Technology*, **2017**, 52(1), 91-99.

This paper deals with the extraction of flavonoids using aqueous ternary DES (ChCl: OA: EG).

- **Ref 10:** Jiang, Z. et. al. Green and efficient extraction of different types of bioactivealkaloids using deep eutectic solvents. *Microchemical Journal*, **2019**, 145, 345–353.

This paper screened a total of 75 types of binary or ternary DESs (including one ternary combination with ChCl:OA: EG) for morphinane alkaloids extraction.

- **Ref 11:** Chen Z et. al, Ternary deep eutectic solvents for effective biomass deconstruction at high solids and low enzyme loadings. *Bioresource Technology*, **2019**, 279, 281-286.

Here, the ternary DES (guanidine hydrochloride/EG/p-TSA and also ChCl/EG/p-TSA) was used for lignocellulose fractionation showing xylan and lignin removal from switchgrass and cellulose retention. The 2D NMR of the lignins showed significant change in lignin interunit linkages.

- **Ref 12:** Ji Q., et. al. Efficient removal of lignin from vegetable wastes by ultrasonic and microwave-assisted treatment with ternary deep eutectic solvent. *Industrial Crops and Products*, **2020**, 149, 112357.

In this paper, the ternary DES (ChCl-Glycerol-$AlCl_3 \cdot 6H_2O$) was used for removing lignin from garlic skin and green onion root. No lignin characterization or tunability of the system was shown.

Other recent ternary or relevant DES literature:

- **Ref 13:** Xue B. et al. Efficient dissolution of lignin in novel ternary deep eutectic solvents and its application in polyurethane. *International Journal of Biological Macromolecules*, **2020**, 164, 480–488.

In this paper, Ternary DES (ChCl:Gly:PEG-400) was used to dissolve lignin for polyurethanes application, the dissolution of lignin in DES didn’t change the lignin structure.

- **Ref 14:** Chen Z. et al.Insights into Structural Changes of Lignin Toward Tailored Properties during Deep Eutectic Solvent Pretreatment. *ACS Sustainable Chem. Eng*. **2020**, 8, 9783−9793.

In this paper, they used H_2SO_4 acidified ChCl/EG DES for switchgrass fractionation to obtained lignin, the lignin characterized by 2D NMR showed well preserved β−O−4 linkages (highest 40.3%).

- **Ref 15:** Jiang J. et al. High Production Yield and More Thermally Stable Lignin-Containing Cellulose Nanocrystals Isolated Using a Ternary Acidic Deep Eutectic Solvent. *ACS Sustainable Chem. Eng*. **2020**, 8, 7182−7191.

This paper described the use of ternary DES (ChCl: OA: p-TSA) for lignin containing cellulose nanocrystals production.

- **Ref 16:** Saputra R. et al. Synthesis and thermophysical properties of ethylammonium chloride-glycerol-$ZnCl_2$ ternary deep eutectic solvent. *Journal of Molecular Liquids*, **2020**, 310, 113232.

This paper mainly focus on synthesis and thermophysical properties of ternary DES (ethylammonium chloride/glycerol/$ZnCl_2$).

- **Ref 17:** Farajzadeh M. A. et al. Preparation of a new three-component deep eutectic solvent and its use as an extraction solvent in dispersive liquid-liquid microextraction of pesticides in green tea and herbal distillates. *J Sci Food Agric***2020**; 100, 1904–1912.

This paper described the use of ternary DES (dichloroacetic acid, L-menthol, and n-butanol) for extraction of pesticide residues from green tea.

- **Ref 18:** Fu N. et al. Ternary choline chloride/caffeic acid/ethylene glycol deep eutectic solvent as both a monomer and template in a molecularly imprinted polymer. *Journal of Separation Science*,**2017**, 40, 2286-2291.

This paper described the use of ternary choline chloride/caffeic acid/ethylene glycol deep eutectic solvent as both a monomer and template in synthesizing a molecularly imprinted polymer.

Supplementary Note 2: Cost of DES calculation

2.1 Discussion about the price and availability of the used DES

In the paper of Słupek E. et. al. in Energies, 2020, 13, 3379 a cost calculation for ChCl/OA and ChCl/urea DESs was provided from Alibaba.[19] In the paper of Singh in Green Chem, 2017, 19 (13), 3152-3163 a cost calculation of choline based ionic liquids, was also

calculated based on prizes from Alibaba.[20] In the publication of Wan and coworkers in Biofuels, Bioprod. Bioref. 2020, 14, 326–343, the price of ChCl was indicated as 1200$ ton^{-1} and that of EG as 838$ ton^{-1} based on Intratec Solutions, LLC[21], consistent with our values added.

The cost of DP or CS-DES was calculated based on the price of choline chloride (ChCl), oxalic acid dihydrate (OA) and ethylene glycol (EG). The calculation was as follows:

Cost of DES= Price of ChCl× Weight percentage of ChCl + Price of OA × Weight percentage of OA + Price of EG × Weight percentage of EG

Regarding the cost of the individual components, this depends, and will in the future depend on many factors such as reactants price (and their availability from fossil *versus* renewable resources), synthetic method, scale, and market demands.[22–28]

2.2 Synthetic routes for the preparation of the described DES from petrochemicals versus renewables

On Supplementary Figures 1 and 2 possible synthetic routes for the preparation of ChCl and EG are displayed.

Supplementary Note 3: Model compound studies

3.1 Procedure for lignin model compound synthesis and characterizations

3.1.1 Compound 2-(2-methoxyphenoxy)-1-(4-methoxyphenyl)ethanone:

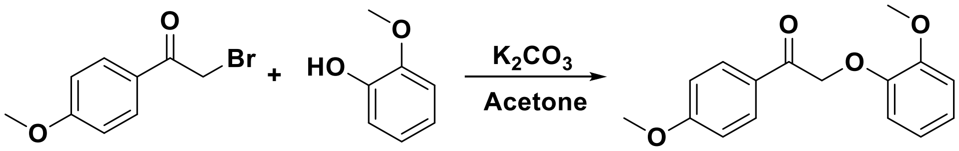

2-(2-methoxyphenoxy)-1-(4-methoxyphenyl)ethanone was prepared according to a literature procedure.[30] A 500 mL round bottom flask equipped with a reflux condenser was charged with potassium carbonate (6.15 g, 44.5 mmol) and guaiacol (4.6 g, 37 mmol) in acetone (100 mL) and stirred at room temperature. 2-bromo-1-(4-methoxyphenyl)ethanone (6.77 g, 30 mmol) in acetone (100 mL) was added dropwise to the solution over 30 min at room temperature. The resulting suspension was stirred and heated to reflux for 4 h, after which it was filtered through celite and concentrated *in vacuo*. The resulting solid was purified by recrystallization from ethanol to give 2-(2-methoxyphenoxy)-1-(4-methoxyphenyl)ethanone

as a light yellow solid in 80.4% yield.

^{1}H NMR ($CDCl_3$, 600 MHz): δ 7.94 (d, J = 9.0 Hz, 2H), 6.89–6.87 (m, 3H), 6.84 (d, J = 8.4 Hz, 1H), 6.77 (d, J = 4.2 Hz, 2H), 5.20 (s, 2H), 3.80 (s, 3H), 3.79 (s, 3H). **^{13}C NMR** ($CDCl_3$, 151 MHz): 193.27, 164.07, 149.90, 147.78, 130.64, 127.87, 122.46, 120.93, 114.91, 114.07, 112.34, 72.16, 56.06, 55.63. **HRMS** (ESI) m/z calculated for $C_{16}H_{16}O_4Na$ ($[M+Na]^+$) 296.09743, found 296.09768. Spectral data are in accordance with those previously reported.[30]

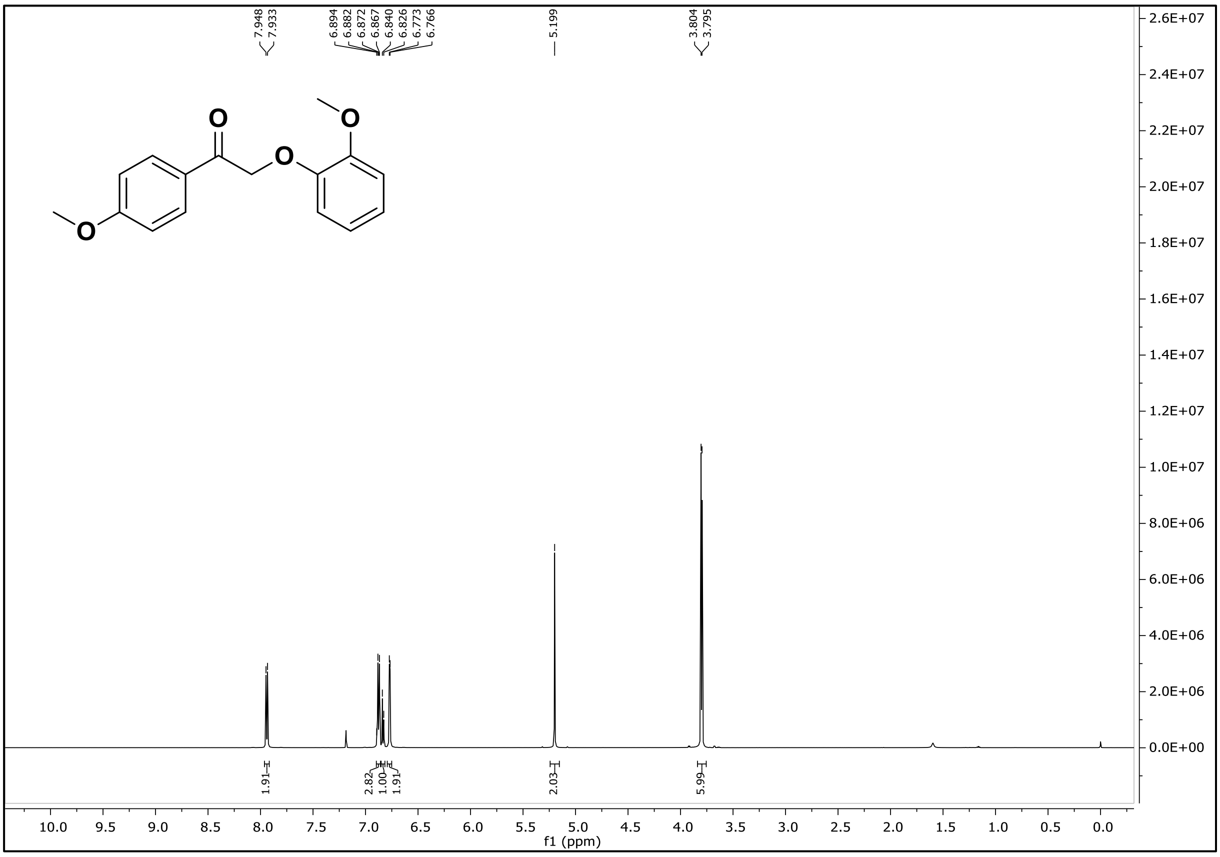

^{1}H NMR spectrum of 2-(2-methoxyphenoxy)-1-(4-methoxyphenyl)ethanone.

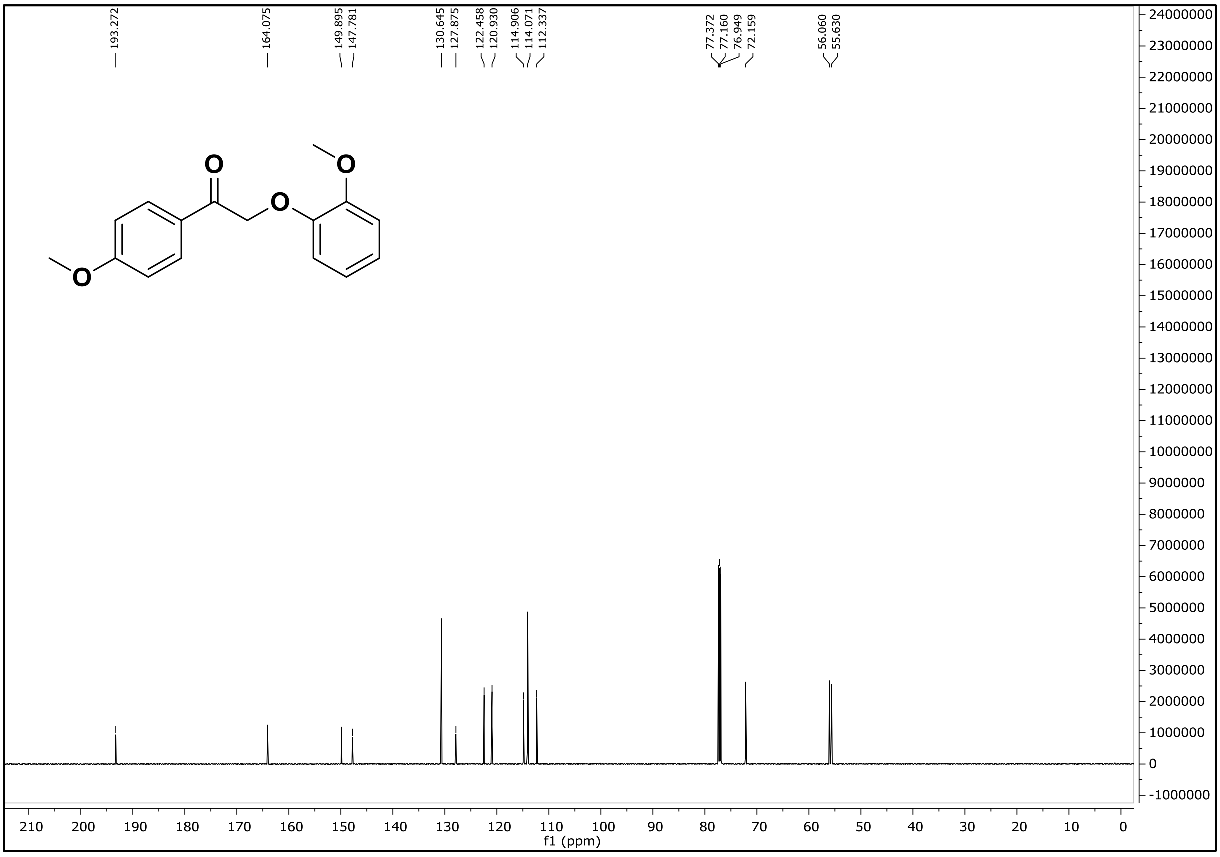

^{13}C NMR spectrum of 2-(2-methoxyphenoxy)-1-(4-methoxyphenyl)ethanone.

3.1.2 Compound 2-(2-methoxyphenoxy)-1-(4-methoxyphenyl)ethanol (1a)

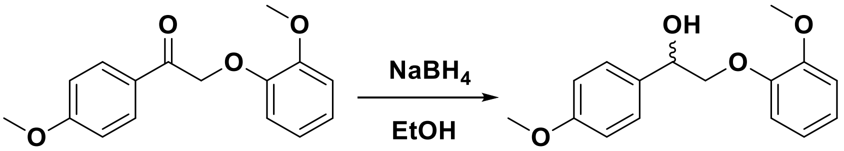

2-(2-methoxyphenoxy)-1-(4-methoxyphenyl)ethanol (**1a**) was prepared according to a literature procedure.[30] A 250 mL round bottom flask was charged with 2-(2-methoxyphenoxy)-1-(4-methoxyphenyl)ethanone (2.7 g, 9.9 mmol) and EtOH (150 mL, 99.5%). Sodium borohydride (0.45 g, 11.7 mmol) was added portion-wise to maintain a gentle evolution of gas over 5 minutes, after which the reaction mixture was stirred for 2 h at room temperature. The reaction mixture was concentrated *in vacuo*. And then quenched with saturated aqueous NH_4Cl (50 mL) and the reaction mixture was diluted with water (50 mL). The aqueous portion was extracted with Et_2O (2 x 100 mL). The combined organic extracts were washed with brine, dried over $MgSO_4$, filtered, and concentrated *in vacuo*. Purification by chromatography on SiO_2 (75:25, Pentane/EtOAc) afforded 2-(2-methoxyphenoxy)-1-(4-methoxyphenyl)ethanol (2.52 g, 9.2 mmol, 93%) as a white solid.

^{1}H NMR($CDCl_3$, 600 MHz): δ 7.36 (d, *J* = 8.4 Hz, 2H) 7.00–6.98 (m, 1H), 6.95-6.89 (m, 5H), 5.06 (dd, *J* = 9.6 Hz, *J* = 3.0 Hz, 1H), 4.15 (dd, *J* = 10.2 Hz, *J* = 3.0 Hz, 1H), 3.97 (t, *J* = 9.6 Hz, 1H), 3.89 (s, 3H), 3.81 (s, 3H). **^{13}C NMR** ($CDCl_3$, 151 MHz): δ 159.54, 150.20, 148.12, 131.77, 127.68, 122.60, 121.22, 115.98, 114.04, 112.12, 76.37, 72.04, 55.98, 55.44. **HRMS** (ESI) m/z calculated for $C_{16}H_{18}O_4Na^+$ ($[M+Na]^+$) 298.11309, found 298.11340. Spectral data are in accordance with those previously reported.[30]

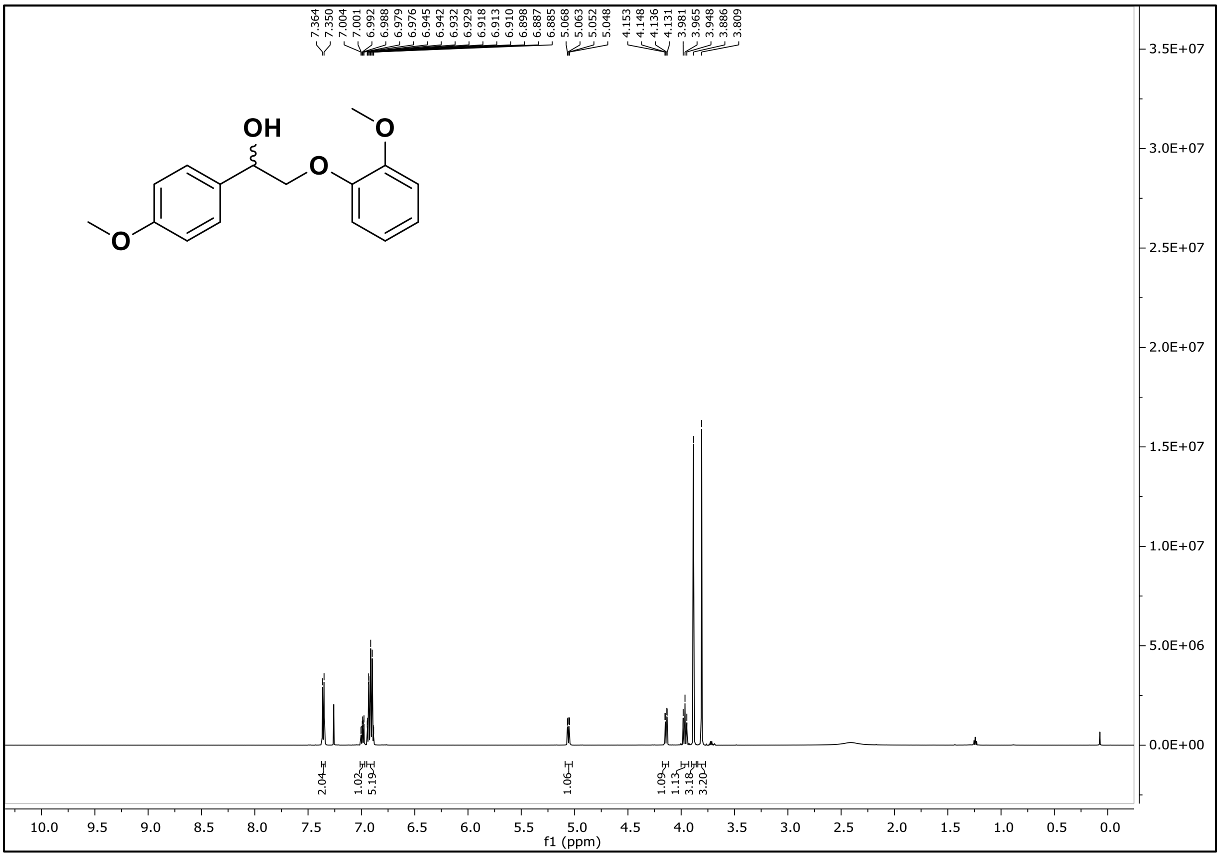

^{1}H NMR spectrum of **1a**.

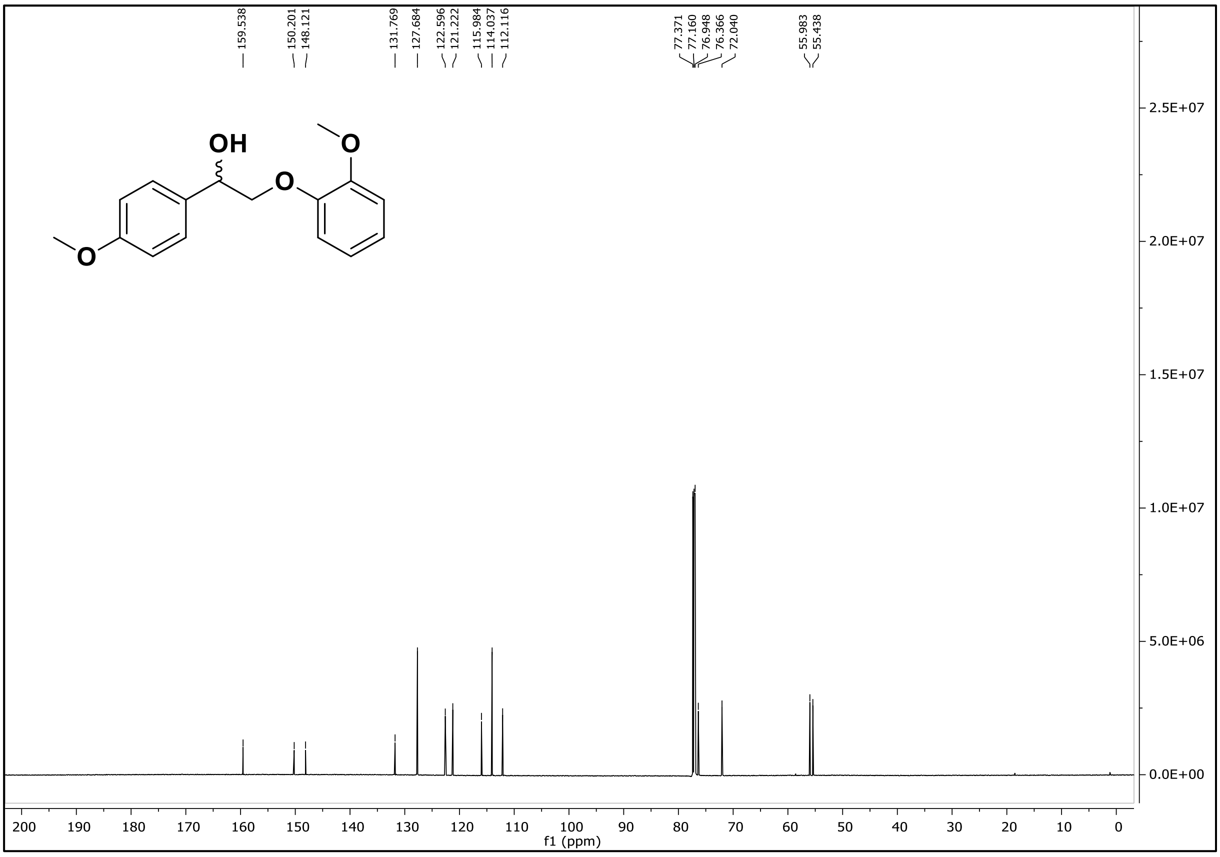

^{13}C NMR spectrum of **1a**.

3.1.3 Compound Erythro/Threo-2-(2-methoxyphenoxy)-1-(4-methoxyphenyl)propane-1,3-diol (1b)

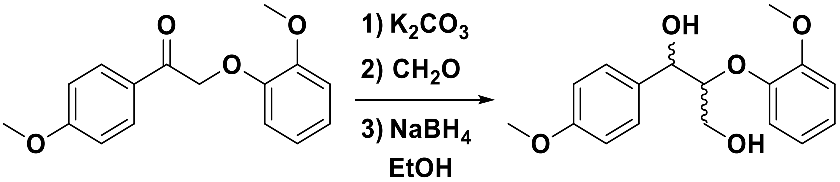

Erythro/Threo-2-(2-methoxyphenoxy)-1-(4-methoxyphenyl)propane-1,3-diol (**1b**) was prepared according to a literature procedure.[30] To a stirring suspension of K_2CO_3 (2.8, 20.2 mmol) in ethanol (200 mL, 99.5%) and 2-(2-methoxyphenoxy)-1-(4-methoxyphenyl)ethanone (5g, 18.5 mmol) at room temperature, 2.25 mL (27.5 mmol) water solution (36.5-38%) of formaldehyde was added. The reaction was monitored by TLC, after full consumption of starting material, $NaBH_4$ (2.5g, 66 mmol) was added and the reaction mixture was stirred for 2 h at room temperature. The reaction mixture was concentrated *in vacuo*. And then quenched by slow addition of NH_4Cl saturated water solution (100 mL) and extracted with diethyl ether (2 × 100 mL). The combined organic extracts were washed with brine and dried over anhydrous $MgSO_4$, filtered and evaporated to give a crude oil. The oil was purified by column chromatography (gradient

pentane: ethyl acetate, 1:1 to ethyl acetate) to give mixture of erythro- and threo-diastereomers (diastereomer ratio 1:1) of 2-(2-methoxyphenoxy)-1-(4-methoxyphenyl)propane-1,3-diol as an oil (4.94 g, 16.3 mmol) in 88% yield.

Erythro and threo: **^{1}H NMR**($CDCl_3$, 600 MHz): δ 7.35-7.28 (m, 4H), 7.11 (dd, *J* = 7.9 Hz, *J* = 1.6 Hz, 1H), 7.05-6.99 (m, 2H), 6.93 – 6.84 (m, 9H), 4.96 (dd, *J* = 6.4 Hz, *J* = 1.6 Hz, 2H), 4.14 (td, *J* = 5.4 Hz, *J* = 3.4 Hz, 1H), 4.05 (ddd, *J* = 7.7 Hz, *J* =4.1 Hz, *J* =3.3 Hz, 1H), 3.91-3.80 (m, 7H), 3.77 (d, *J* = 1.8 Hz, 6H), 3.66 (dd, *J* = 12.2 Hz, *J* = 3.4 Hz, 1H), 3.60 (dd, *J* = 12.5 Hz, *J* = 3.3 Hz, 1H), 3.44 (dd, *J* = 12.5 Hz, *J* = 4.1 Hz, 1H).**^{13}C NMR** ($CDCl_3$, 151 MHz): δ 159.47, 159.07, 151.42, 151.16, 147.72, 147.03, 132.37, 131.89, 128.33, 127.45, 127.43, 124.01, 123.90, 121.65, 121.57, 120.73, 120.55, 120.54, 113.93, 113.79, 112.25, 112.19, 89.11, 86.95, 73.58, 72.67, 60.99, 60.74, 55.88, 55.27. **HRMS** (ESI) m/z calculated for $C_{17}H_{20}O_5Na^+$ ($[M+Na]^+$) 328.12365, found 328.12407. Spectral data are in accordance with those previously reported.[30] Compound 1-(3,4-dimethoxyphenyl)-2-(2-methoxyphenoxy)propane-1,3-diol (**1c**) and compound 1-(4-hydroxy-3-methoxyphenyl)-2-(2-methoxyphenoxy)propane-1,3-diol (**1d**) were also prepared as previously reported.[31,32]

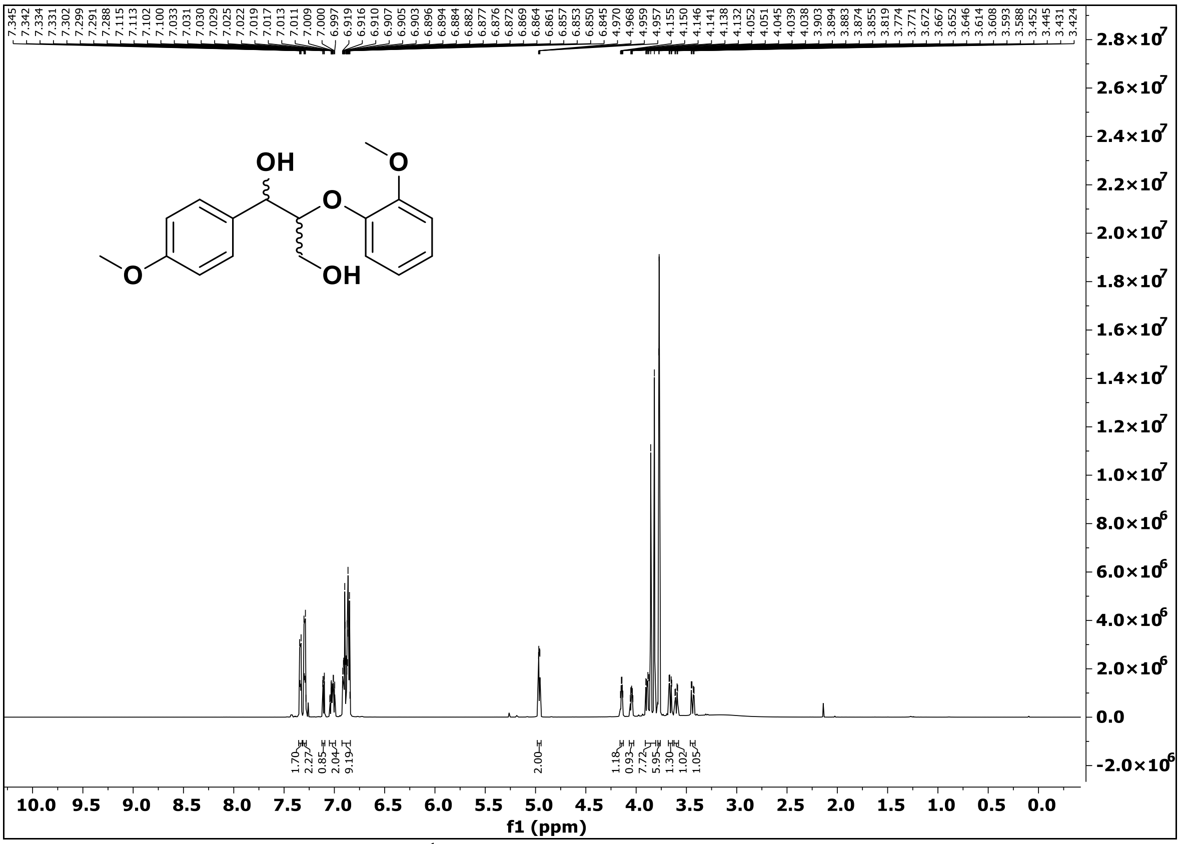

^{1}H NMR spectrum of **1b**.

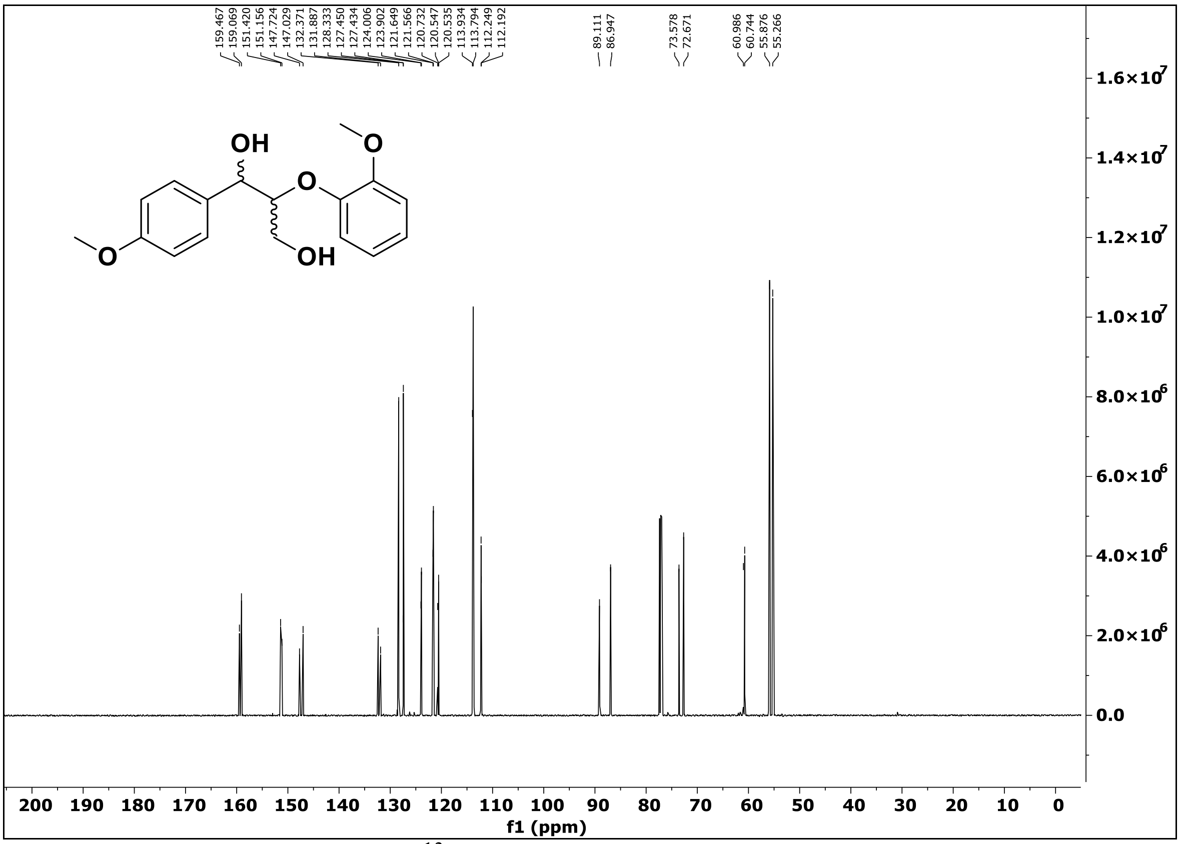

^{13}C NMR spectrum of **1b**.

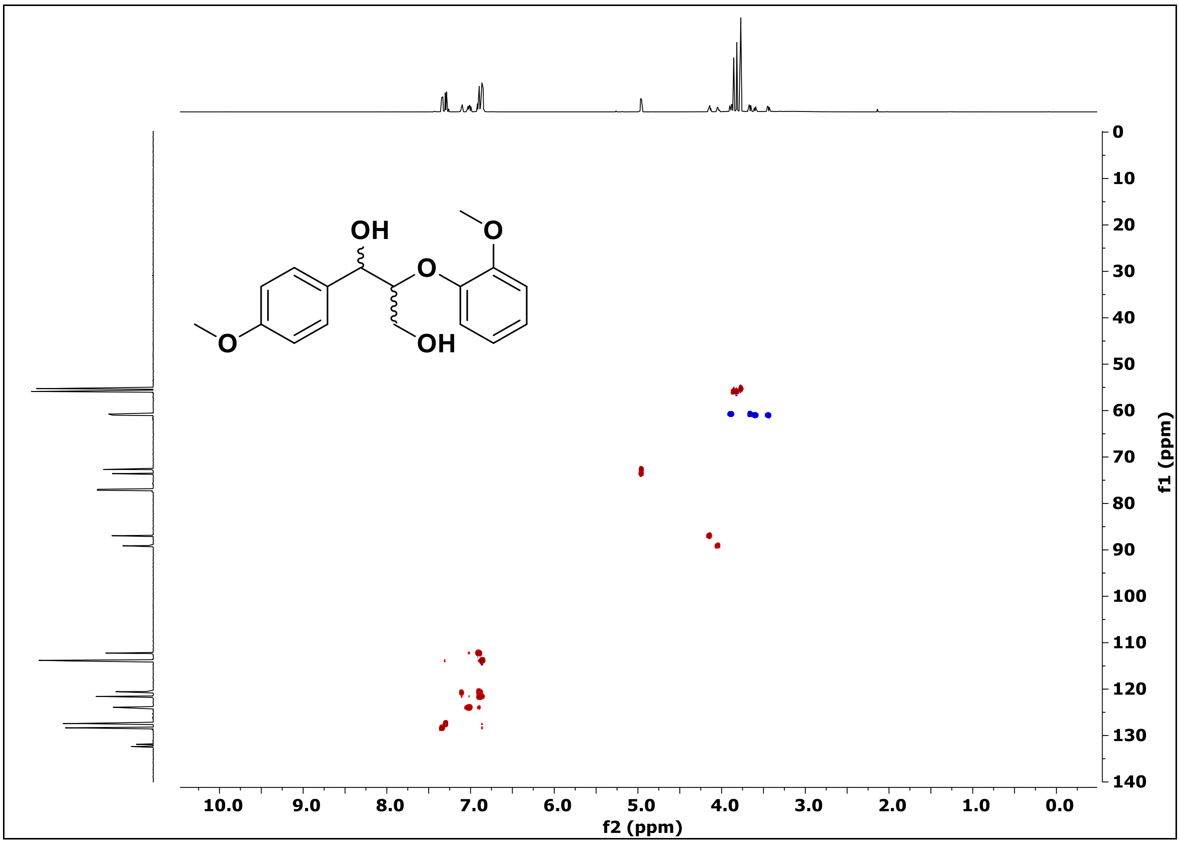

2D HSQC NMR spectrum of **1b**.

3.1.4 Compound 2-(2-(2-methoxyphenoxy)-1-(4-methoxyphenyl)ethoxy)ethanol (4aa)

The mixture of products was obtained after extraction workup with DCM and water. The crude mixture was purified by column chromatography (gradient pentane: ethyl acetate, 1:1 to ethyl acetate) to give 2-(2-(2-methoxyphenoxy)-1-(4-methoxyphenyl)ethoxy)ethanol as an oil in 70% yield.

^{1}H NMR(CDCl$_3$, 600 MHz): δ 7.21 (d, *J* = 8.4 Hz, 2H), 6.81–6.75 (m, 6H), 4.70 (dd, *J* = 9.0 Hz, *J* = 3.0 Hz, 1H), 4.00 (t, *J* = 9.6 Hz, 1H), 3.96 (dd, *J* = 10.2 Hz, *J* = 3.0 Hz, 1H), 3.75 (s, 3H), 3.69 (s, 3H), 3.68-3.56 (m, 3H), 3.41-3.38 (m, 1H), 3.21 (*br* s, 1H). **^{13}C NMR** (CDCl$_3$, 151 MHz): δ 159.57, 149.42, 148.15, 130.49, 127.97, 121.50, 120.82, 114.01, 113.67, 111.83, 80.37, 73.43, 70.72, 61.63, 55.74, 55.24. **HRMS** (ESI) m/z calculated for $C_{18}H_{22}O_5Na^+$ ([M+Na]$^+$) 342.13930, found 342.13934.

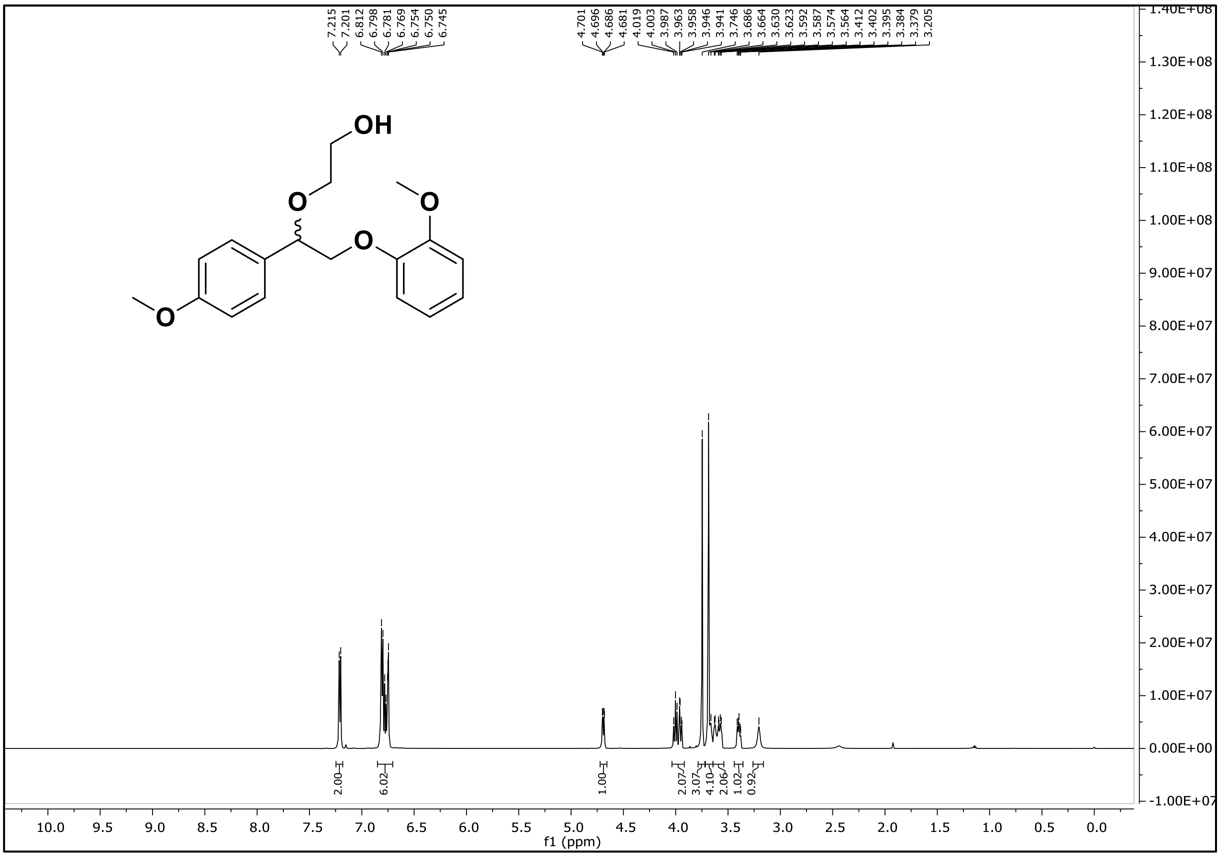

^{1}H NMR spectrum of **4aa**.

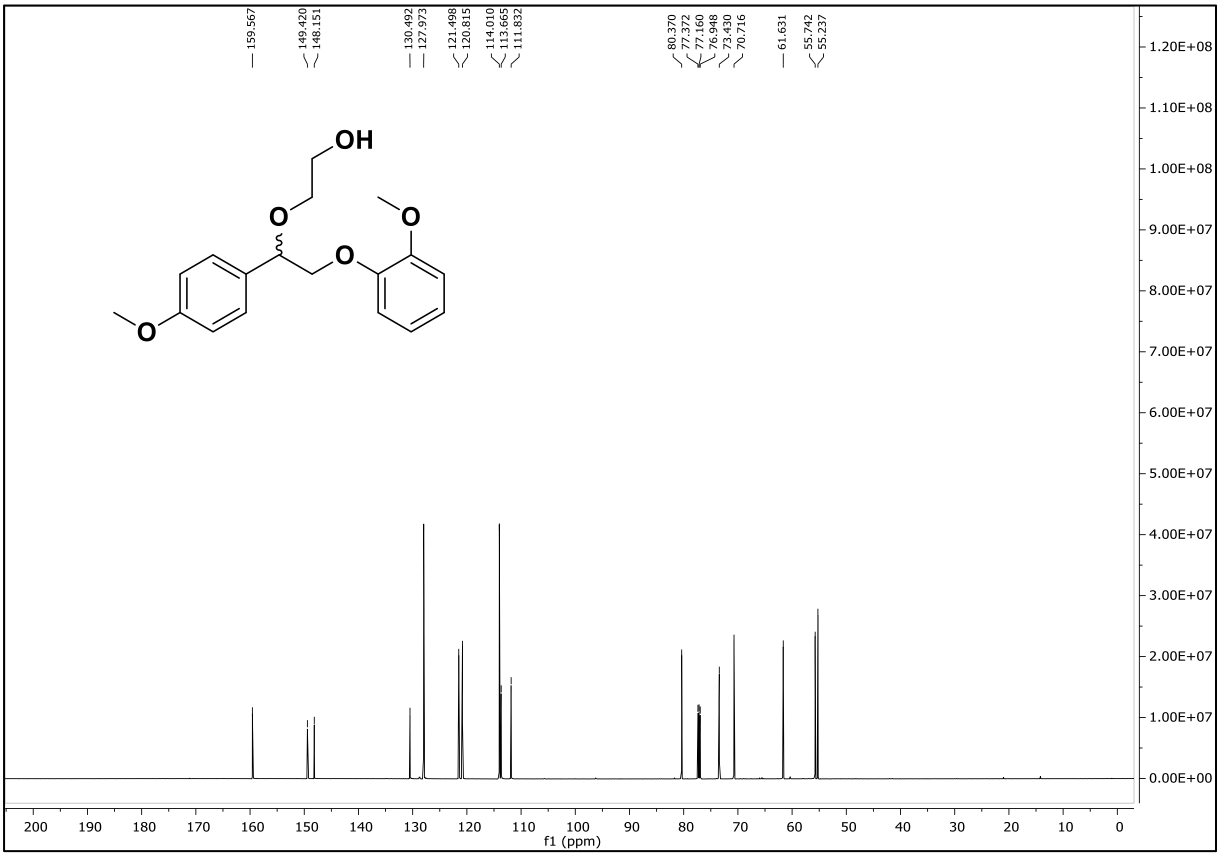

^{13}C NMR spectrum of **4aa**.

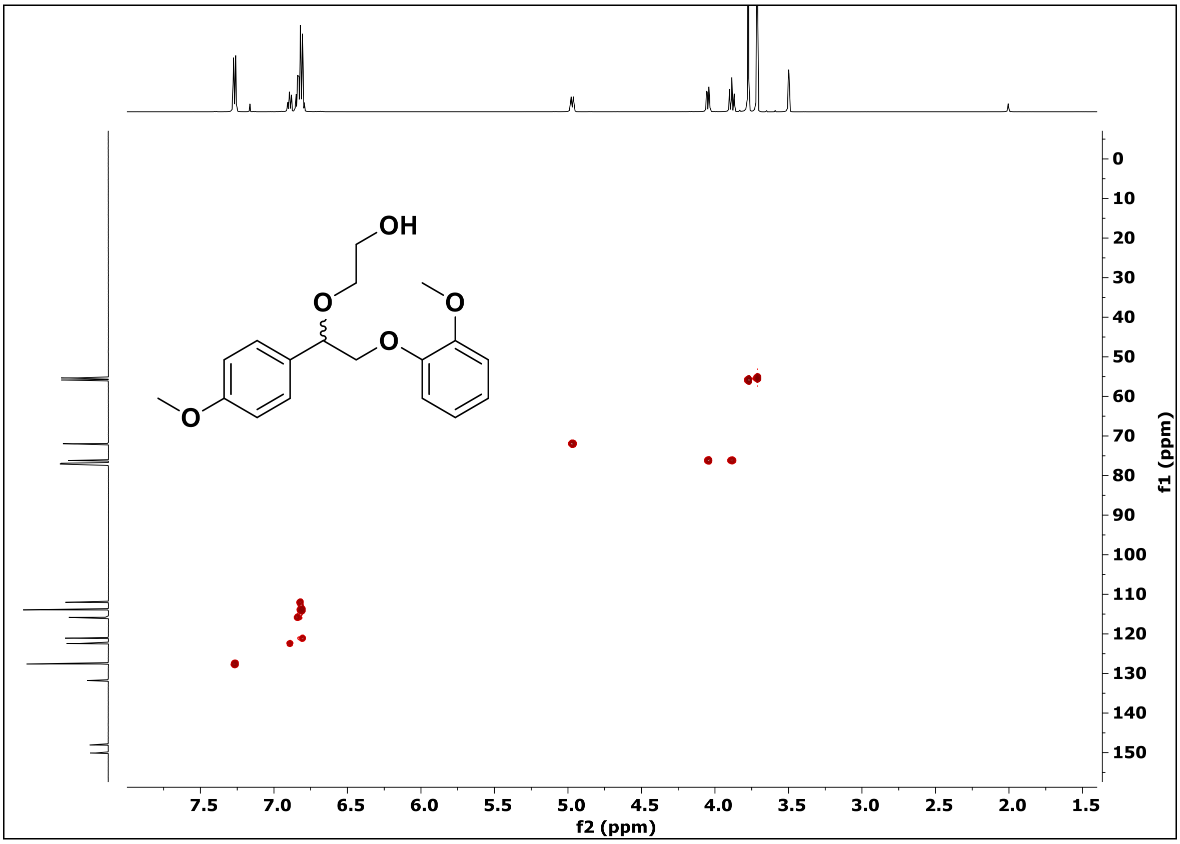

2D HSQC NMR spectrum of **4aa**.

3.1.5 Compound 3-(2-hydroxyethoxy)-2-(2-methoxyphenoxy)-3-(4-methoxyphenyl)propanol (4ba)

The mixture of products was obtained after extraction workup with dichloromethane and water. The crude mixture was purified by column chromatography (methanol: dichloromethane, 1:99) to give **4ba** as an oil in 70% yield.

Erythro and threo: **^{1}H NMR**($CDCl_3$, 600 MHz):δ 7.32 (dd, *J* = 15.3, *J* = 8.6 Hz, 4H),7.13 (dd, *J* = 8.3, *J* = 1.5 Hz, 1H), 7.01-6.82 (m, 9H), 6.77 (td, *J* = 7.8, *J* = 1.5 Hz, 1H), 6.60 (dd, J = 8.0, *J* = 1.5 Hz, 1H), 4.67 (dd, J = 26.5, *J* = 7.3 Hz, 2H), 4.27 (ddd, J = 7.7, *J* = 5.3, *J* = 3.5 Hz, 1H), 4.12 (ddd, J = 7.0, *J* = 3.9, *J* = 3.0 Hz, 1H), 3.98 (dd, J = 12.2, *J* = 4.0 Hz, 1H), 3.85 (m, 3H), 3.82-3.72 (m, 11H), 3.71-3.59 (m, 4H), 3.52 (m, 2H), 3.45-3.40 (m, 3H), 3.03 (*br* s, 4H);**^{13}C NMR** ($CDCl_3$, 151 MHz): δ 159.72, 159.43, 151.24, 150.75, 148.44, 147.21, 131.16, 130.21, 128.76, 128.68, 123.50, 123.19, 121.49, 121.37, 119.83, 119.35, 114.13, 113.87, 112.27, 112.21, 86.73, 86.15, 82.29, 80.35, 70.50, 70.38, 61.76, 61.65, 61.52, 60.87, 55.90, 55.88, 55.31; **HRMS** (ESI) m/z calculated for $C_{19}H_{24}O_6Na^+$ ($[M+Na]^+$) 371.14651, found 371.14681.

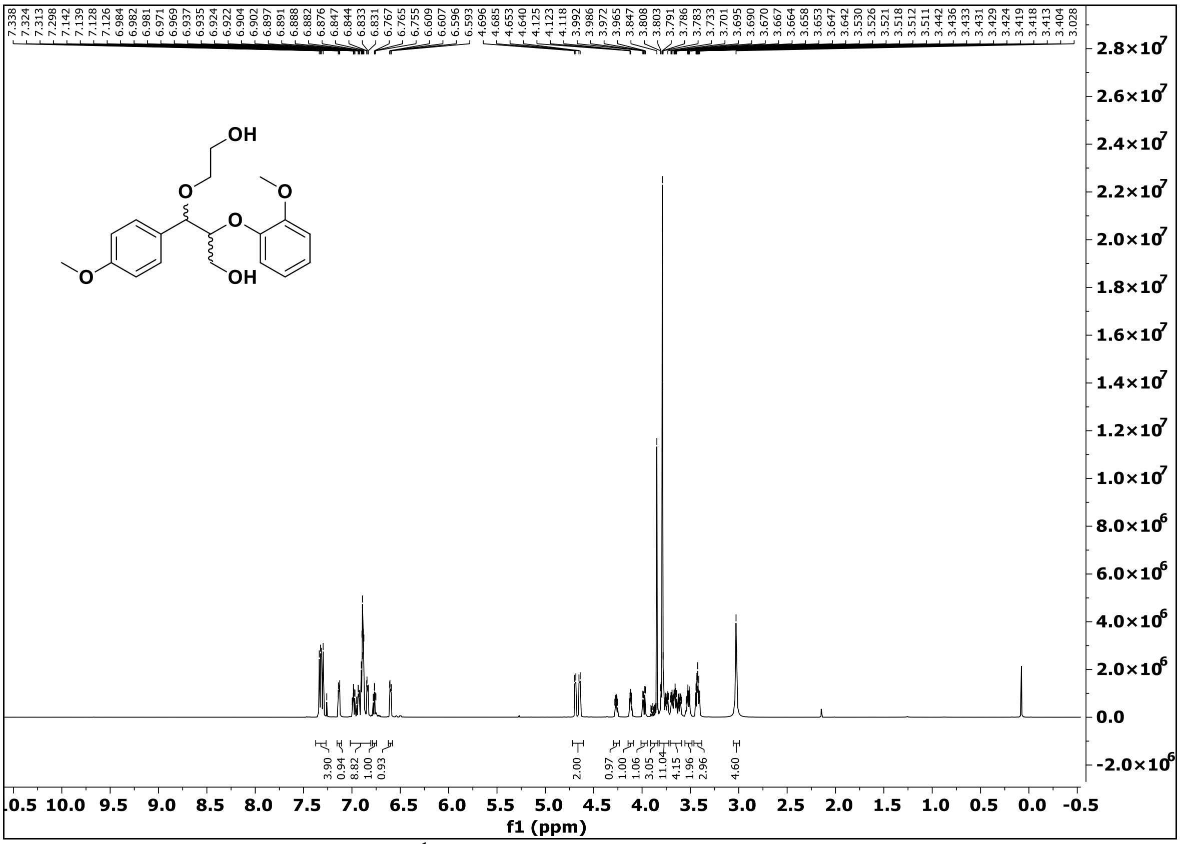

^{1}H NMR spectrum of **4ba**.

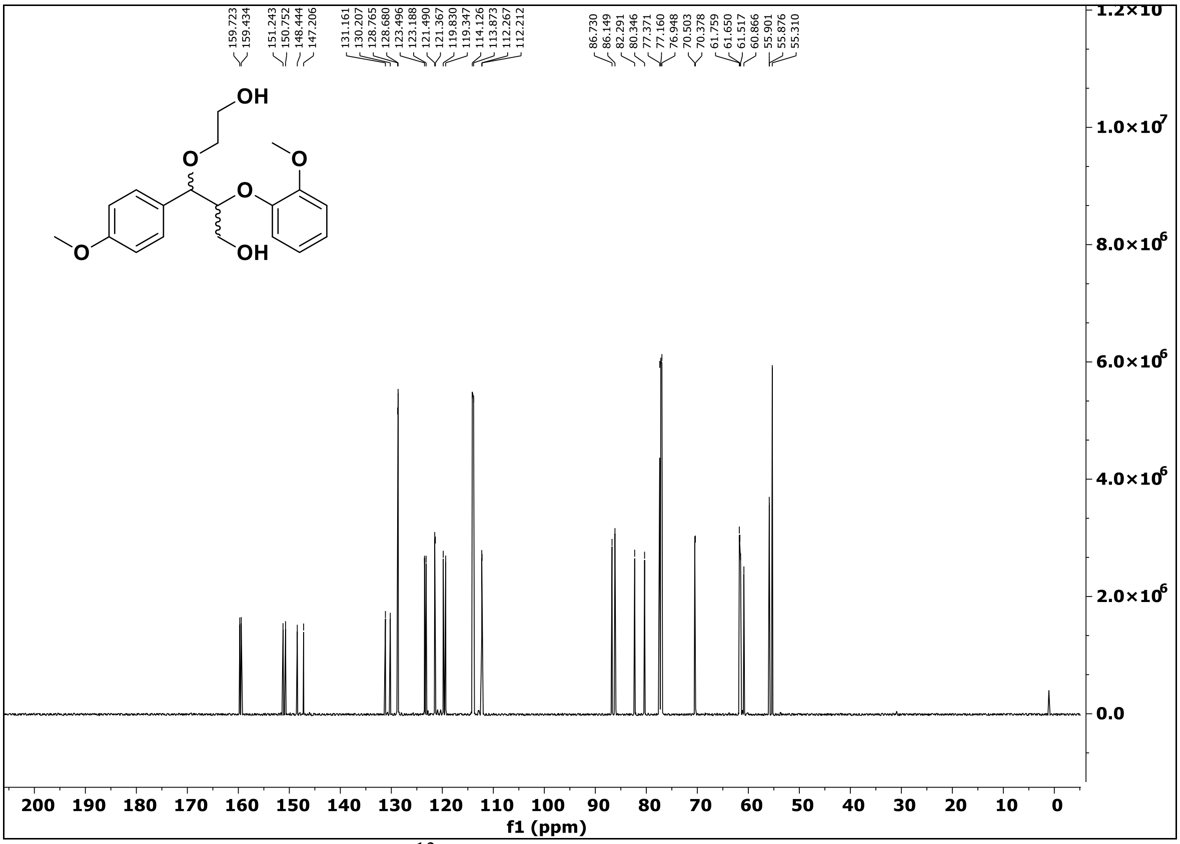

^{13}C NMR spectrum of **4ba**.

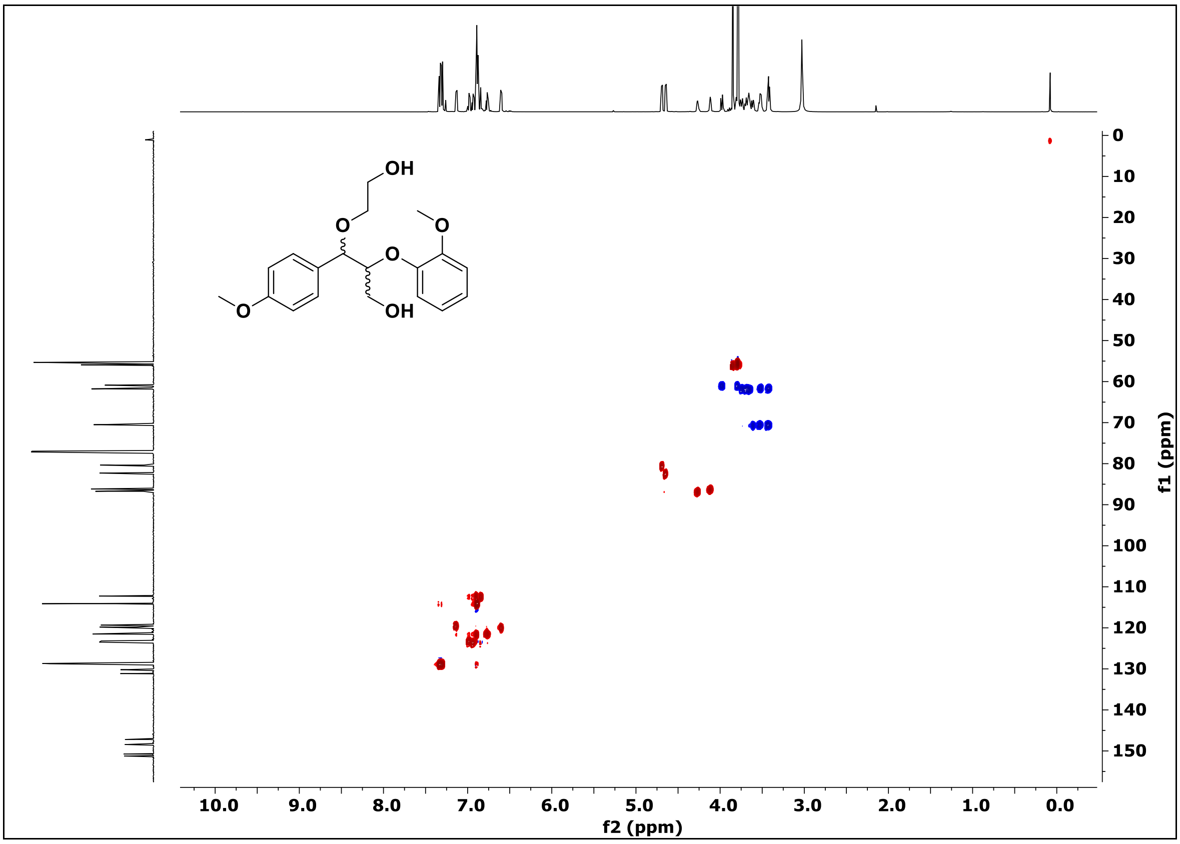

2D HSQC NMR spectrum of **4ba**.

3.1.6. Compound3-(1-(4-hydroxy-3-methoxyphenyl)-2-(2-methoxyphenoxy)ethoxy)propane-1,2-diol (4ab) and 2-(1-(4-hydroxy-3-methoxyphenyl)-2-(2-methoxyphenoxy)ethoxy)propane-1,3-diol(4ab′)

The mixture of products was obtained after extraction workup with dichloromethane and water. The crude mixture containing **4ab** and **4ab′** was purified by column chromatography (methanol: dichloromethane, 1:99) to give a mixture of **4ab** and **4ab′**as an oil in 73% yield. **4ab** and **4ab′** were identified separately by NMR.

4ab: Erythro and threo: **^{1}H NMR** ($CDCl_3$, 600 MHz): δ 7.29 (dd, *J* = 8.6, *J* = 3.4 Hz, 4H), 6.96-6.84 (m, 12H), 4.80-4.76 (m, 2H), 4.14-4.04 (m, 4H), 3.95-3.89 (m, 2H), 3.87 (d, *J* = 1.0 Hz, 6H), 3.81 (d, *J* = 0.8 Hz, 6H), 3.72-3.60 (m, 5H), 3.57 (dd, *J* = 11.5, 5.2 Hz, 1H), 3.51 (dd, *J* = 10.8, *J* = 3.9 Hz, 1H), 3.43 (dd, *J* = 10.3, *J* = 7.4 Hz, 1H); **^{13}C NMR** ($CDCl_3$, 151 MHz): δ 159.84, 159.82, 149.68, 149.60, 148.27, 148.22, 130.25, 130.18, 128.13, 128.08, 121.81, 121.79, 121.01, 114.24, 114.08, 113.94, 112.11, 112.08, 82.06, 80.75, 73.68, 73.52, 71.87, 71.33, 70.74, 70.20, 64.08, 63.76, 55.98, 55.96, 55.43.**HRMS** (ESI) m/z calculated for $C_{19}H_{24}O_7Na^+$ ($[M+Na]^+$) 371.14651, found 371.14691.

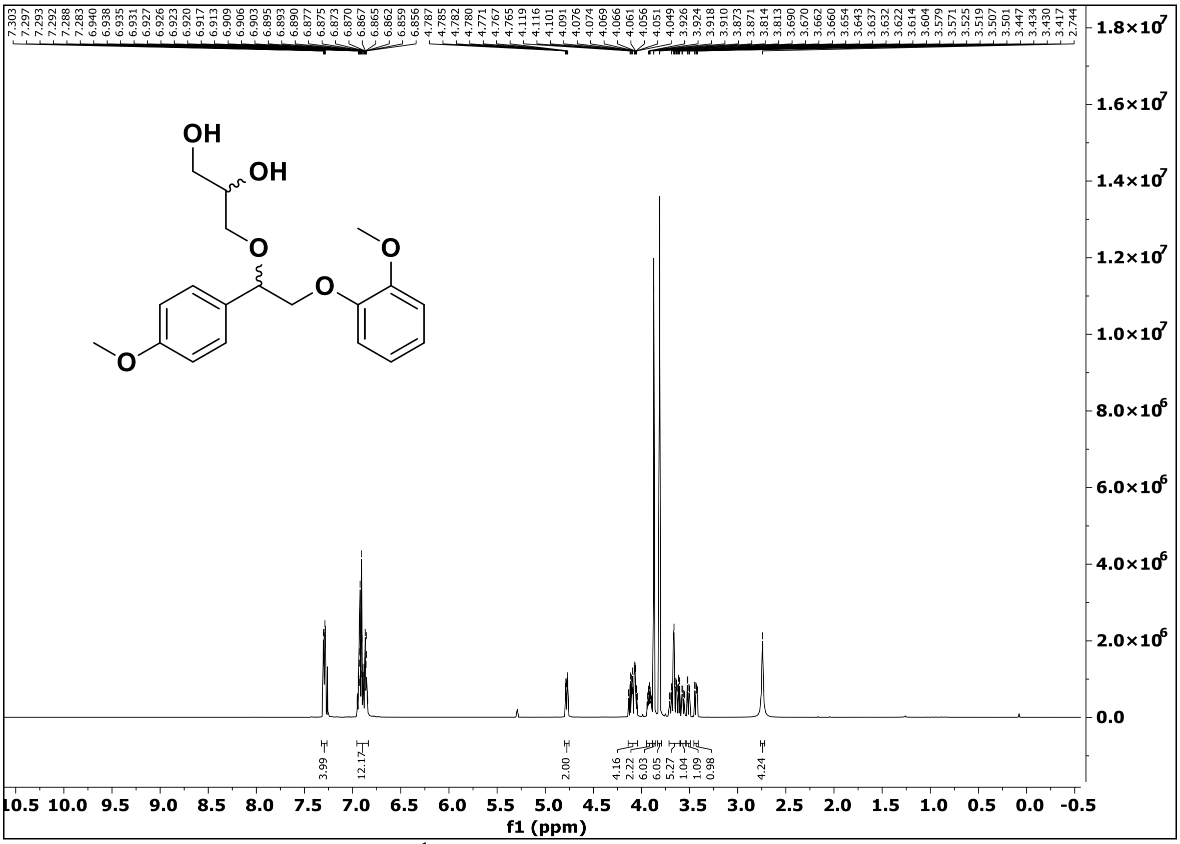

^{1}H NMR spectrum of **4ab**.

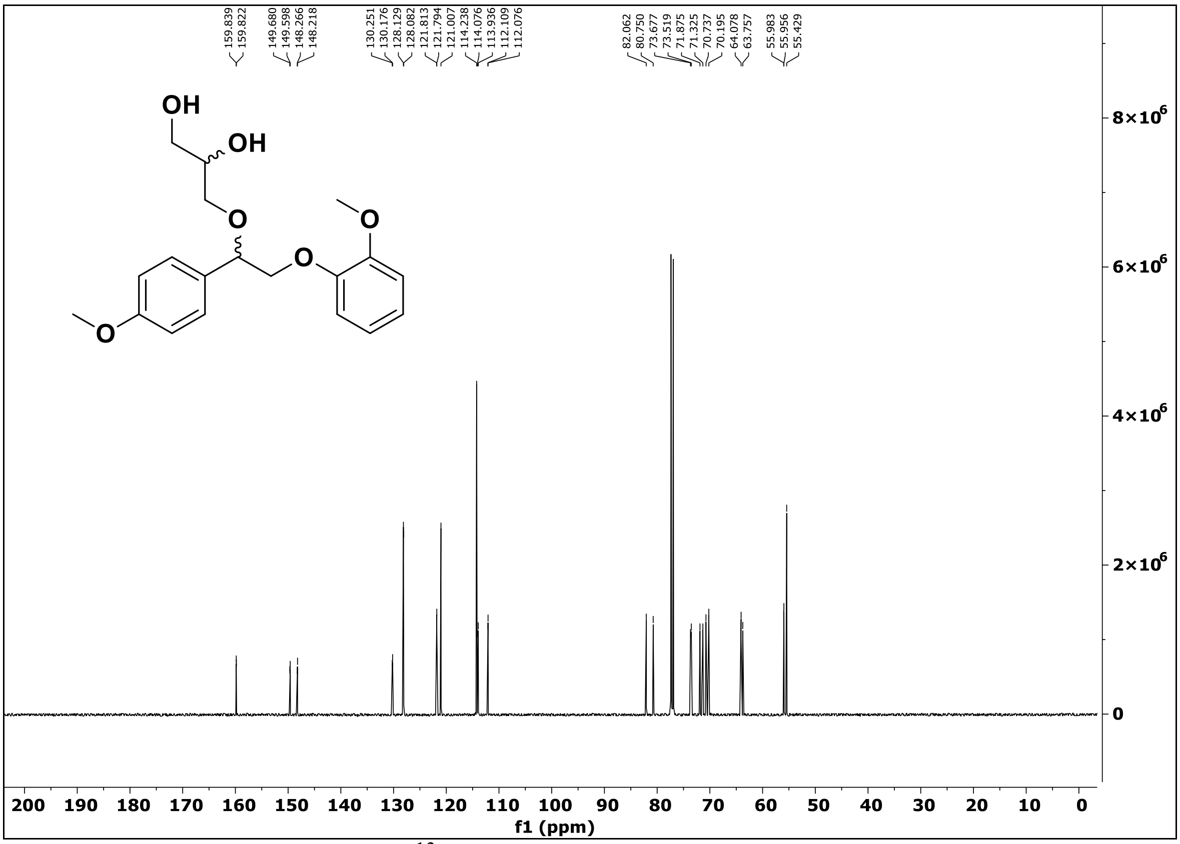

^{13}C NMR spectrum of **4ab**.

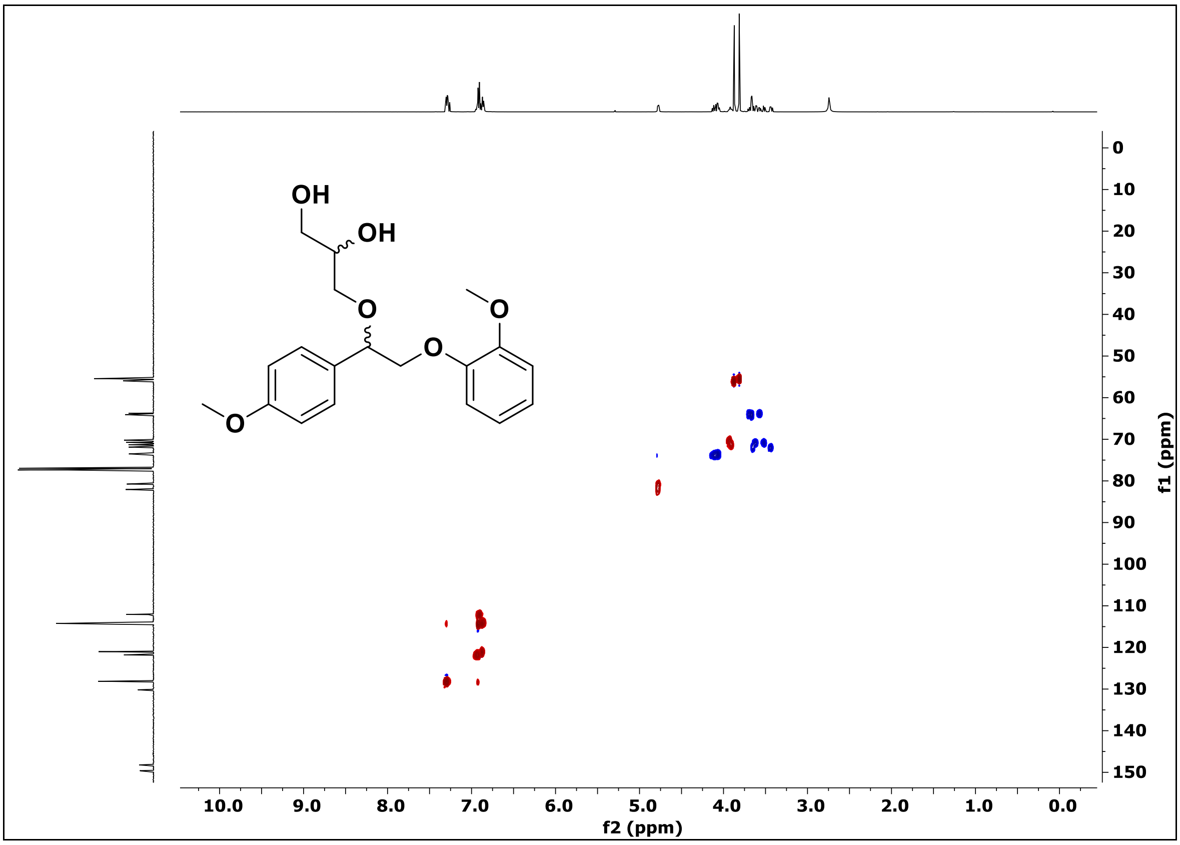

2D HSQC NMR spectrum of **4ab**.

4ab′:**^{1}H NMR**(CDCl$_3$, 600 MHz):δ 7.28 (d, *J* = 8.6 Hz, 2H), 6.88-6.78 (m, 5H), 6.74 (dd, *J* = 8.0, *J* = 1.5 Hz, 1H), 4.89 (dd, *J* = 9.9, *J* = 2.9 Hz, 1H), 4.02 (m, 2H), 3.85 (dd, *J* = 12.3, *J* = 2.3 Hz, 1H), 3.81 (s, 3H), 3.75 (s, 3H), 3.63 (dd, *J* = 12.4, *J* = 5.0 Hz, 1H), 3.60-3.54 (m, 2H), 3.48 (q, *J* = 6.8 Hz, 1H); **^{13}C NMR** (CDCl$_3$, 151 MHz): δ 159.91, 149.06, 147.87, 130.71, 127.94, 121.46, 120.80,114.39, 112.62, 111.40, 80.61, 79.98, 72.80, 63.15, 62.14, 55.59, 55.33; **HRMS** (ESI) m/z calculated for $C_{19}H_{24}O_7Na^+$ ([M+Na]$^+$) 371.14651, found 371.14702.

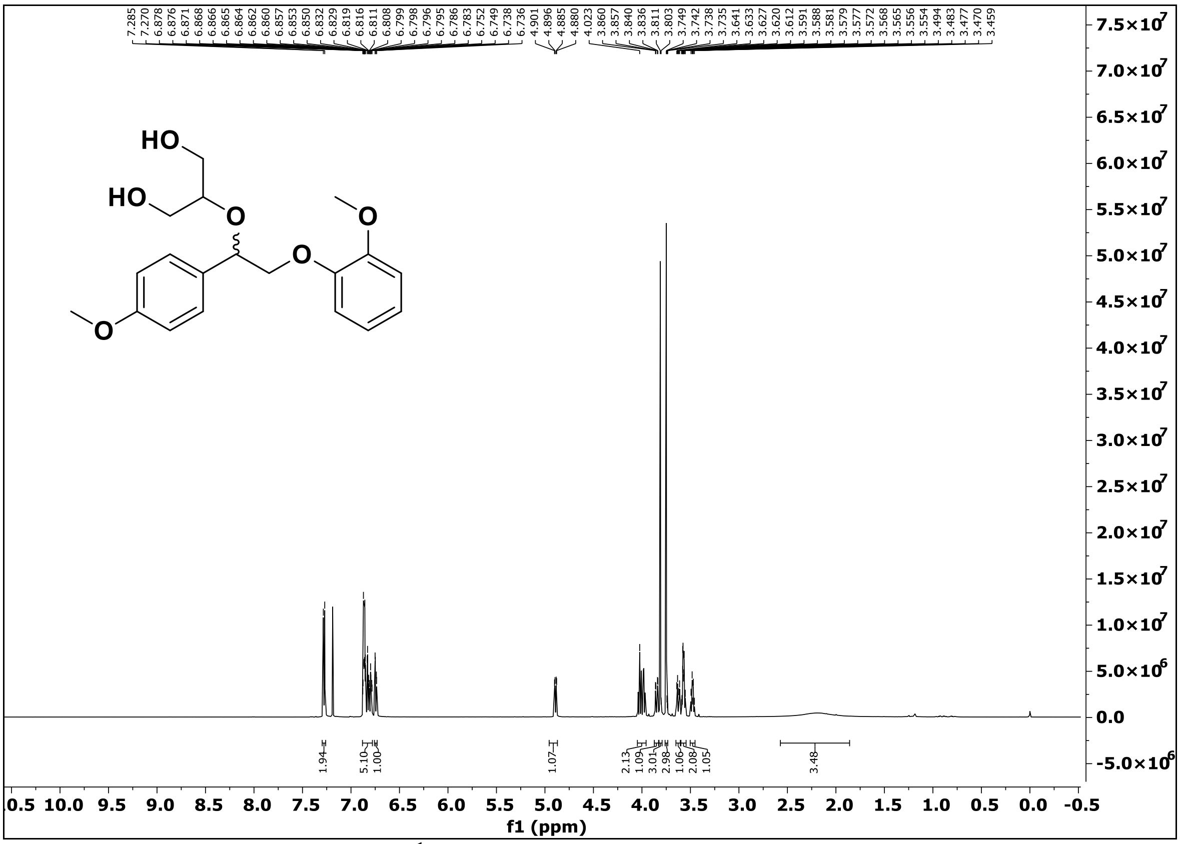

^{1}H NMR spectrum of **4ab'**.

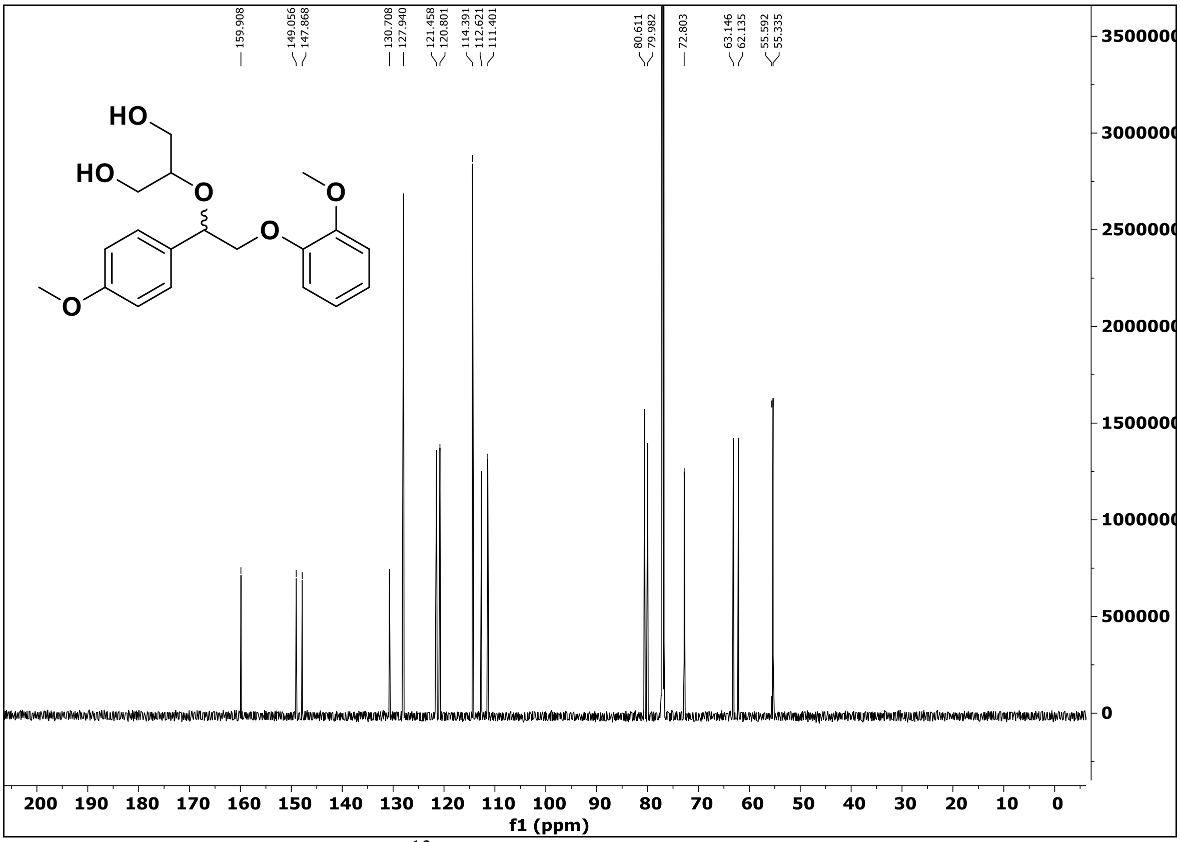

^{13}C NMR spectrum of **4ab'**.

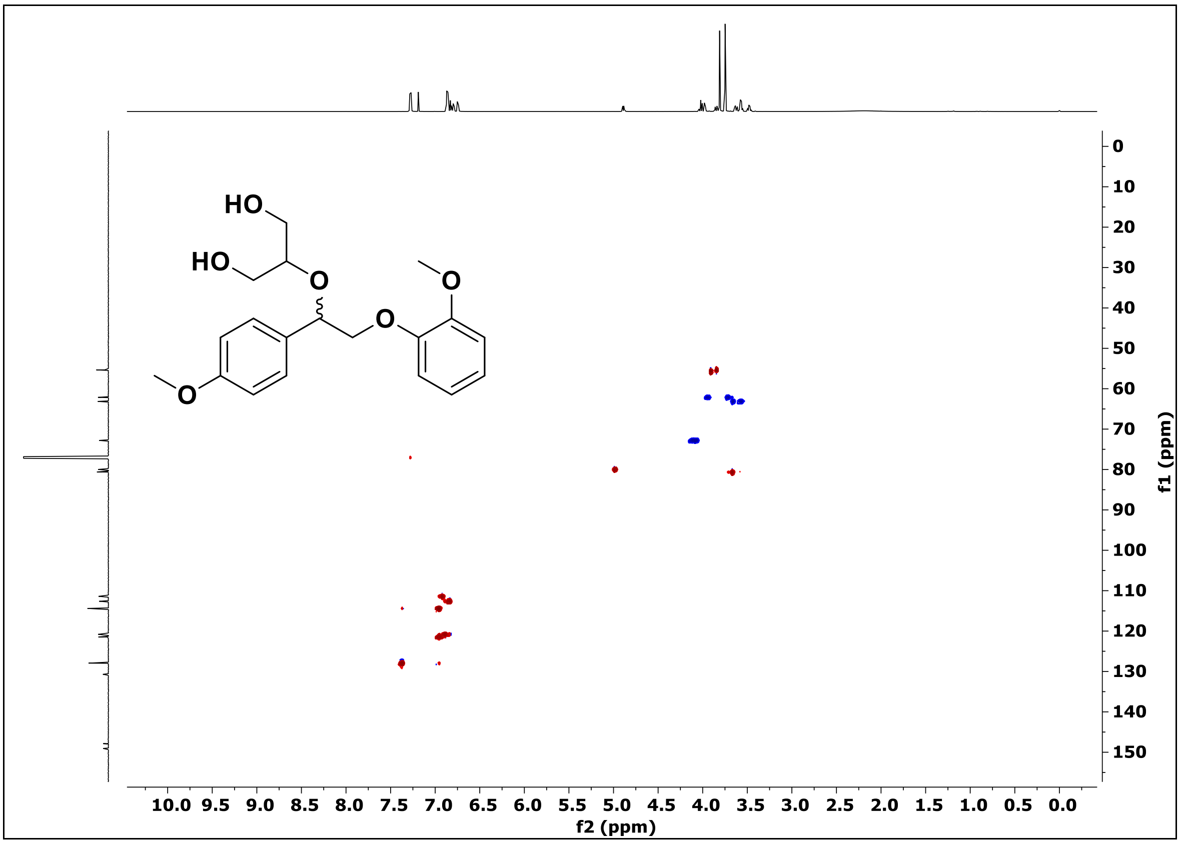

2D HSQC NMR spectrum of **4ab'**.

3.1.7 Compound4-(3-hydroxy-1-(2-hydroxyethoxy)-2-(2-methoxyphenoxy)propyl)-2-methoxyphenol (4da)

The mixture of products was obtained after extraction workup with dichloromethane and water. The crude mixture containing **4da** was purified by column chromatography (methanol: dichloromethane, 2:98) to give a mixture of **4da**as an oil in 82% yield.**4da** were identified separately by NMR.

Erythro and threo: **^{1}H NMR**(CDCl$_3$, 600 MHz): δ 7.08 (dd, *J* = 8.3, *J* = 1.6 Hz, 1H), 6.96-6.92 (m, 1H), 6.91-6.78 (m, 10H), 6.74-6.70 (m, 1H), 6.52 (dd, *J* = 8.0, *J* =1.6 Hz, 1H), 5.65 (*br*s, 2H), 4.60 (d, *J* = 7.1 Hz, 1H), 4.54 (d, *J* = 7.8 Hz, 1H), 4.20-4.14 (m, 1H), 4.04 (ddd, *J* = 7.0, *J* = 3.8, *J* = 3.0 Hz, 1H), 3.91 (dd, *J* = 12.3, *J* = 3.9 Hz, 1H), 3.84-3.68 (m, 15H), 3.68-3.56 (m, 2H), 3.54-3.48 (m, 1H), 3.43-3.34 (m, 3H), 2.42 (*br*s, 4H).**^{13}C NMR** (CDCl$_3$, 151 MHz):δ 151.26, 150.83, 148.38, 147.15, 146.93, 146.65, 145.84, 145.46, 131.03, 130.00, 123.59, 123.35, 121.51, 121.36, 120.72, 120.52, 119.93, 119.58, 114.45, 114.29, 112.16, 112.13, 109.84, 109.51, 87.03, 86.35, 82.75, 80.59, 70.49, 70.38, 61.75, 61.70, 61.59, 60.95, 56.03, 56.01, 55.88, 55.82.**HRMS** (ESI) m/z calculated for $C_{19}H_{22}O_7Na^+$ ([M+Na]$^+$) 387.14142, found 387.14203.

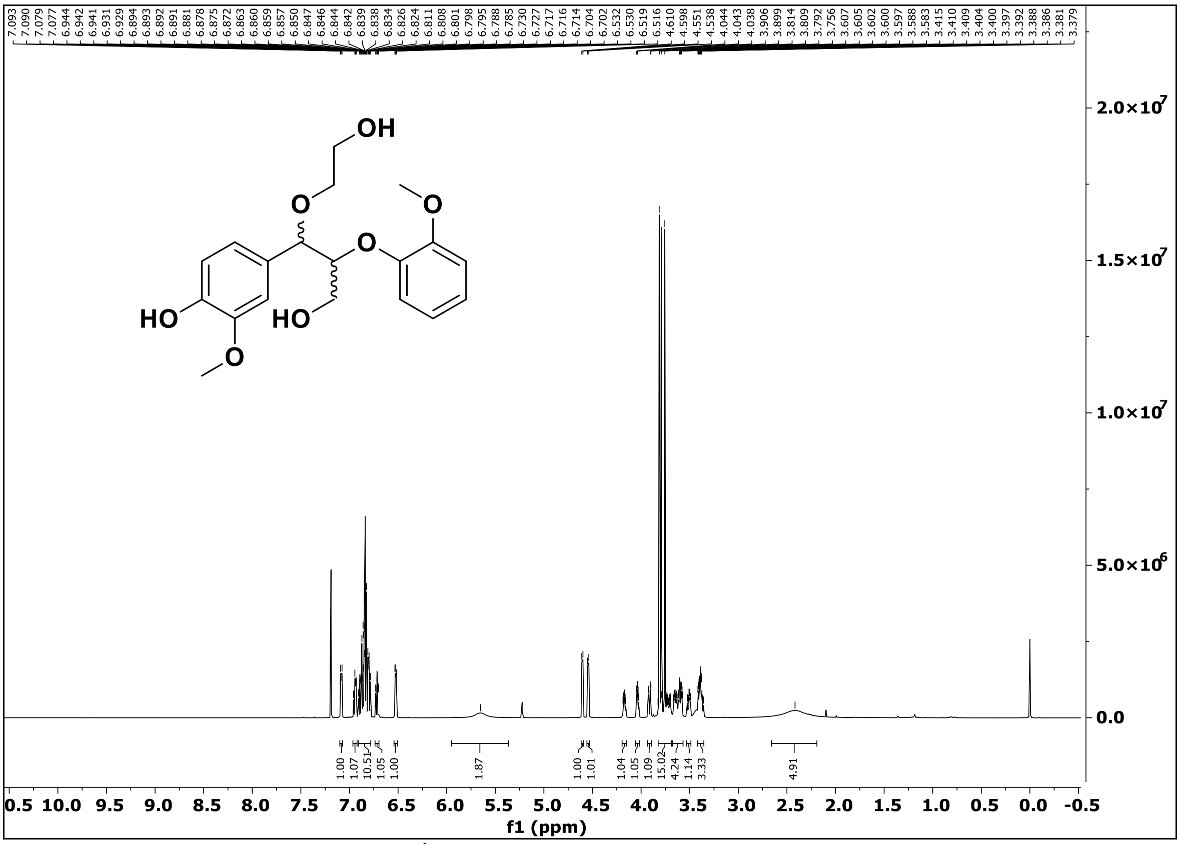

^{1}H NMR spectrum of **4da**.

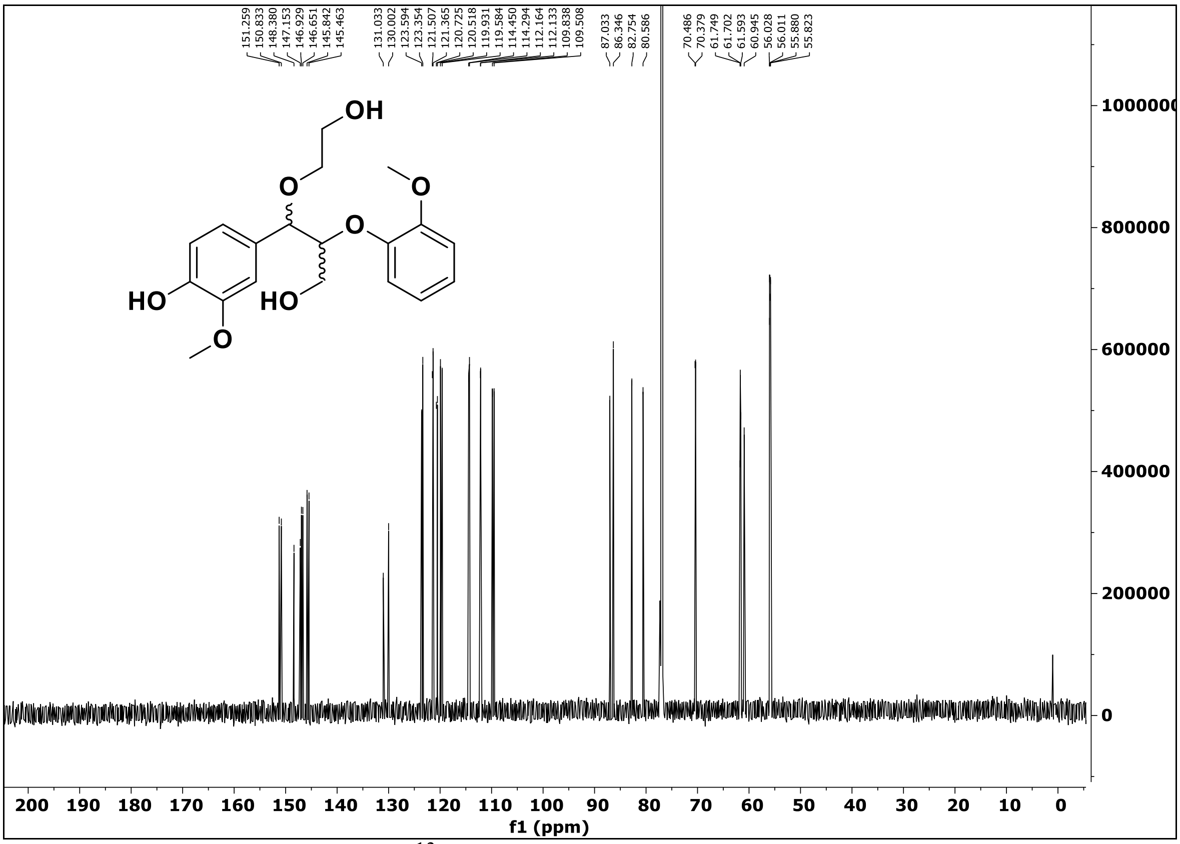

^{13}C NMR spectrum of **4da**.

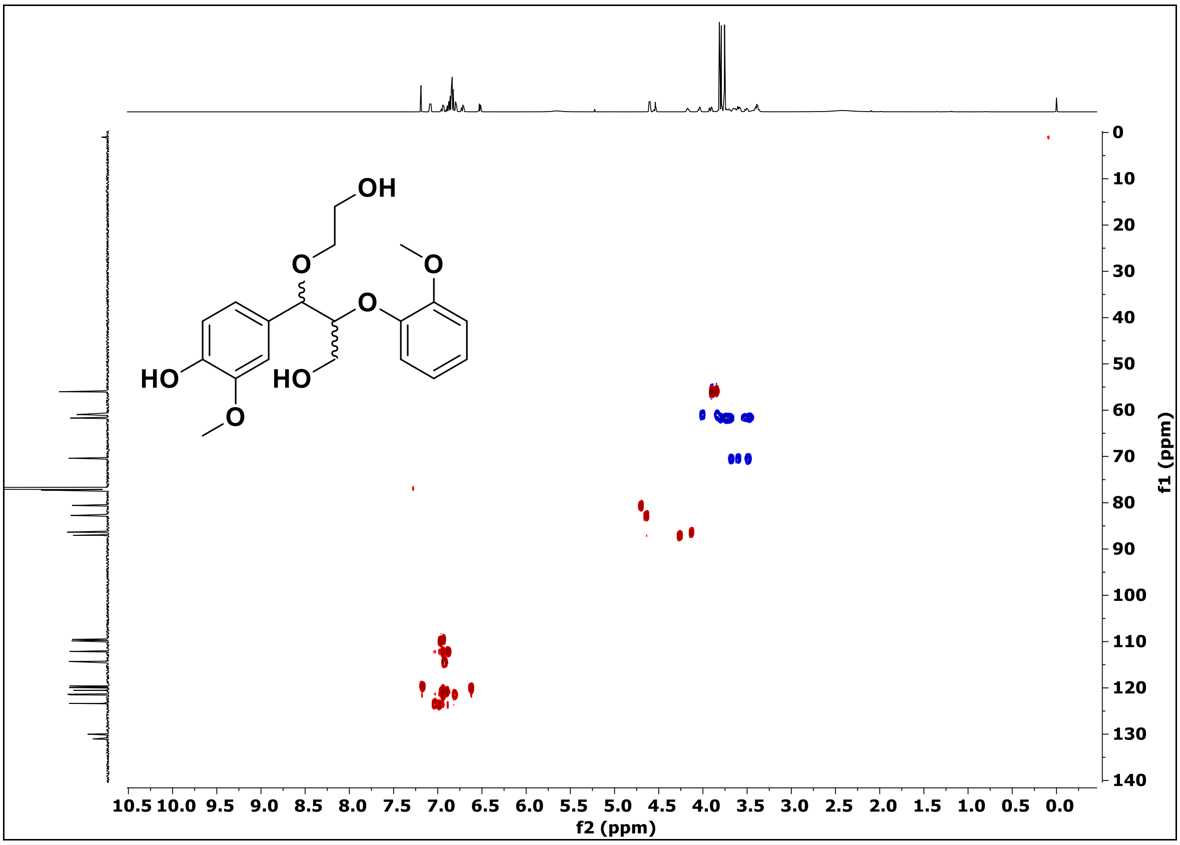

2D HSQC NMR spectrum of **4da**.

Supplementary Note 4: In-depth studies regarding the nature of DES

4.1 Elucidating the nature of interactions between the ternary DES compositions

4.1.1 Analysis of three different DES compositions by diverse NMR spectroscopy techniques

In our study, different DES mixtures, namely: ChCl:OA (1:1), ChCl:EG (1:2), ChCl:OA:EG (1:1:2) were prepared and analyzed by NMR and IR spectroscopy both in neat form and dissolved in a deuterated solvent (D_2O and DMSO). ^1H, ^{13}C, COSY, HSQC, HMBC, NOESY and DOSY experiments were realized.

Supplementary Figure 19 shows the ^1H NMR of the DES mixtures. The signals for all the aliphatic protons of the components can be identified in both the ^1H and ^{13}C NMR spectra. In all cases, for the protons belonging to the hydroxyl groups of ChCl and EG (4 – ChCl, 7 – EG) and carboxylic group of OA (5), respectively, only one signal can be seen in the NMR spectra recorded on the neat DES. This coalescence of the protons belonging to a hydroxyl group (including water) can also be seen in the literature in the case when water is present in the system. In the present case, DES was prepared starting from dry choline chloride and anhydrous oxalic acid and ethylene glycol, but water is generated by the ester formation side reaction between the oxalic acid and choline chloride or ethylene glycol. The ester formation was previously shown in the literature.[33,34]

In the NOESY spectrum (Supplementary Figure 20) of ChCl:OA:EG (1:1:2, neat) several correlations between the peak corresponding to the hydroxyl groups and the rest of the molecules, the -CH_2- groups of ChCl and EG and CH_3 of ChCl, can be observed (green oval). Due to the fact that all –OH and COOH overlap, these can also be intramolecular correlations and intermolecular correlations. Of particular interest is however a correlation between the peaks corresponding to the -CH_2- groups of EG (3.55 ppm) and the ones corresponding to the CH_3 groups of choline chloride (3.20 ppm) (marked with blue circle). This is strongly suggesting a steric proximity of these two components, supporting the involvement of EG in the ternary DES.

Supplementary Figure 21 shows the DOSY NMR spectrum of the ternary DES ChCl:OA:EG (1:1:2, neat).

According to the literature, the viscosities of the studied DES systems are as presented in the table below (Supplementary Table 8). Supplementary Table 8 also contains the diffusion

coefficients (D_{ChCl}) obtained from the DOSY experiments.

The diffusion coefficients (Supplementary Table 9), corresponding to the choline chloride, obtained from the DOSY experiments, are observed to be increasing with decreasing viscosity values known from the literature for the DES. The D_{ChCl} observed for the ternary DES ChCl:OA:EG is lower than the one for ChCl:EG and higher than for ChCl:OA, suggesting that an intermolecular interaction is present in the DES.

With the use of the Stokes-Einstein relation (Equation 1) more information can be obtained about the three studied DESs.

$$D = \frac{k_B T}{6\pi\eta r} \quad (1)$$

where *D* is diffusivity, *k* is the Boltzmann constant, *T* is the temperature, η is the viscosity, and *r* is the radius of the diffusing solute molecule.

From this, r will be Equation 2

$$r = \frac{k_B T}{6\pi\eta D} \quad (2)$$

The values obtained for the radius of the diffusing molecule suggest that the ternary mixture ChCl:OA:EG produces the biggest aggregate, followed by ChCl:EG and ChCl:OA, the smallest one. These values also show that the ternary DES forms an aggregate, rather than a mixture of the individual components, providing a further proof towards its existence.

In order to further support the presence of intermolecular interactions between all three components of the ternary DES ChCl:OA:EG, carbon DOSY NMR experiment was also realized on the neat DES (using D_2O capillary, Pulse Sequence dstebpgp3s, NS = 64, TD = 16). The carbon DOSY spectra (Supplementary Figure 22) shows that all the components have very similar diffusion coefficient, suggesting an interaction between the OA (signals around 160 ppm), EG and ChCl (signals between 50-80 ppm).

It should be noted, that NMR analysis cannot detect chloride ion-associated effects. Although DFT calculation (see Note 4.2) can observe the effect/role of chloride ions on the formation of DESs, this limitation exists for the NMR studies.

4.1.2 IR measurements of DES

The prepared DES samples were also analyzed by FTIR-ATR spectroscopy, the data is presented in Supplementary Figure 23.[9] The FTIR spectrum of the ternary DES ChCl:OA:EG (Supplementary Figure 23a – red, Supplementary Figure 23b – black) can be compared with data shown in the literature. Furthermore, characteristic bands for DESs can be observed, similar to those seen in binary mixtures (See Supplementary Figure 23).[36,37]

4.2 Computational studies related to different DES compositions

4.2.1 DFT calculation of DESs

Two type of DESs, namely ChCl/OA (1:1) and ChCl/EG (1:2) were used for calculation, to investigate the hydrogen bonding interactions. The optimized structure and parameters of complex (left) ChCl/OA and (right) ChCl/EG can be seen in Supplementary Figure 24, chloride ion forms a hydrogen bond with hydrogen of OA and hydroxy of EG after optimization, the bond length of them are 1.927 Å and 2.127 Å, respectively. These interatomic distances are all within the accepted criteria of H-bond coordination (<3.5 Å). The interaction energy can be defined as follows: $\triangle E=E_{A/B}-(E_A+E_B)$. $\triangle E$ of the ChCl/OA and ChCl/EG are -25.63 KJ/mol and -5.67 KJ/mol, respectively. This indicates that hydrogen bonding interaction in ChCl/OA is stronger than that in ChCl/EG.

Topological analysis of the two DES systems was shown in Supplementary Figure 25 and Supplementary Table 10, the key topological parameters (electron density (ρ) and Laplacian density ($\nabla^2\rho$)) at the bond critical points (BCPs) were calculated for ChCl/OA and ChCl/EG. Generally, ρ and $\nabla^2\rho$ were used to describe the properties of hydrogen bond.[5] As the results in Supplementary Table 10 showed, all the ρ and $\nabla^2\rho$ are within the range of electron density (0.002 to 0.035 a.u.) and Laplacian density ranges (0.024 to 0.139 a.u.), indicating the formation of hydrogen bonding interaction between the hydrogen bond acceptor and donor. In ChCl/OA and ChCl/EG DESs, Cl–H···O showed the strongest hydrogen bonding interaction with highest ρ and $\nabla^2\rho$ values of ρ=0.0476 a.u., $\nabla^2\rho$=0.0656 a.u. and ρ=0.0295 a.u., $\nabla^2\rho$=0.0699 a.u., respectively. It's worth to mention that the ρ of Cl–H···O in ChCl/OA is higher than ChCl/EG (0.0476 *versus* 0.0295), indicating a stronger hydrogen bonding interaction in ChCl/OA DES. The results were consistent with the interaction energy and bond length analysis.

4.2.2 Molecular dynamic simulation of DESs

The simulation of different DES systems[4] showed that in ChCl/EG DES, the average number of hydrogen bonds (HBs) between chloride and ethylene glycol (Cl-EG) is 258 (Supplementary Figures 26 and 27). When oxalic acid (OA) was added for preparing DP-DES10, it can be observed that the HBs of Cl-EG decreased to 241 and the HBs of Cl-OA are 17, this is because the hydrogen bonding interaction of Cl-OA is stronger than Cl-EG as suggested by DFT calculation.

Supplementary Note 5: DES for lignocellulose fractionation studies

We have now performed a series of control experiments using microcrystalline cellulose (MCC) and several model compounds (Supplementary Figure 49). In these experiments, MCC alone or MCC in the presence of xylan (as hemicellulose model) or lignin was treated under the reaction conditions resembling lignocellulose fractionation in either ChCl/OA DES (120°C for 30 min) or DP-DES10 (100°C for 24 h). When MCC or a combination of MCC and xylan (0.45g MCC and 0.35g xylan) were fractionated in ChCl/OA DES, no nanosized particles were observed by SEM on the recovered cellulose residues (white color). However, when the MCC+xylan+lignin (0.45g MCC, 0.35g xylan and 0.2g lignin) combination was treated in ChCl/OA DES, obvious nanosized particles were formed on the surface of the residues (dark brown). When the same experiments were performed in DP-DES10, the cellulose residue (light brown and fine surface) showed a relatively "clean" surface without any particles, the cellulose appeared fibrillated into nanofibers. These results were also consistent with SEM analysis of the cellulose residues from the corresponding lignocellulose fractionation experiments (Supplementary Figure 51). These experiments strongly suggest that the nanosized particles are condensed lignin. The ability of DP-DES10 to 'protect' the lignin from condensation is likely responsible for this behavior, compared to ChCl/OA DES which has higher acid content and no stabilization function.

5.1 Thermal behaviour and characterization of the obtained lignins

5.1.1 Thermal degradability

The thermal stability of two lignin samples (**DPL10** and **ChCl/OAL**) was determined by TG-FTIR, respectively (Supplementary Figures 45 and 46) The yield of solid residues for **DPL10** and **ChCl/OAL** after 800°C degradation was 24.5% and 40.8% respectively,

consistent with the degree of condensation. The results of TG-FTIR at 220°C also showed differences between the two samples. Both samples displayed typical peaks of water with the characteristic bands at 3500-3964 cm^{-1} and 1300-1800 cm^{-1} as well as characteristic bands at 2271-2391 cm^{-1} and 586-726 cm^{-1} indicative of CO_2 formation.[40] These signals were more pronounced for **DPL10** and less intense for **ChCl/OAL**, as expected from the respective β-O-4 contents and level of condensation.

5.2.2 Pyrolysis GC-MS

In addition, these lignins **ChCl/OAL** and **DPL10** are further characterized by pyrolysis GC-MS (Supplementary Table 14 and Figure 47). The stabilized **DPL10** lignin clearly demonstrated higher yield of pyrolytic monomers compared to **ChCl/OAL**, consistent with TG-FTIR analysis.

5.2 Control experiments related to SEM imaging of condensed lignin.

5.2.1 SEM images of untreated wood, the CRs and model components studies at high and low magnification

Untreated lignocellulose was characterized by SEM as control (Supplementary Figure 50). ChCl/OA CR and DPCR10 were characterized by SEM at high magnification, see Figure 6c-f in the main text or Supplementary Figure 51. The SEM images of ChCl/OA CR at low magnification (Supplementary Figure 51a) and high magnification (Supplementary Figure 51b), show obvious nanoparticles formation on the surface. In comparison, the cell structure of DPCR10 at low magnification (Supplementary Figure 51c) and high magnification (Supplementary Figure 51d), was fibrillated and without formation of nanoparticles.

5.3 In depth sugar analysis

5.3.1 Analysis of the behavior of xylan and MCC in DES

Microcrystalline cellulose (MCC) alone or xylan were separately treated in different DES at 100 °C for 24 h (Supplementary Figures 52a and 52b). Gratifyingly, MCC was very stable in **DP-DES10** (99% retention) and displayed high stability in **DP-DES20** (containing more OA) as well (93.5% retention). This is in good agreement with the lignocellulose fractionation data see Supplementary Tables 16 and 18.

Next, 0.35g xylan was stirred in **DP-DES10** at 100°C for 24h as hemicellulose model (Supplementary Figure 49c). A clear liquid was obtained after the indicated time, without any coloring, indicating full dissolution of xylan. In order to determine whether xylan has further reacted, the mixture was diluted with 10 mL water, filtered through a 0.22 μm microfilter and further analyzed by HPLC in terms of xylose content. Partial hydrolysis of xylan was confirmed by detecting 7.2±0.42% yield of xylose.

5.3.2 In–depth composition analysis of the obtained CR

The hemicellulose retention and composition residues after different DES treatments were analyzed with HPAEC-PAD. The obtained residues were hydrolyzed via acid hydrolysis (samples were diluted 20 times before injection). Therefore, about 15 mg of each pellet was mixed with 0.45 mL 72% (w/w) sulfuric acid and incubated for 1h at 30°C. After diluting the acid to 1 M, the incubation was continued for 3h at 100°C to fully hydrolyze the carbohydrates into monomers. Arabinose, galactose, glucuronic acid, mannose, rhamnose, and xylose were quantified by integrating the peak area of corresponding standards. Total hemicellulose content was calculated as a sum of all neutral sugars and glucuronic acid.

Elution of monosaccharides (0.25 mL min^{-1}) was performed with a multi-step-gradient using the following eluents: A: 0.1 M NaOH, B: 1 M NaOAc in 0.1 M NaOH, C: 0.2 M NaOH, and D: milliQ water. All analyzed monosaccharides elute in the first 20 min with 16% A, 84% D. Followed by 5 min with 45% A, 5% B, 50% D and 15 min with 60% A, 40% B. To regenerate the column it was flushed 12 min with 100% C by increasing flowrate in first 2 min to 0.35 mL min-1. Finally, the column is equilibrated for 12 min with 16% A, 84% D by decreasing the flow rate in the first 2 min to 0.25 ml min^{-1}.

The obtained hemicellulose composition of Birchwood was in a similar range of previously published data[41,42]. It contains xylose (25% w/w) in the polymeric form of xylan.

Around 2% w/w of galactose was detectable and traces of arabinose and rhamnose. The applied DES treatments lead to a loss in total hemicellulose content. However, the ternary DES treatment retain more hemicellulose (~38% w/w) if compared to the binary DES treatment (23% w/w). The total hemicellulose content decreased from 28% w/w in raw birchwood down to 12% w/w after ChCl/OA DES treatment. It was shown that xylan was debranched completely and partially degraded as can be seen in the loss in xylan content and complete loss of galactose, rhamnose and arabinose.

Supplementary Note 6: DES recycling

6.1 DES recycling

6.1.1 Quantification of losses of ChCl and EG during recycling by ^{1}H NMR spectroscopy

Quantitative analysis of the components in DP-DES10 (original) and RDP-DES10 (after recycling) was performed by ^{1}H NMR spectroscopy using 29.7 mg guaiacol as external standard as shown in Supplementary Figure 33, semi-quantification was performed using the $–CH_2$- protons for EG and $–CH_3$ protons for ChCl versus $–CH_3$ protons of guaiacol. The relative quantities of choline chloride to guaiacol in DP-DES10 and RDP-DES10 were $1.19x10^{-4}$ and $1.18x10^{-4}$, showing minimal loss of choline chloride after recycling. The **EG** amount decreased from $5.32x10^{-5}$ in DP-DES10 to $3.66x10^{-5}$ in RDP-DES10 after recycle, indicating an approximately 30% loss of EG during the fractionation process.

6.1.2 Quantification of losses of Oxalic acid (OA) during recycling

In order to quantify potential losses of oxalic acid (OA) during recycling and DES treatment, the pH values of the fresh DP-DES10 and the recycled RDP-DES10 were determined according to the literature.[55] The pH value of the fresh DP-DES10 is 1.06, which changed to 2.57 after one reaction/recycling step (Entry 2). The pH value could be easily recovered by adding 107 mg OA (Entry 3) to RDP-DES10. When this DES was used for a new fractionation run and recycled again, the pH value appeared to be similar to that obtained previously 2.51 (Entry 4), showing good reproducibility of the pH change after processing.

A control recycled DES just after mixing DP-DES10 and lignocellulose without heating showed a less significant pH change to 1.59 (Entry 5), this indicates that the loss of OA may occur both in the fractionation and recycling procedure.

As comparison, the other DES compositions with more acid content, such as the ChCl/OA as well as the CS-DES designed for acidolysis or DP-DES20, had pH values of 0.62, 0.74, 0.9 respectively, as expected.

6.1.3 Investigation of purity of the recycled DES and its effectiveness for further fractionation

In order to determine the purity of the DES after lignocellulose fractionation and recycling, we have next performed semi-quantitative ^{1}H NMR analysis of the used and recycled DES. First, we could assign all signals in the ^{1}H NMR spectrum of the fresh DP-DES10, which

beside the individual components, also showed small peaks of esters from the DES components, as already described in literature[6,7]. Semi-quantitative analysis was used to determine the “estimated purity” of DES according to the ^{1}H NMR of DES (this does not take into account changes in inorganics content). All signals were assigned, integrated and set the total integration to 100. The “estimated purity” of DES was calculated as follows: proton of all the unknown impurities/total protons in recycled DES×100%. It was seen, that the estimated NMR purity of freshly prepared DP-DES10 is 100%. The purity of the DESs was shown in Supplementary Table 12. When the composition of the DES obtained after recycling (with decreased EG and OA content) was re-adjusted, a new fractionation round resulted in excellent performance: good lignin yield and high β-O-4 content obtained, see Supplementary Figure 37.

Supplementary Note 7: Characterization of CRs by XRD and BET

The crystallinities of the untreated wood and fractionated cellulose residues (ChCl/OACR and DPCR10) were characterized by X-ray diffraction (XRD). All the samples showed typical cellulose I structure as shown in Supplementary Figure 56, the crystallinity of untreated wood was 57% according to Segal’s method, while the ChCl/OACR and DPCR10 showed increased crystallinities of 68% and 70%, respectively. The increase in crystallinity is typical for DES fractionated samples, and is attributed to the removal of lignin and some of the hemicellulose (amorphous components) during fractionation.

The BET surface area of the cellulose residues was tested by Micromeritics ASAP2020 gas adsorption analyzer. The results are summarized in Supplementary Table 21. After fractionation in DES, ChCl/OACR and DPCR10 showed increasing BET surface area of 1.68 m^2/g and 1.89 m^2/g, respectively compared to untreated wood, consistently with literature. Both the surface area and volume of pores of ChCl/OACR were slightly lower than DPCR10. This is further hint at the existence of lignin condensation in the cellulose matrix in the case of the control reaction.

Supplementary Figures

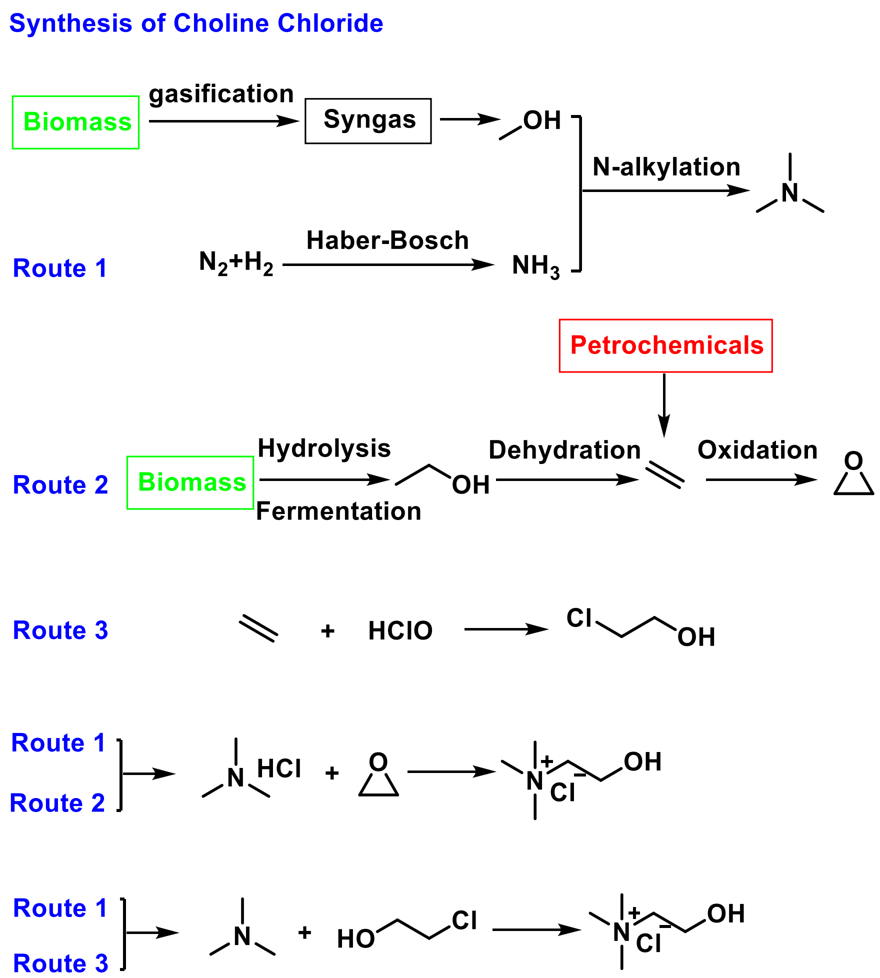

Supplementary Figure 1. Synthetic pathways for the preparation of choline chloride from petrochemicals versus renewable resources.[26–28]

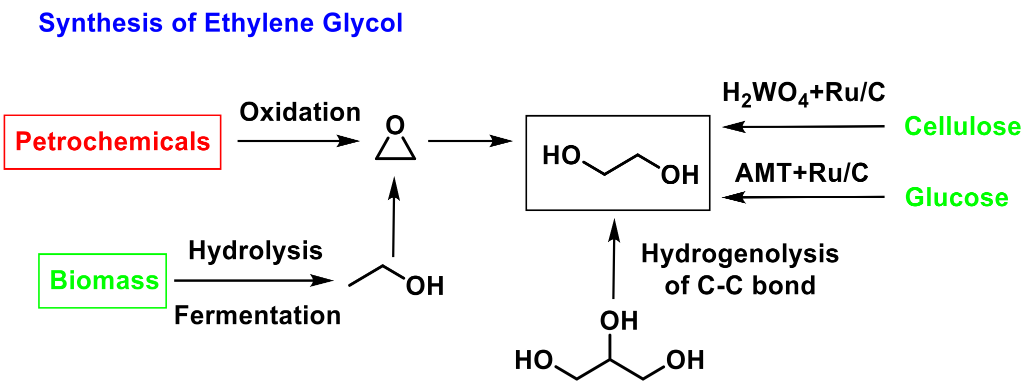

Supplementary Figure 2. Synthetic pathways for the preparation of EG from petrochemicals versus renewable resources.[22–24,29]

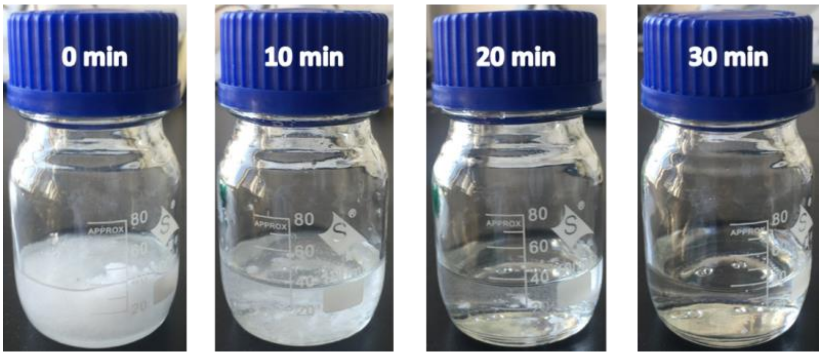

Supplementary Figure 3. Photograph of DP-DES10 preparation at room temperature within 30min.

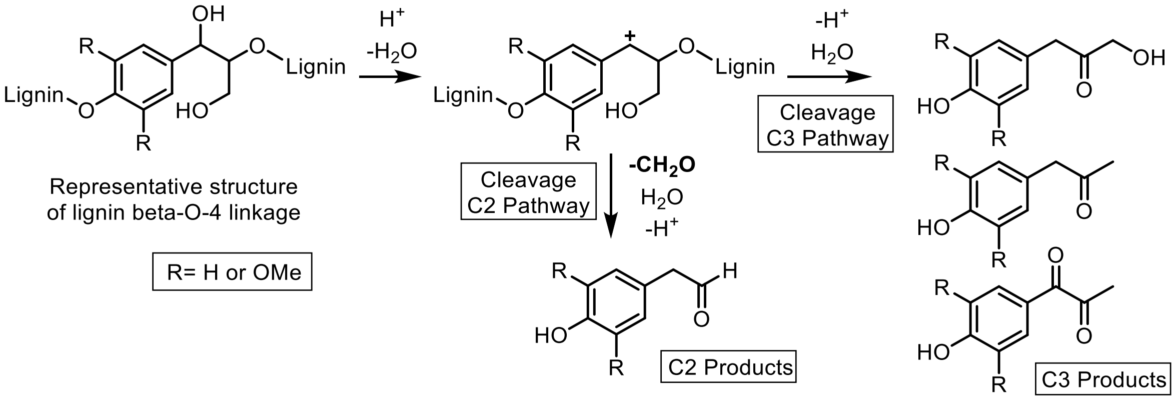

Supplementary Figure 4. Scheme of acid catalyzed lignin depolymerization pathways (C2 pathway and C3 pathway).[32]

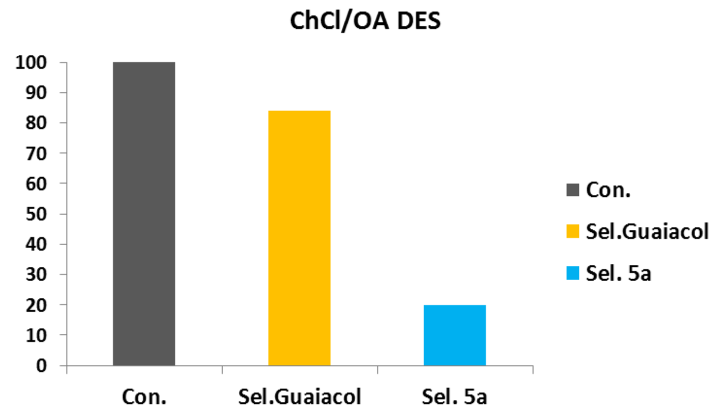

Supplementary Figure 5. 1a reaction in ChCl/OA DES at 100 °C for 2h, octadecane was used as an internal standard, conversion and selectivity were calculated by GC-FID. Full conversion of **1a** was achieved, the yield of guaiacol was 84% and the yield of aldehyde (**5a**) was 20%.

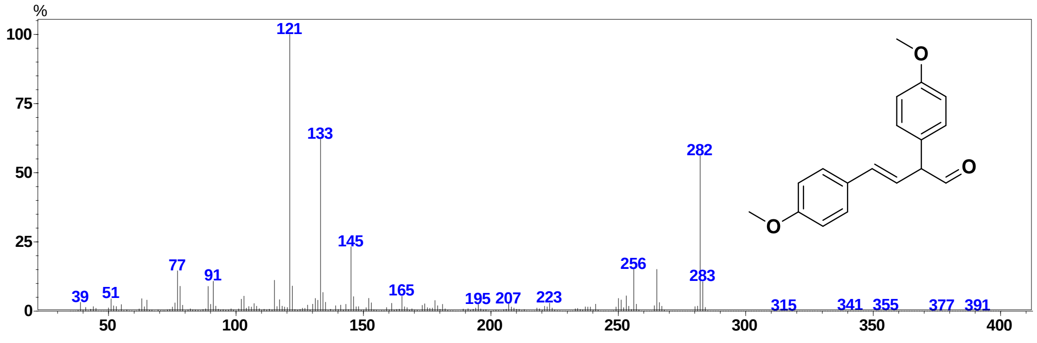

Supplementary Figure 6. Identification of the aldehyde condensation by-product by GC/MS in ChCl/OA DES. Showing a m/z of 282, the product was formed by aldol condensation of **5a**.

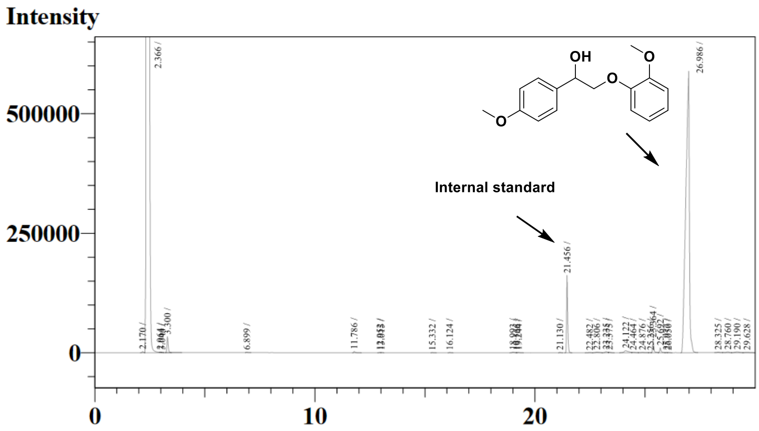

Supplementary Figure 7. GC-FID result of **1a** reaction in ChCl/EG DES, only **1a** starting material and octadecane as an internal standard were observed after reaction at 100 °C for 2 h.

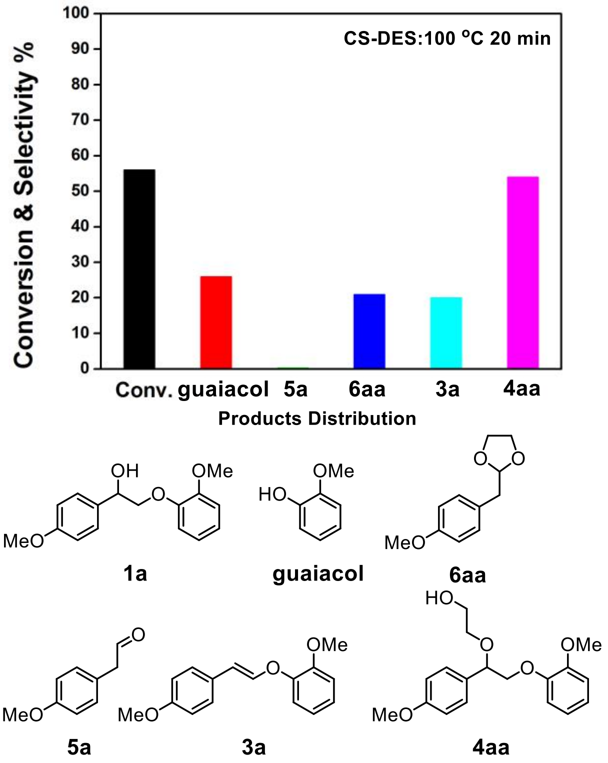

Supplementary Figure 8. Products distribution of **1a** in **CS-DES** (100 °C for 20 min) after reaction. **CS-DES** composition ChCl/OA/EG (1:1:2 molar ratio, 11.6 g), **1a** 25 mg, octadecane as an internal standard, conversion and selectivity were calculated by GC-FID, products identification was carried out generally by GC/MS.

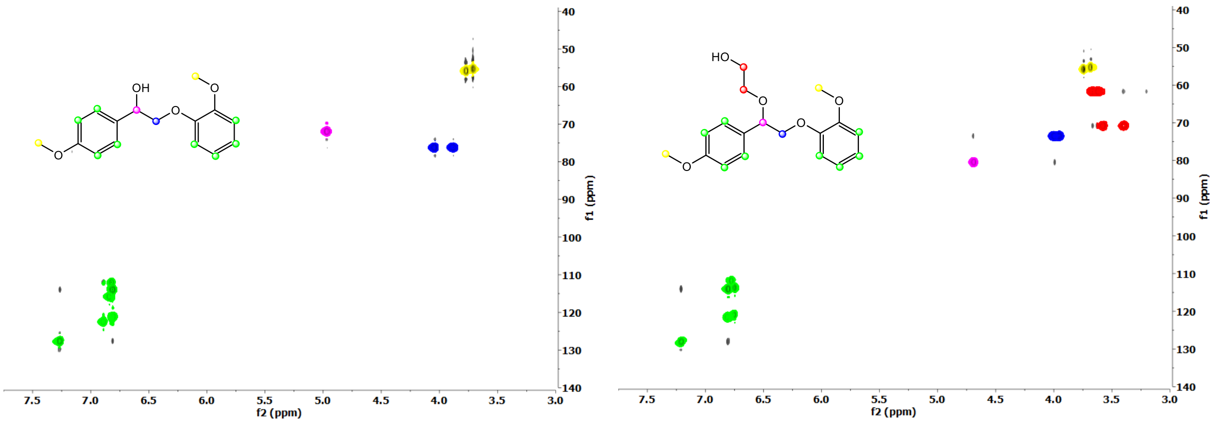

Supplementary Figure 9. 2D HSQC NMR spectrum of **1a** (left) and **4aa** (right), spectra were recorded in $CDCl_3$.

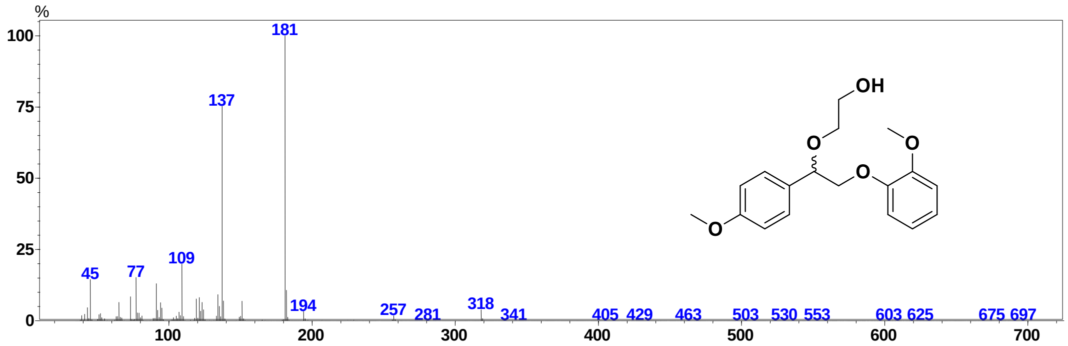

Supplementary Figure 10. Identification of **4aa** by GC/MS, showing a m/z of 318.

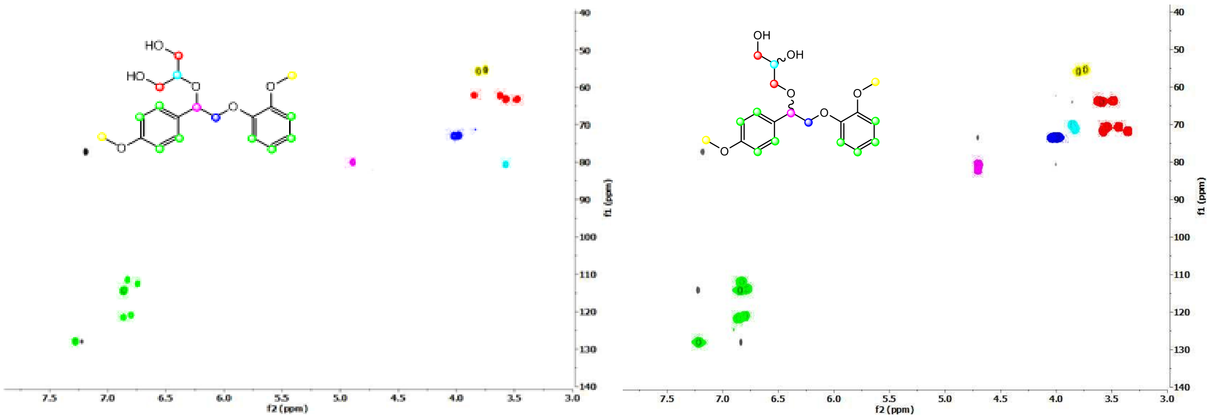

Supplementary Figure 11. 2D HSQC NMR spectrum of **4ab** (right) and **4ab´** (left), spectra was recorded in $CDCl_3$.

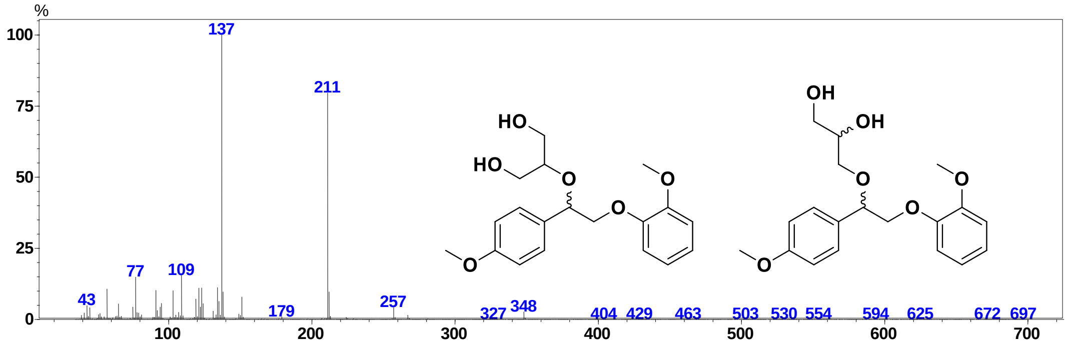

Supplementary Figure 12. Identification of **4ab** and **4ab´** by GC/MS, both of them show a m/z of 348.

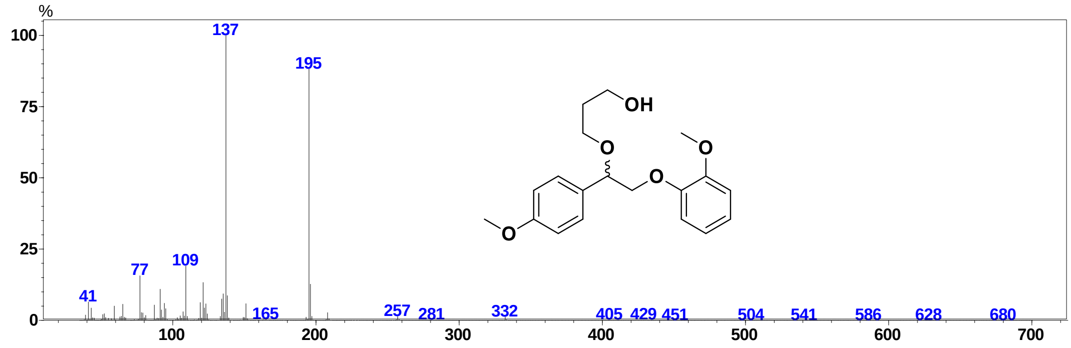

Supplementary Figure 13. Identification of **4ac** by GC/MS, showing a m/z of 332.

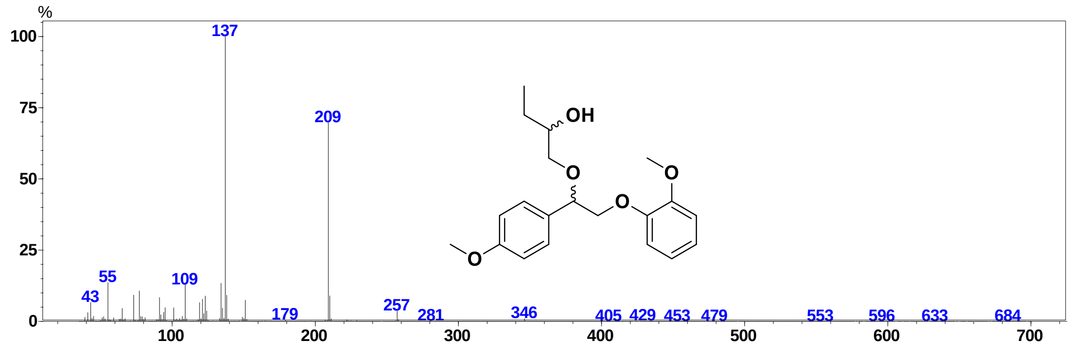

Supplementary Figure 14. Identification of **4ad** by GC/MS, showing a m/z of 346.

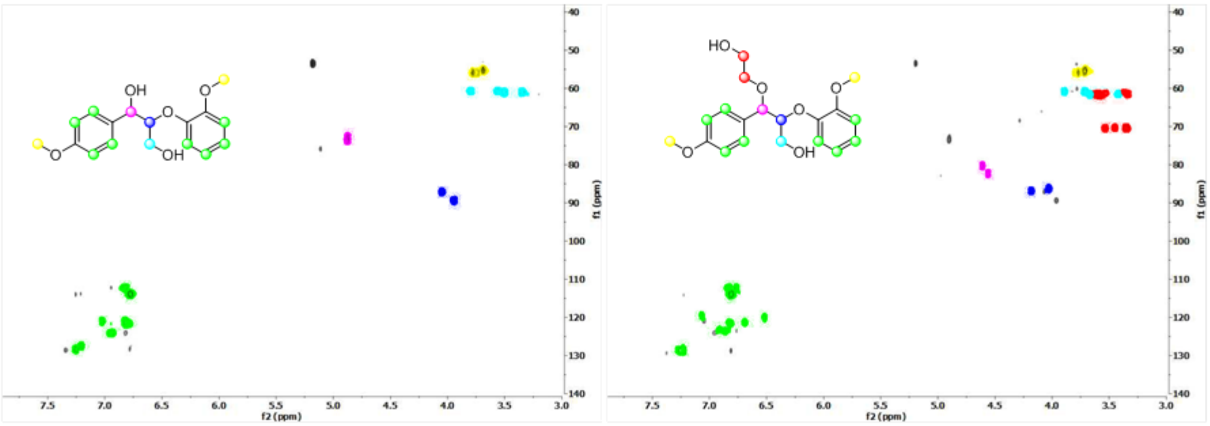

Supplementary Figure 15. 2D HSQC NMR spectrum of **1b** (left) and **4ab** (right), spectra was recorded in $CDCl_3$.

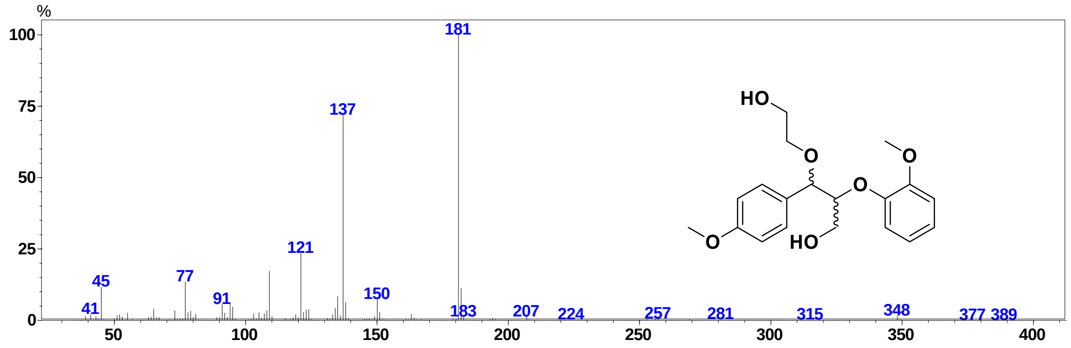

Supplementary Figure 16. Identification of **4ab** by GC/MS, showing a m/z of 348.

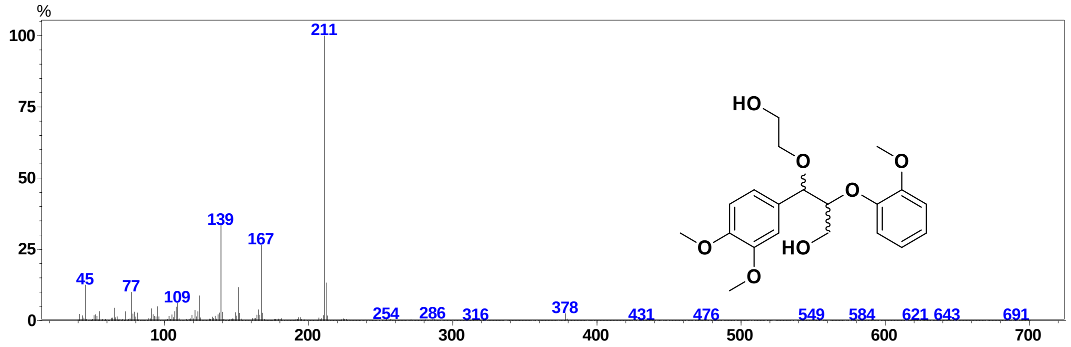

Supplementary Figure 17. Identification of **4ab** by GC/MS, showing a m/z of 378.

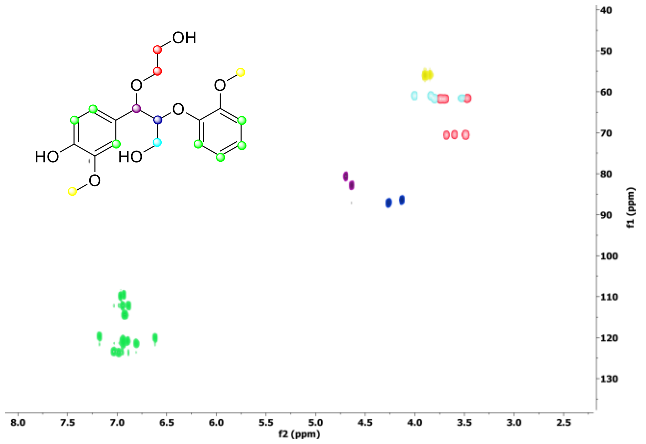

Supplementary Figure 18. 2D HSQC NMR spectrum **4da**, spectrum was recorded in $CDCl_3$.

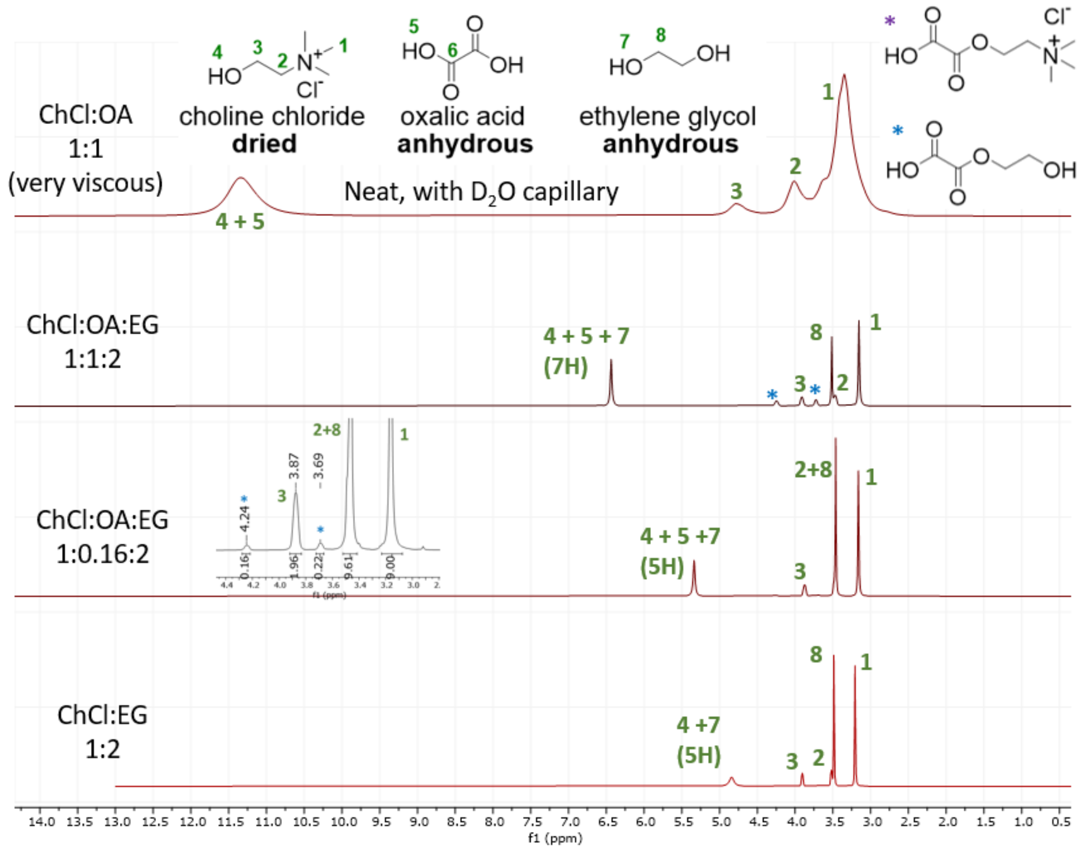

Supplementary Figure 19. ^{1}H NMR of neat DES (with D_2O capillary) prepared from anhydrous components

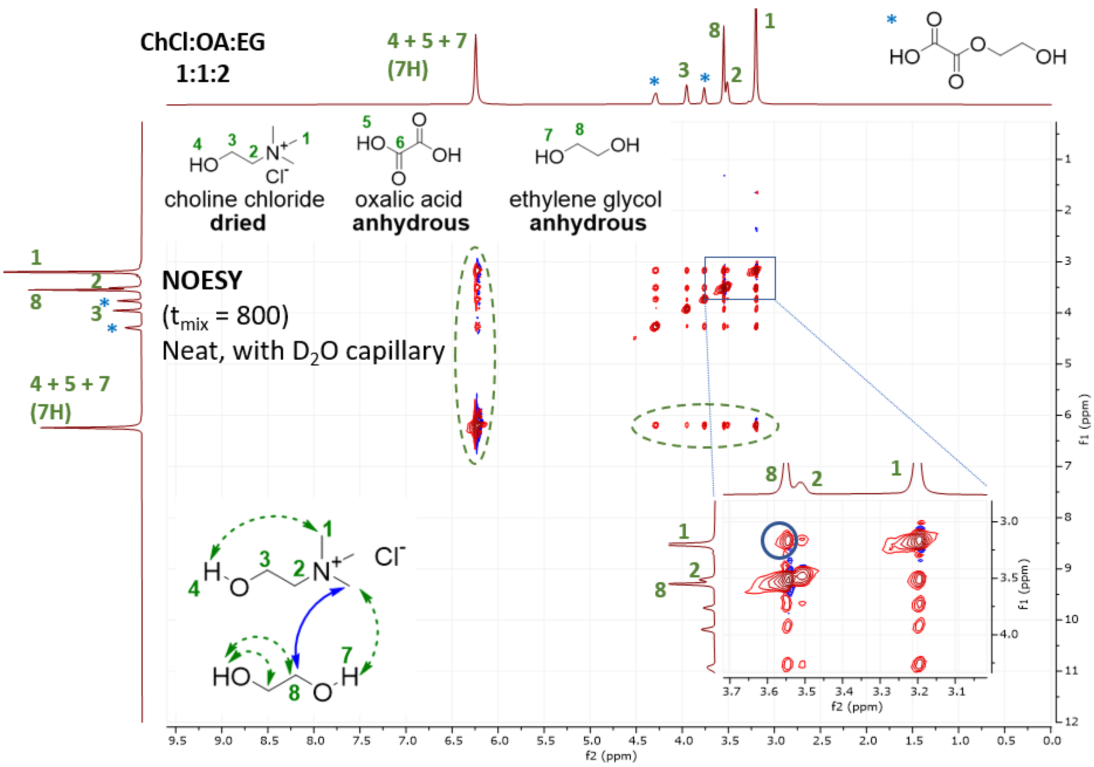

Supplementary Figure 20. NOESY spectrum of the ternary DES ChCl:OA:EG (1:1:2)

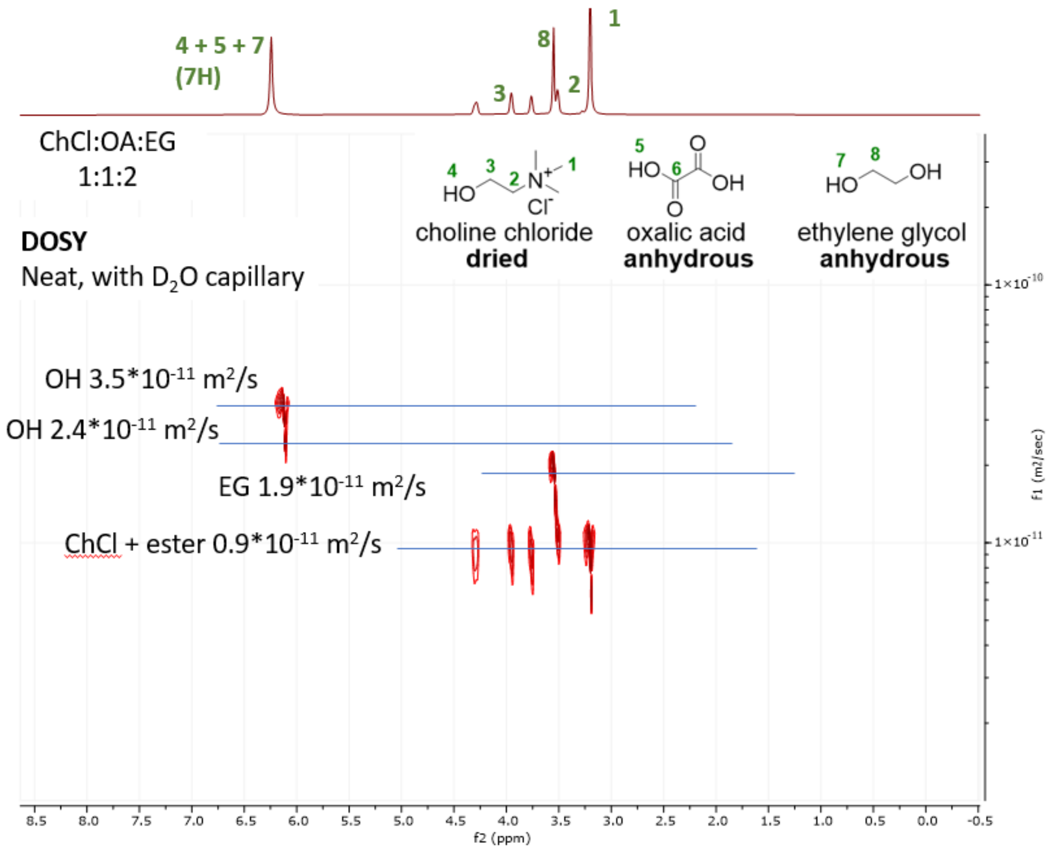

Supplementary Figure 21. DOSY spectrum of the ternary DES ChCl:OA:EG (1:1:2)

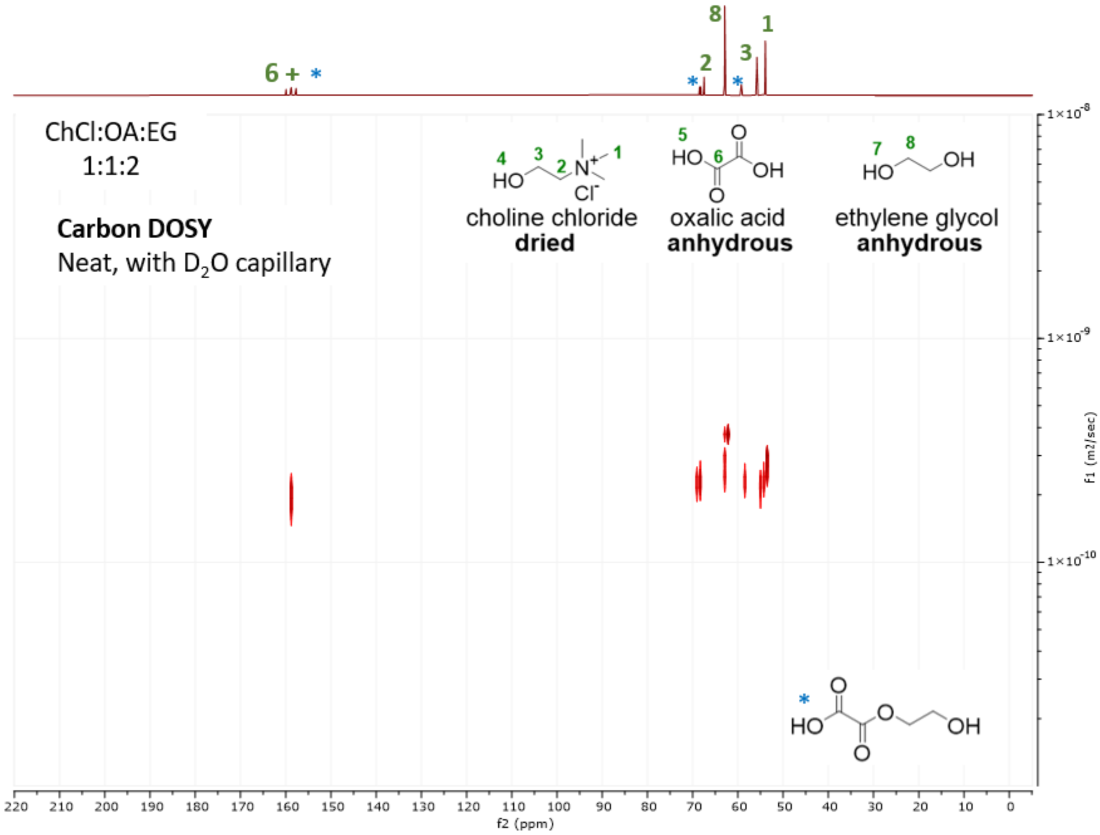

Supplementary Figure 22. Carbon DOSY of ChCl:OA:EG (1:1:2) DES (neat)

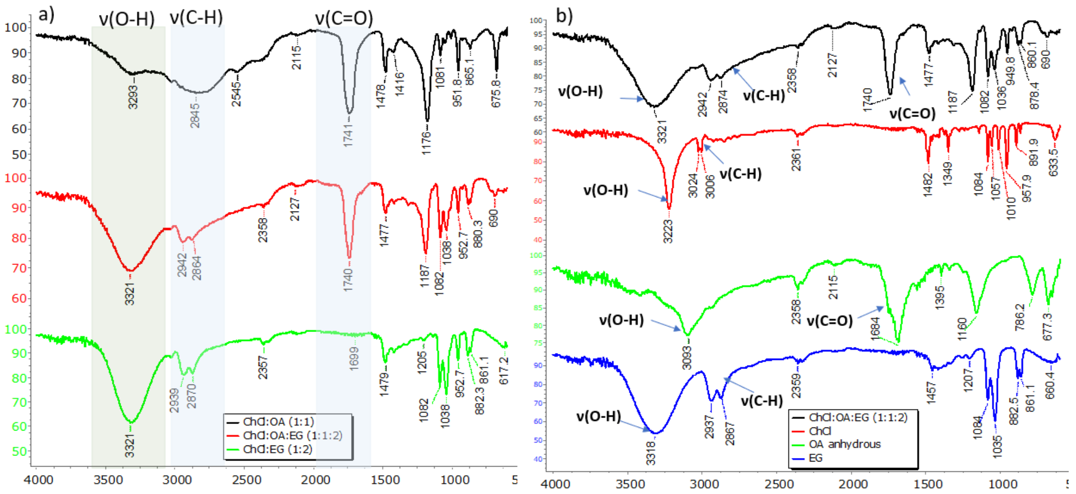

	ChCl:OA	ChCl:OA:EG	CHCl:EG	ChCl	OA	EG
v(O-H)*(stretching vibration), [cm^{-1}]*	3500-3050			3223	3300-2900	3500-3050
v(C-H) *(stretching vibration, CH_2, CH_3), [cm^{-1}]*	3000-2800	2942, 2864	2939, 2870	3024, 3006	-	2937, 2867
v(C=O) *(stretching vibration), [cm^{-1}]*	1741	1740	-	-	1684	-
v(C-O) *(stretching vibration), [cm^{-1}]*		1082, 1038	1082-1038	1084, 1057	1160	1084, 1035

Supplementary Figure 23. a) Comparison of the FTIR spectra of the DES (ChCl:OA - black, ChCl:EG - green and ChCl:OA:EG - red) b) Comparison of the FTIR spectra of DES ChCl:OA:EG (1:1:2) – black, choline chloride – red, oxalic acid – green, ethylene glycol – blue (anhydrous OA)

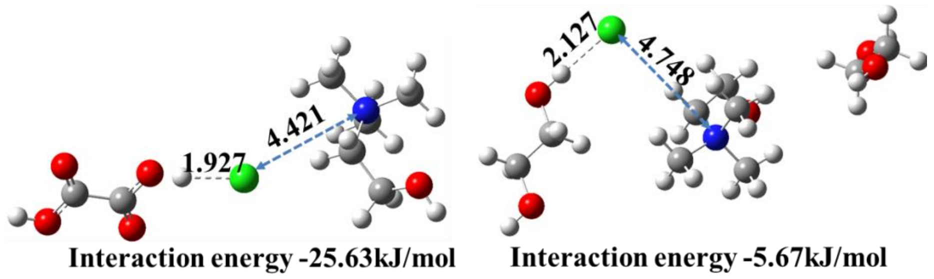

Supplementary Figure 24. Geometry of ChCl/OA, ChCl/EG at the B3LYP/6-311+G** level. Hydrogen: white; Carbon: Gray; Oxygen: Red; Chlorine: Green; Nitrogen: Blue.

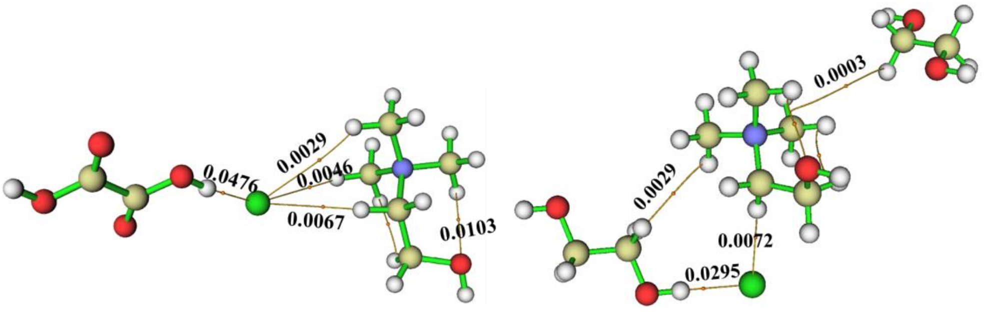

Supplementary Figure 25. Topological structures of ChCl/OA (left) and ChCl/EG (right) DESs.

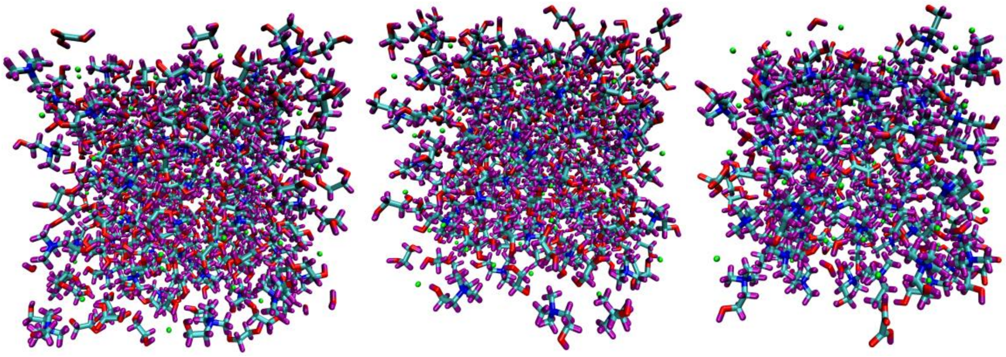

Supplementary Figure 26. Molecular dynamic simulation of different DES systems in the box.

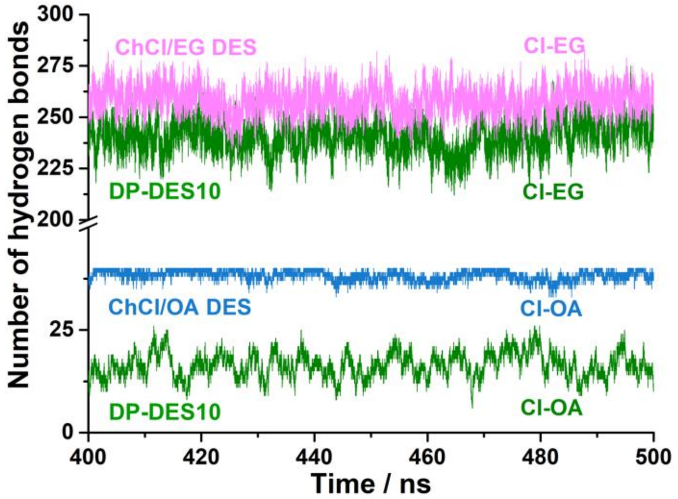

Supplementary Figure 27. Number of hydrogen bonds between chloride and hydrogen bond donors in different DES systems after simulation.

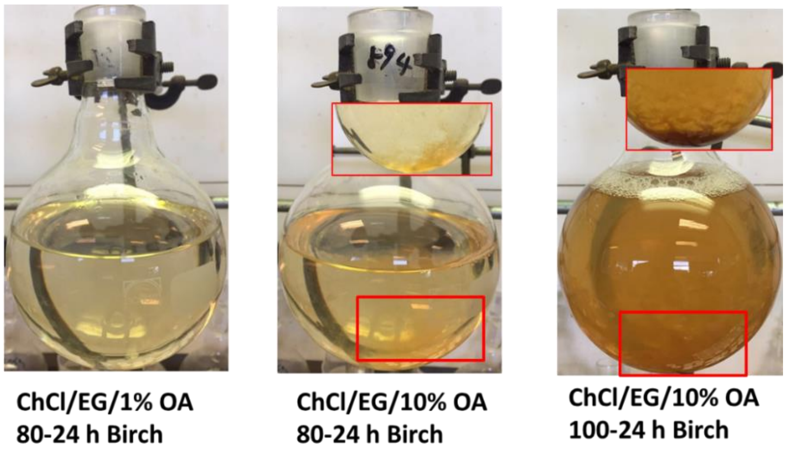

Supplementary Figure 28. Digital images of lignin precipitation in water, after fractionation of birch lignocellulose in various **DP-DES**.

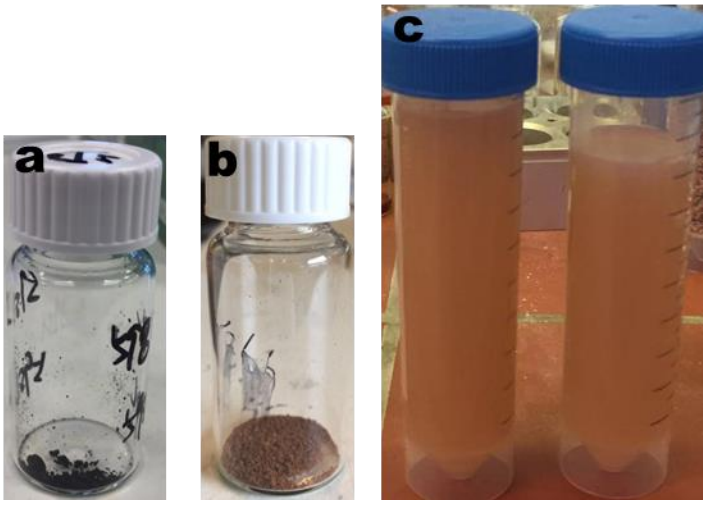

Supplementary Figure 29. Digital images of dried "black" lignin (a) isolated by traditional water precipitation method after filtration (15wt% yield) and brownish DPL10 (b) isolated by liquid/liquid extraction method;(c) stable lignin dispersion in water after centrifugation; **DP-DES10** was used for birch lignocellulose fractionation at 100 °C for 24 h.

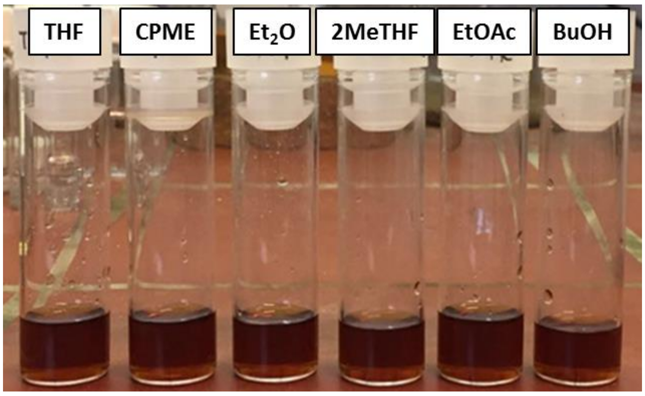

Supplementary Figure 30. Digital images of ~1 mL of **DP-DES10**/lignin mixture after removing the cellulose residue and ethanol.

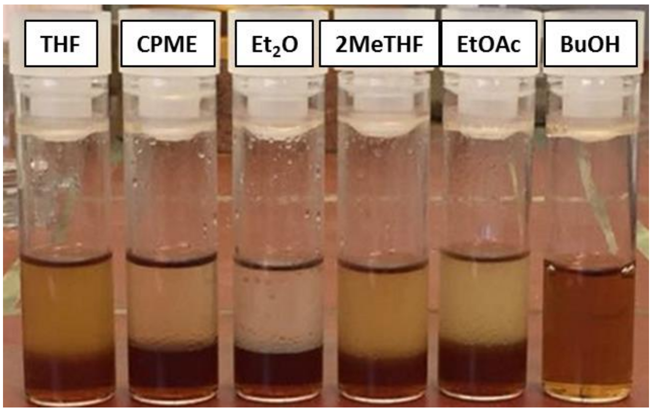

Supplementary Figure 31. Digital images of 2 mL of corresponding organic solvent added into ~1 mL of **DP-DES10**/lignin mixture after sonication.

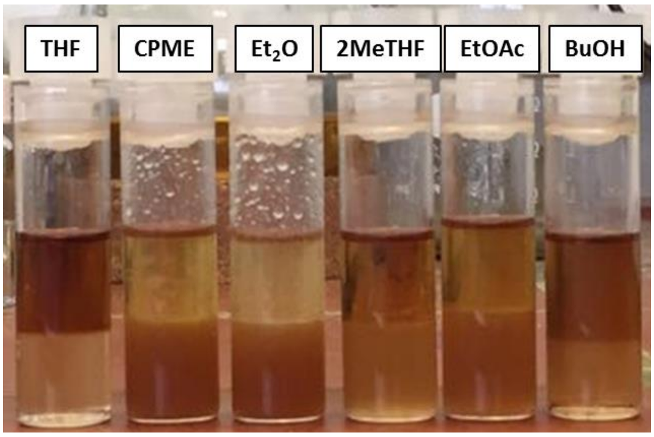

Supplementary Figure 32. Digital images of 2 mL of corresponding organic solvent and 1 mL water added into ~1 mL of **DP-DES**/lignin mixture after sonication. The combination of THF and water was promising for lignin extraction from **DP-DES**, brownish THF phase containing lignin was successfully extracted from **DP-DES**, the left aqueous **DP-DES** phase was nearly transparent.

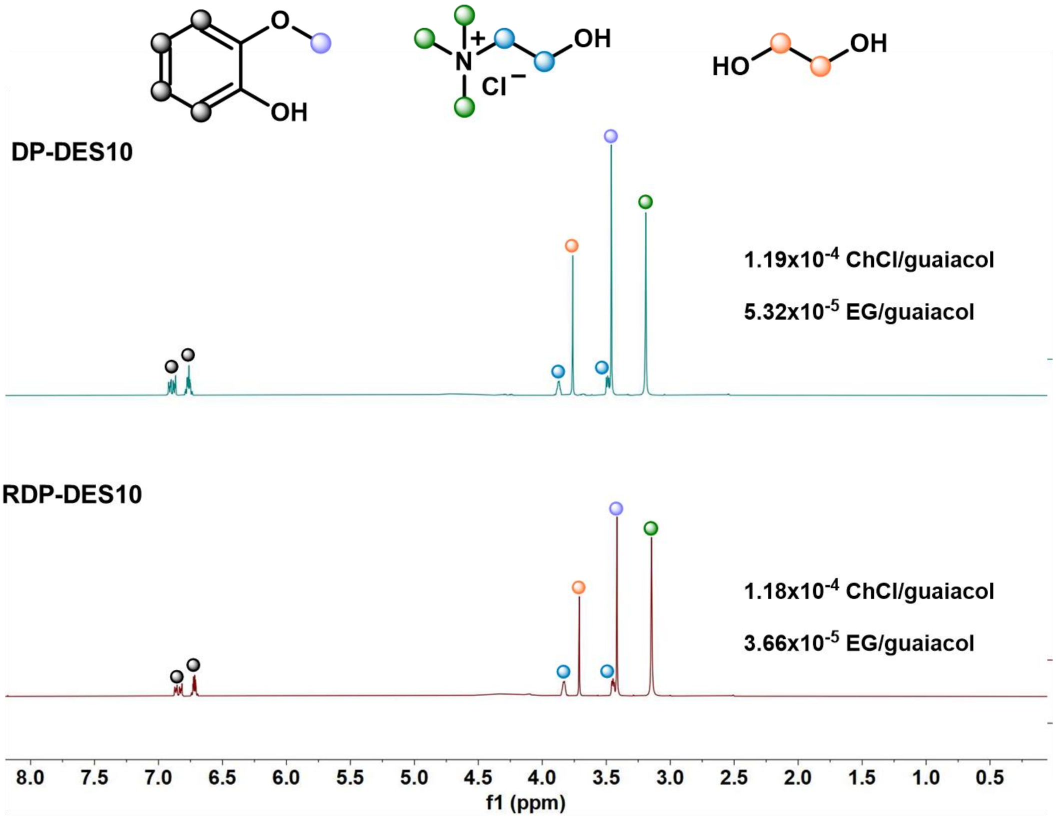

Supplementary Figure 33. Quantification of choline chloride and ethylene glycol in **DP-DES10** and **RDP-DES10** by ^{1}H NMR, using guaiacol as external standard; quantification was performed using the $–CH_2$- protons for EG and $–CH_3$ protons for ChCl versus $–CH_3$ protons of guaiacol. 29.7 mg guaiacol and 70.4 mg DP-DES10 or RDP-DES10 were dissolved in D_2O for NMR characterization.

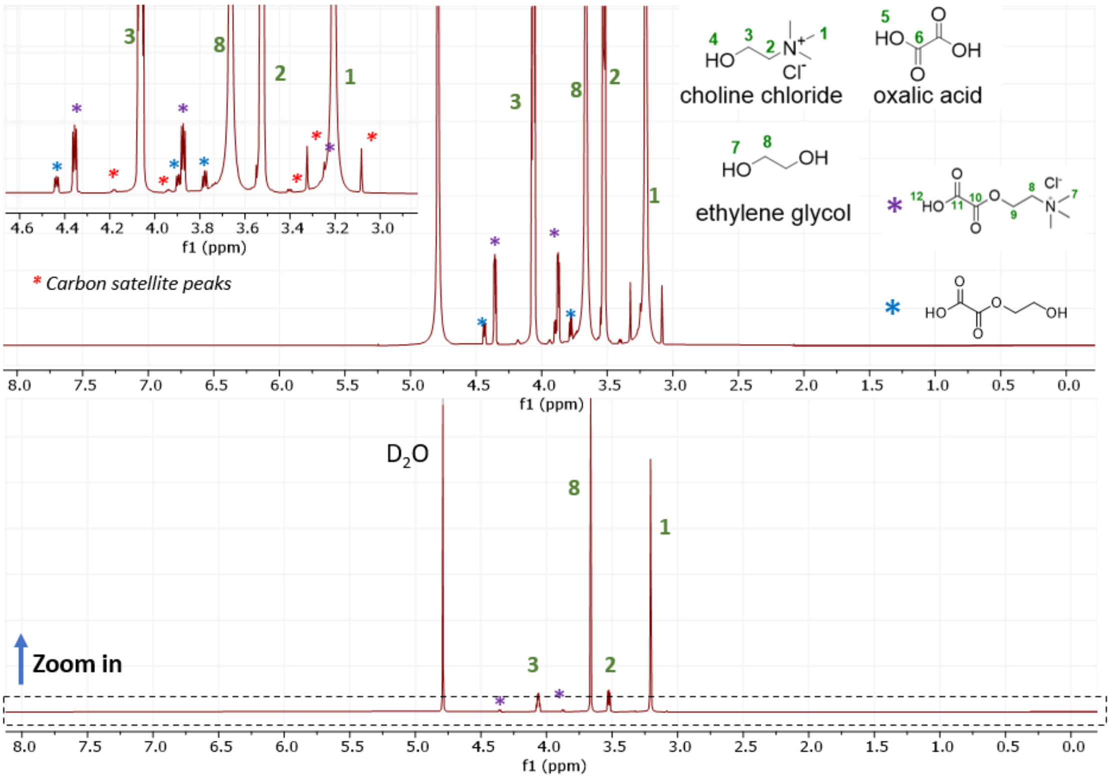

Supplementary Figure 34. ^{1}H NMR of fresh prepared DP-DES10 recorded in D_2O. "Estimated purity":100%.

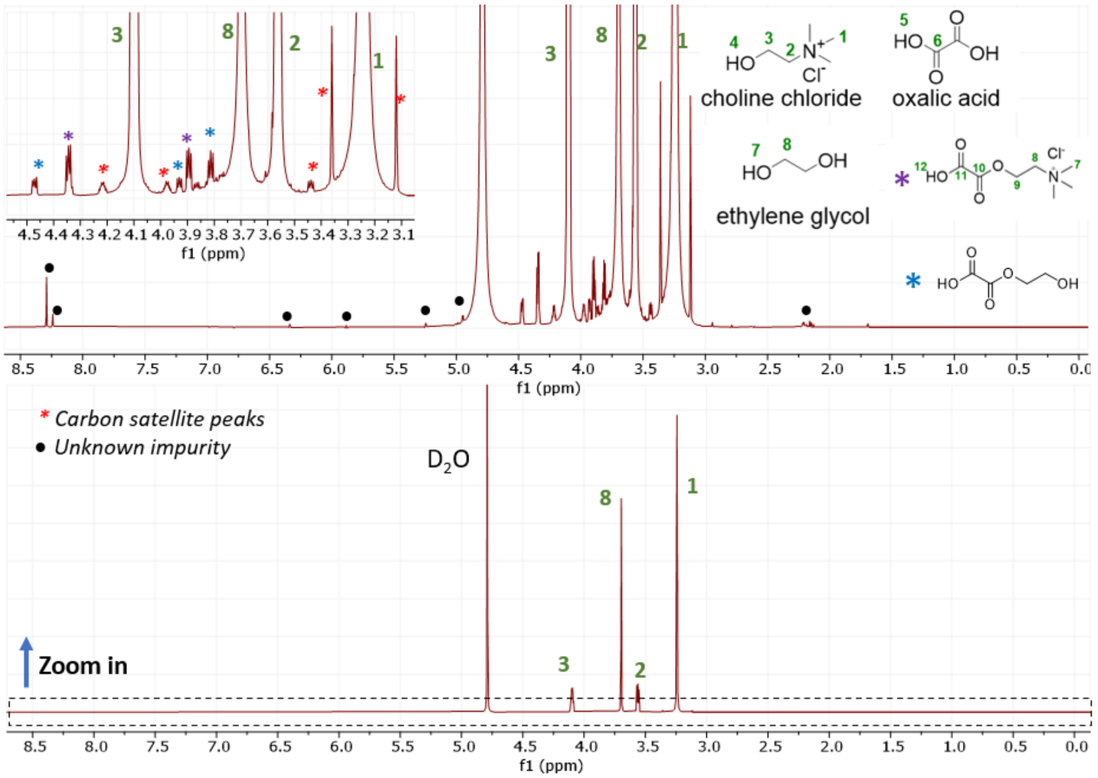

Supplementary Figure 35. ^{1}H NMR of RDP-DES10 recorded in D_2O. "Estimated purity": 99.8%.

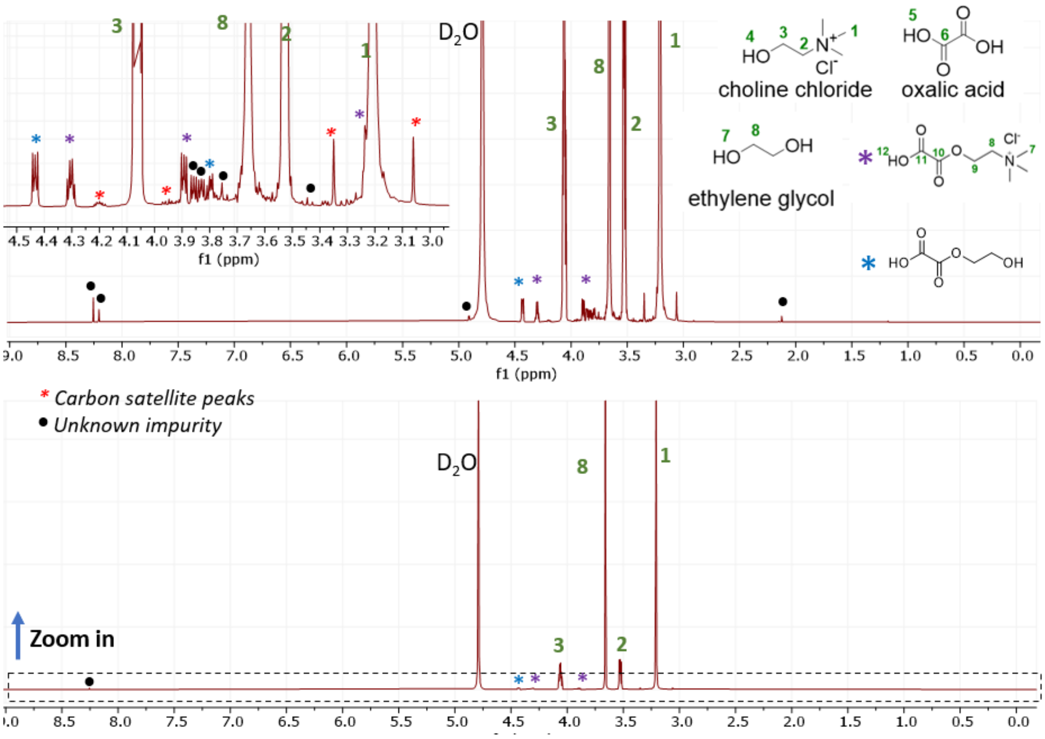

Supplementary Figure 36. ^{1}H NMR of recycled re-adjusted **RDP-DES10** recorded in D_2O. “Estimated purity”: 98.4%.

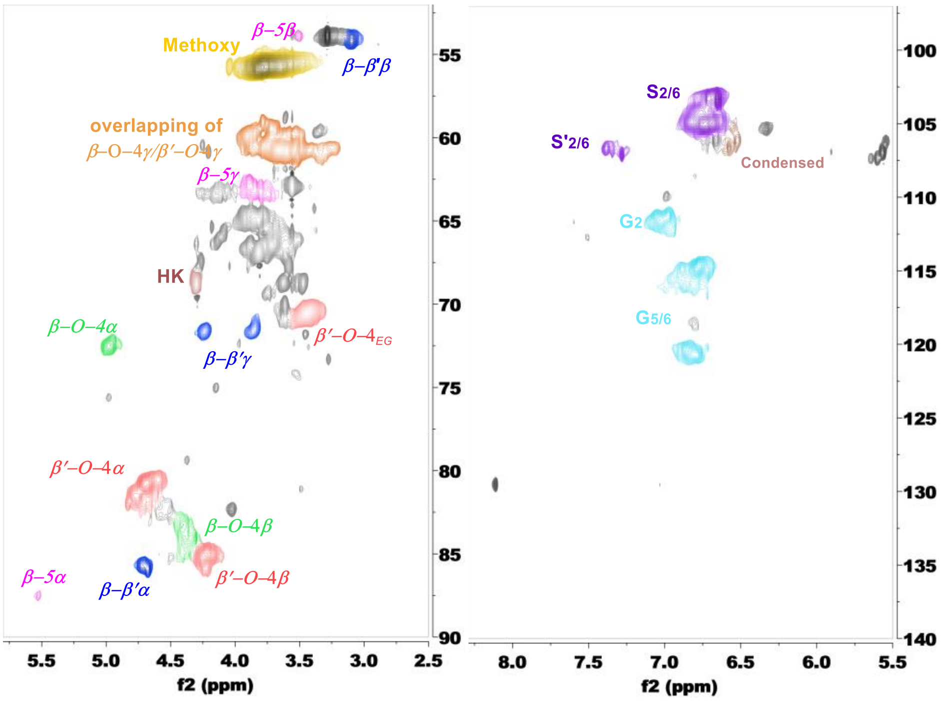

Supplementary Figure 37. 2D HSQC NMR of the lignin obtained in 42% yield after fractionation with re-adjusted RDP-DES10. From these NMR measurements the following parameters are concluded: S: 173.81; G: 70.5; $S_{condensed}$: 15.73; Total: 244.31; β-O-4: 8/100Ar; β′-O-4: 48/100Ar; β-β: 9/100Ar; β-5: 2/100Ar.

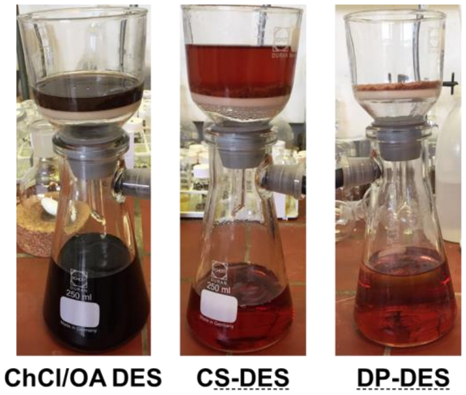

Supplementary Figure 38. Digital images of lignin filtrates after lignocellulose fractionation in ChCl/OA DES (black filtrates), **CS-DES** (brown filtrates) at 120 °C for 30 min and **DP-DES** at 100 °C for 24 h. ‘Black color’ represents severe condensation after fractionation.

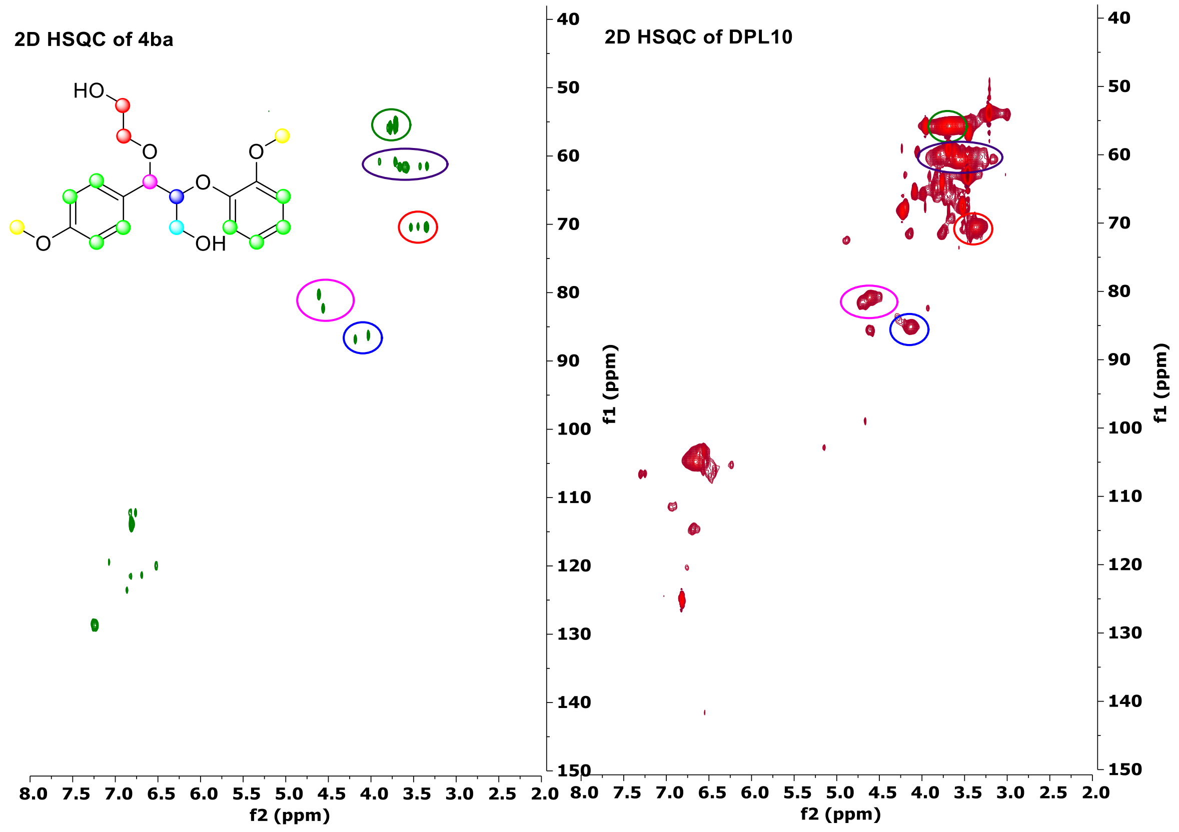

Supplementary Figure 39. ^{1}H-^{13}C HSQC NMR (600 MHz, acetone-d_6) of ethylene glycol incorporated lignin model compound **4ba** and ethylene glycol incorporated birch lignin **DPL10**.

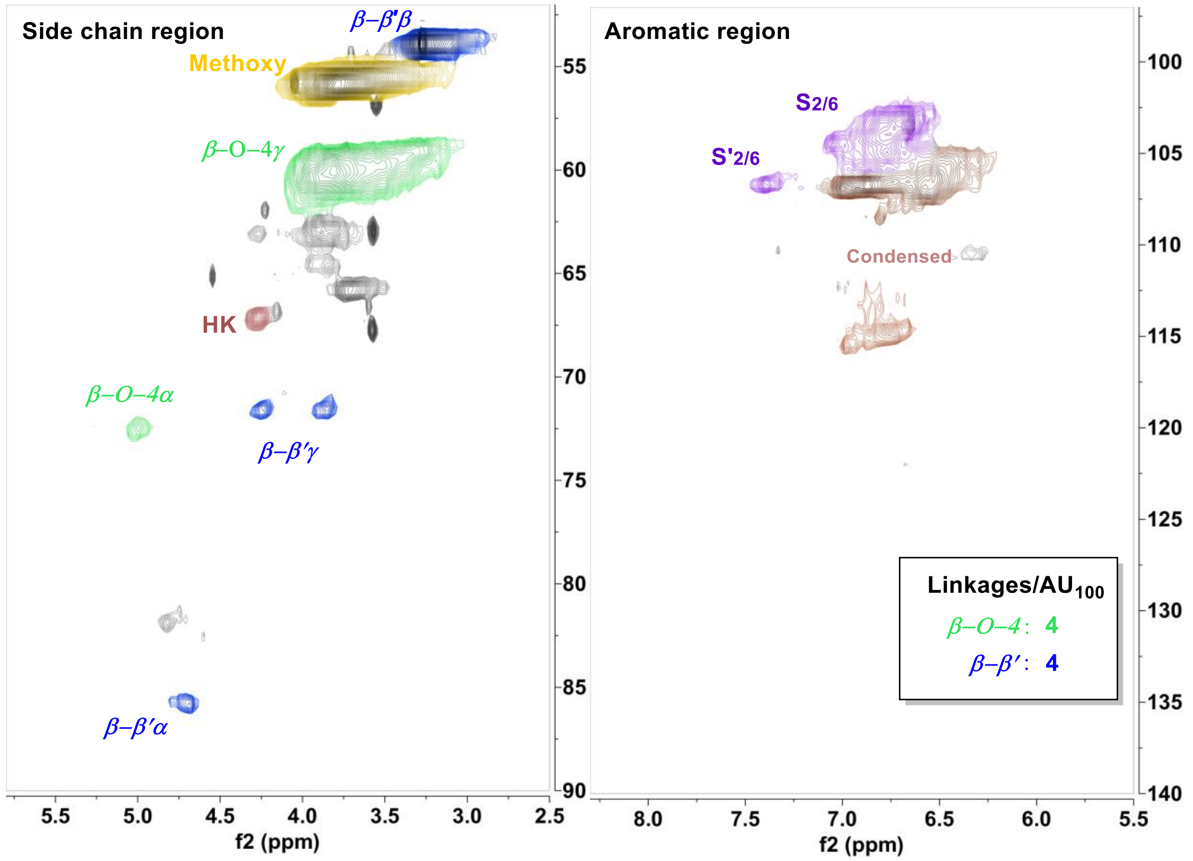

Supplementary Figure 40. ^{1}H-^{13}C HSQC NMR (600 MHz, acetone-d$_6$) of ChCl/OAL (binary DES: ChCl, 16.8 g, 120 mmol: OA, 120 mmol, 15.2 g), obvious condensation was observed and the β-O-4 content was low (4 per Au$_{100}$), $S_{condensed}$% = 53%.

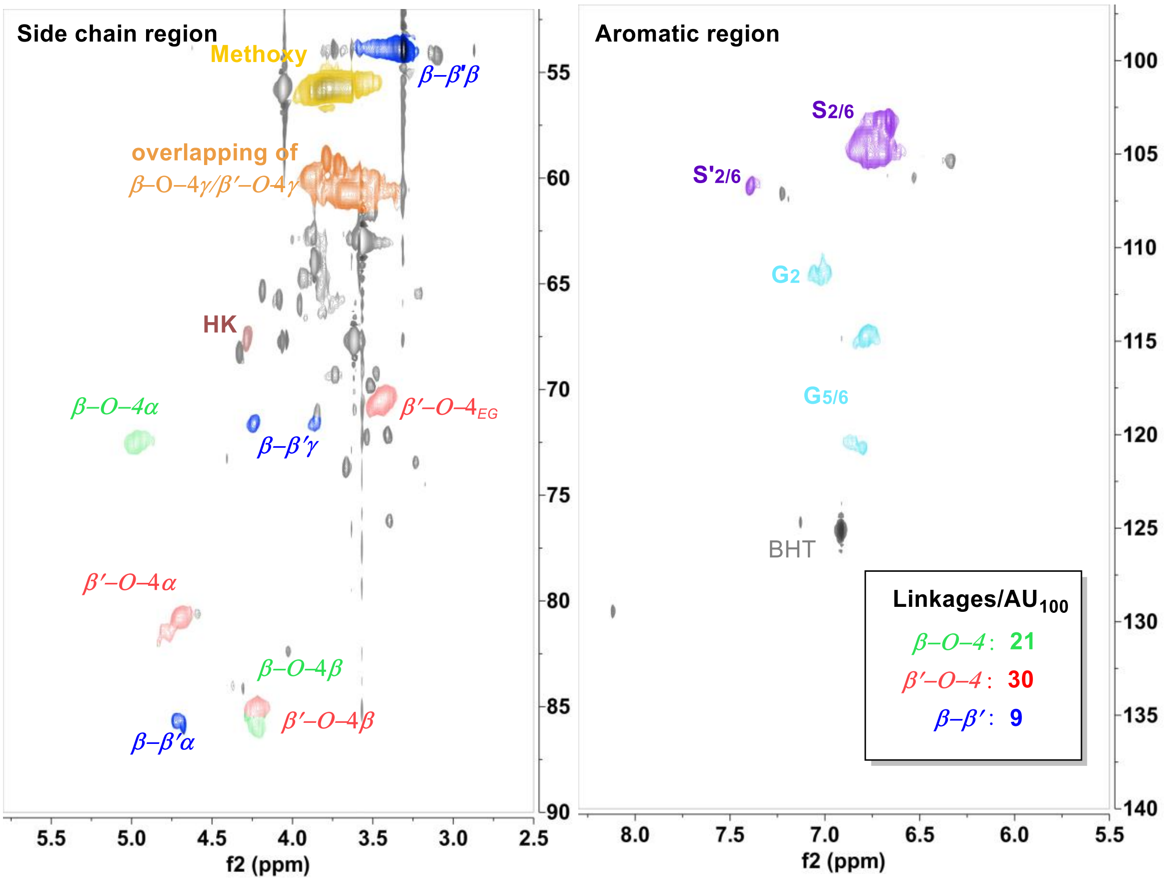

Supplementary Figure 41. ^{1}H-^{13}C HSQC NMR (600 MHz, acetone-d_6) of **RDP-DES10** (recycled DP-DES10) fractionated lignin. $S_{condensed}$% = 15%.

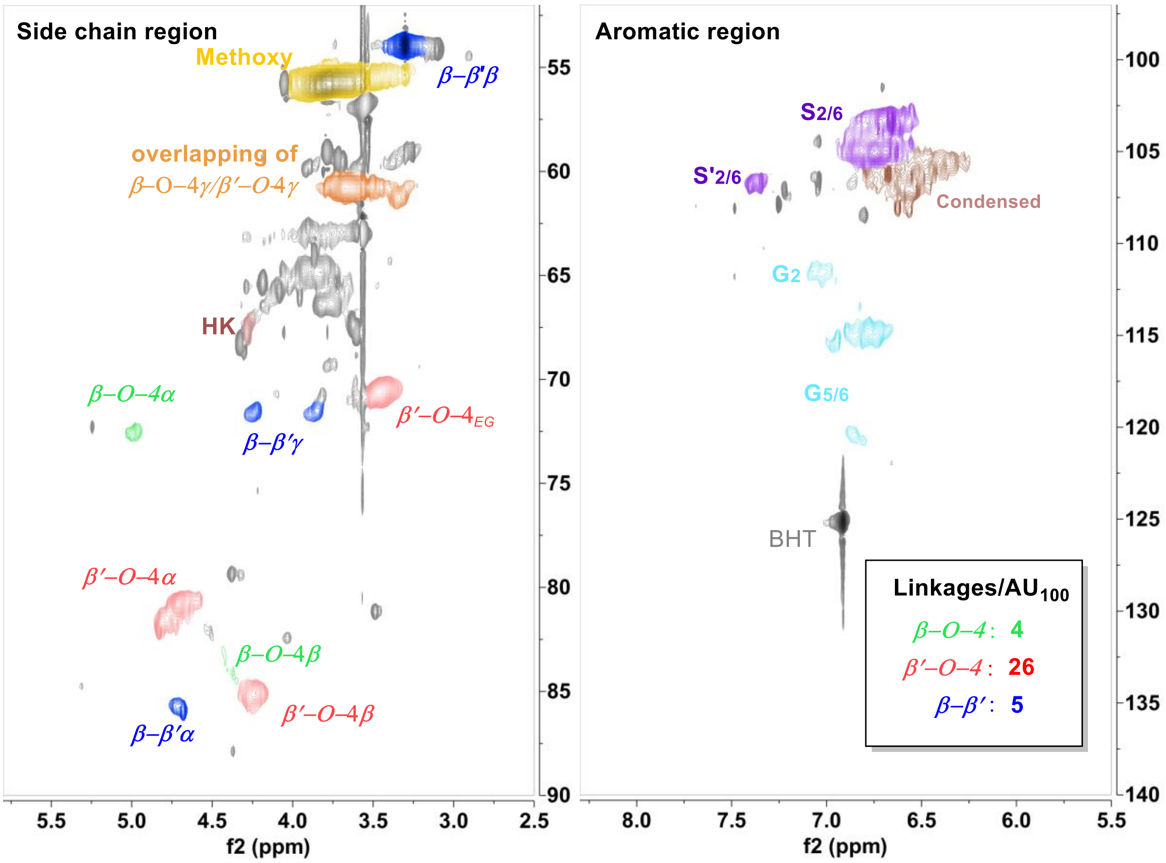

Supplementary Figure 42. ^{1}H-^{13}C HSQC NMR (600 MHz, acetone-d$_6$) of **DP-DES20** (ChCl, 16.8 g, 120 mmol: OA, 2.4 mmol, 6.4 g, 20 wt%: EG, 240 mol, 14.4 g) fractionated lignin at 80 °C for 24 h, $S_{condensed}\% = 27\%$.

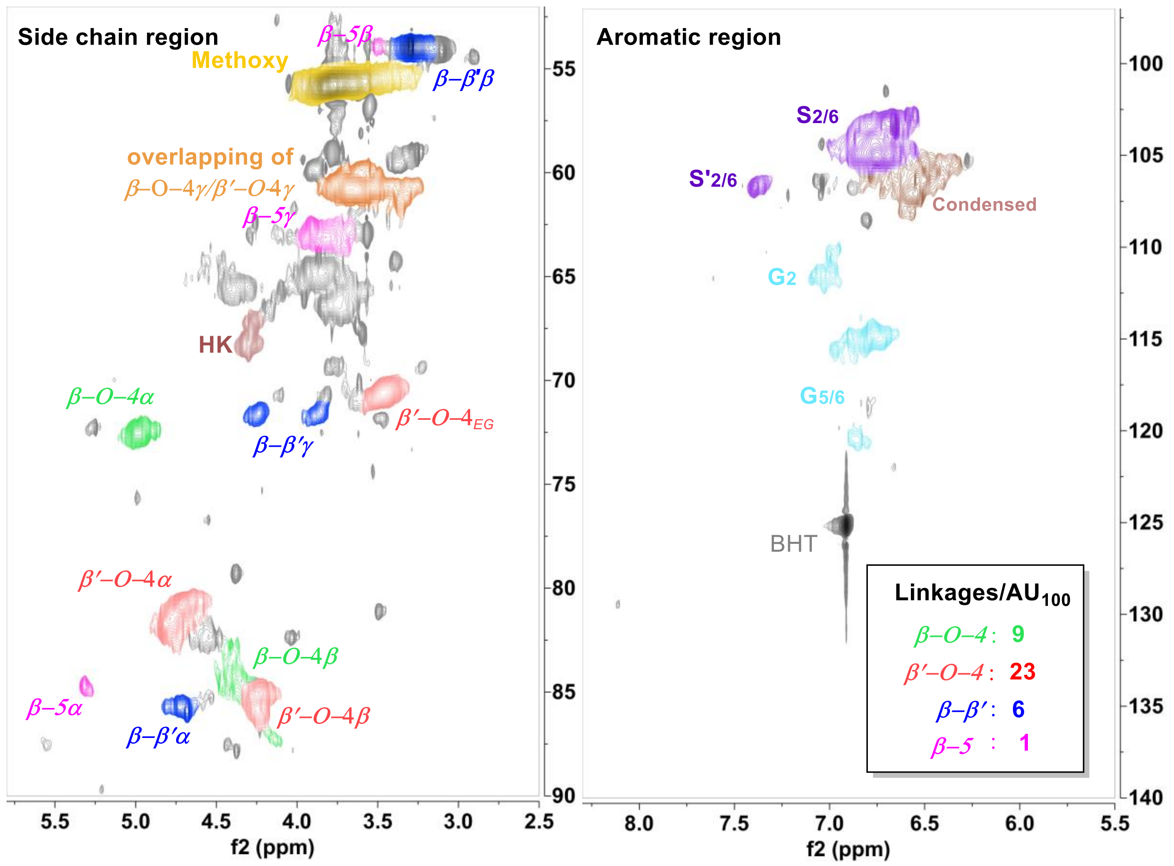

Supplementary Figure 43. ^{1}H-^{13}C HSQC NMR (600 MHz, acetone-d_6) of **CS-DES** (ChCl, 16.8 g, 120 mmol: OA, 120 mmol, 15.2 g: EG, 240 mmol, 14.4 g) fractionated lignin at 120 °C for 30 min, $S_{condensed}\%$ = 23%.

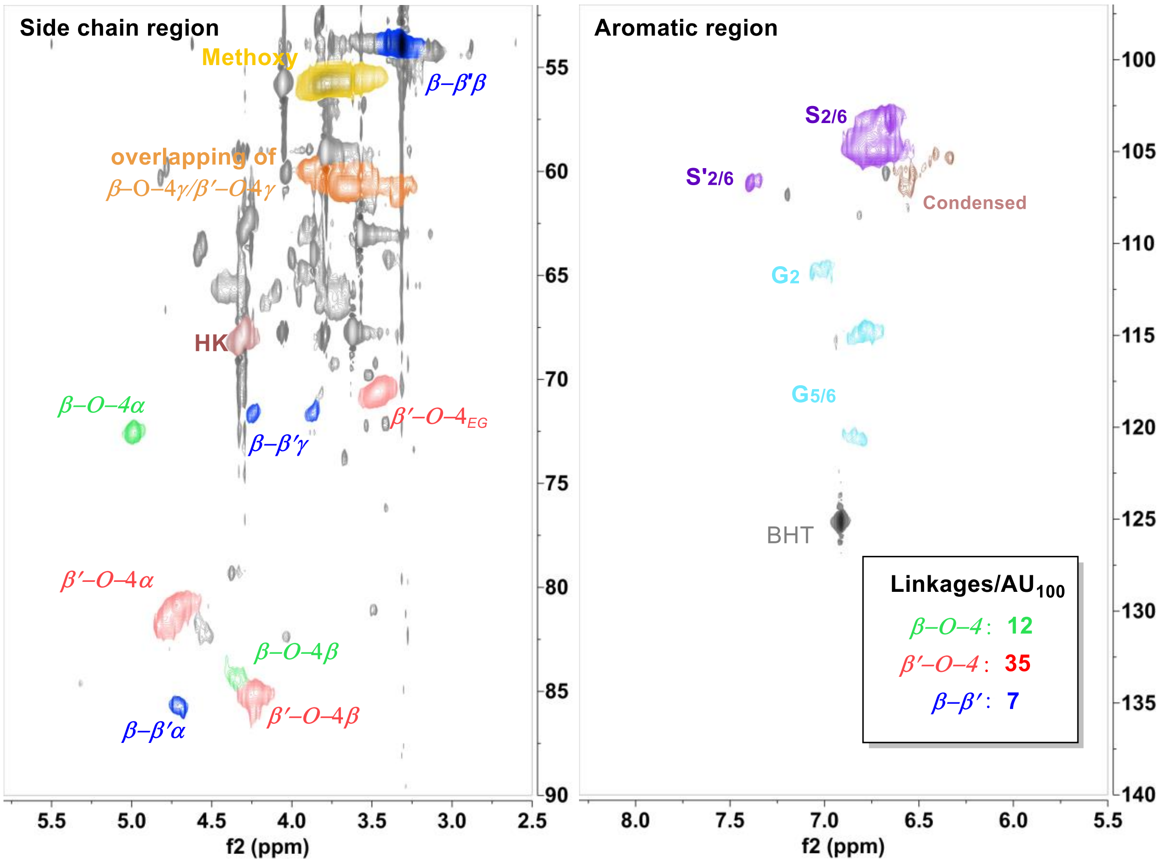

Supplementary Figure 44. ^{1}H-^{13}C HSQC NMR (600 MHz, acetone-d_6) of **CS-DES** (ChCl, 16.8 g, 120 mmol: OA, 120 mmol, 15.2 g: EG, 240 mmol, 14.4 g) fractionated lignin at 80 °C for 24 h, $S_{condensed}$% = 17%.

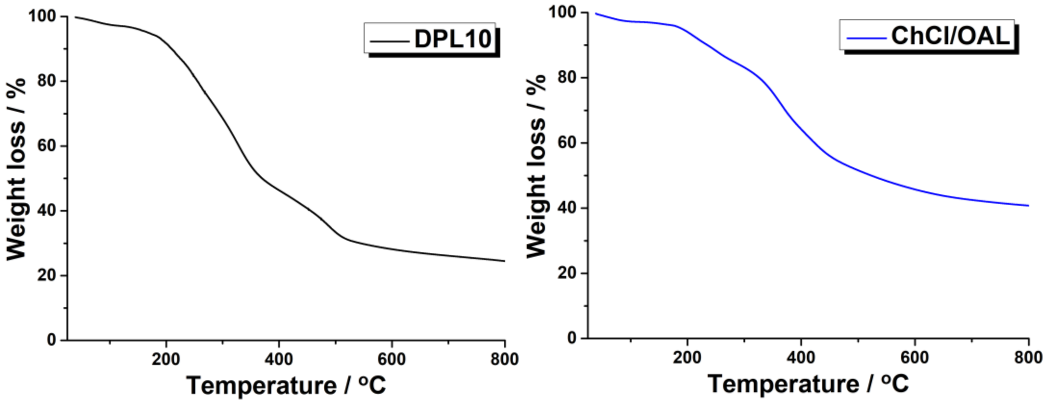

Supplementary Figure 45. The thermal stability of two lignin samples (**DPL10** and **ChCl/OAL**)

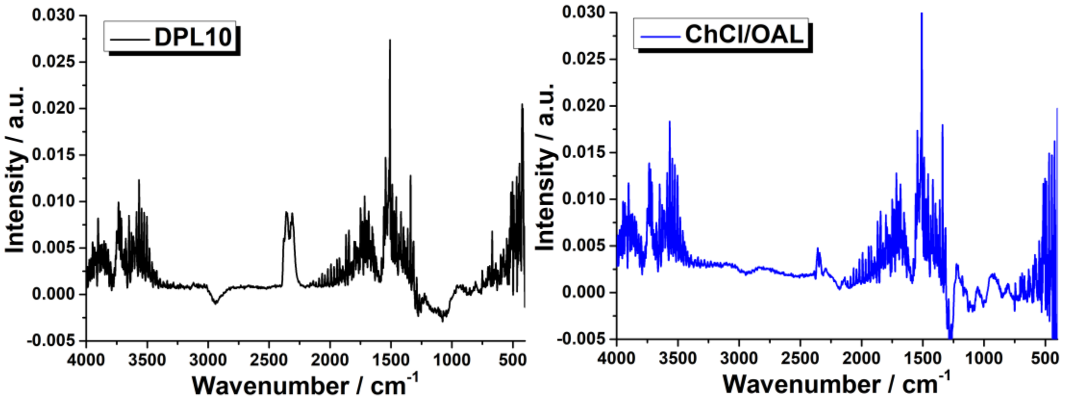

Supplementary Figure 46. FTIR of ChCl/OAL and **DPL10**, note: IR analysis at 220°C (220ºC is also the temperature used for lignin depolymerization by hydrogenolysis).

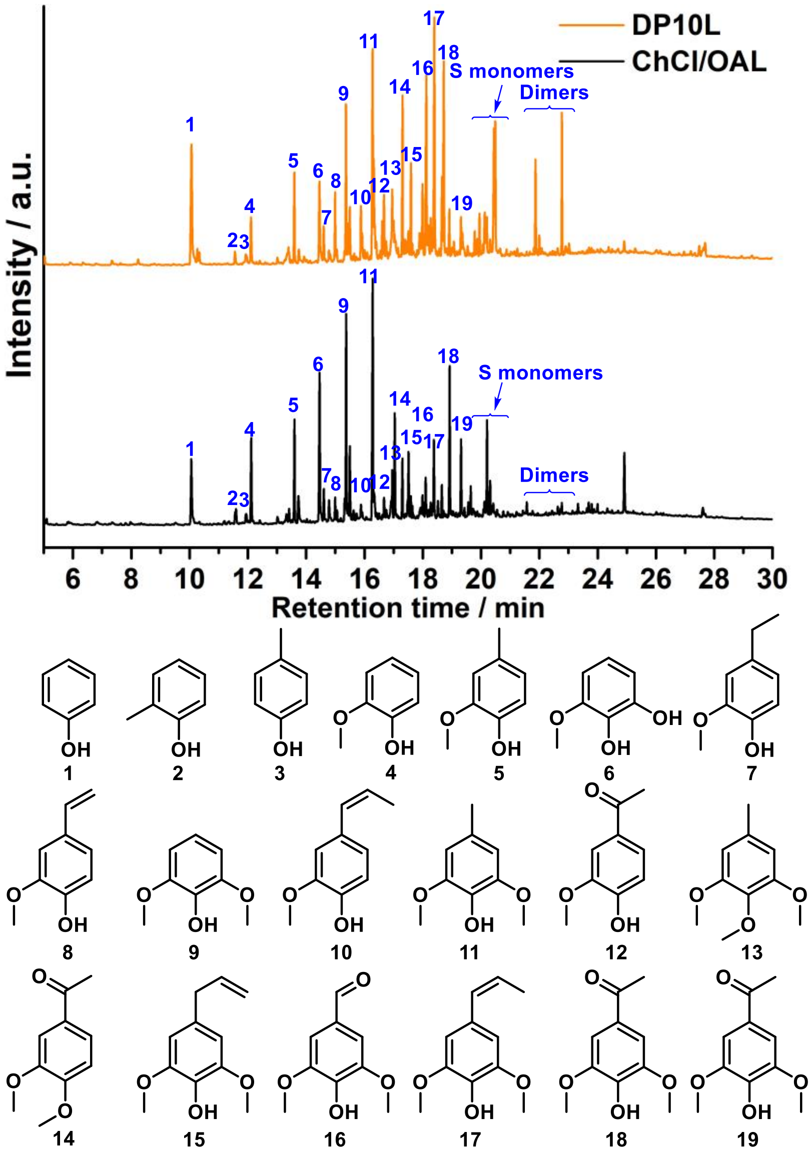

Supplementary Figure 47. Py-GCMS analysis of **ChCl/OAL** and **DPL10** with structures of the main signals.

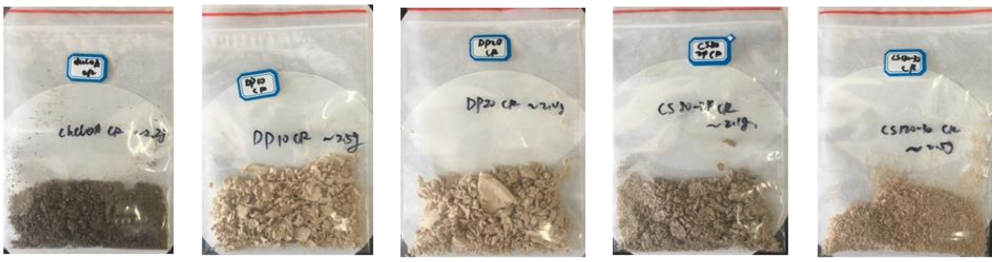

ChCl/OACR DPCR10 DPCR20 CSCR80 CSCR120

Supplementary Figure 48. Digital images of the DES fractionated CRs.

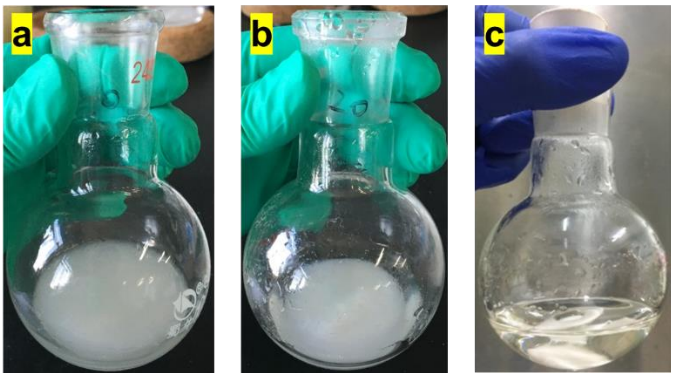

Supplementary Figure 49. Treatment of cellulose and hemicellulose models under different conditions at 100 ℃ for 24h. (a) MCC (0.45 g) in **DP-DES10** (b) MCC (0.45 g) in **DP-DES20** (c) xylan (0.35 g) in **DP-DES10**. The cellulose retention in **DP-DES10** and **DP-DES20** is 99% and 93.5%, respectively.

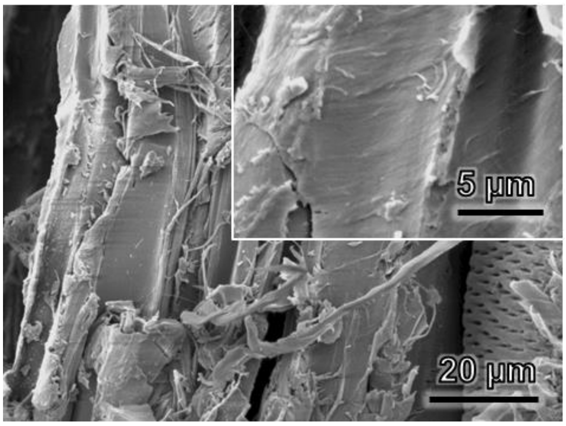

Supplementary Figure 50. SEM of untreated birch lignocellulose, showing a basic wood cell structure at low magnification (left) and smooth surface at high magnification (right).

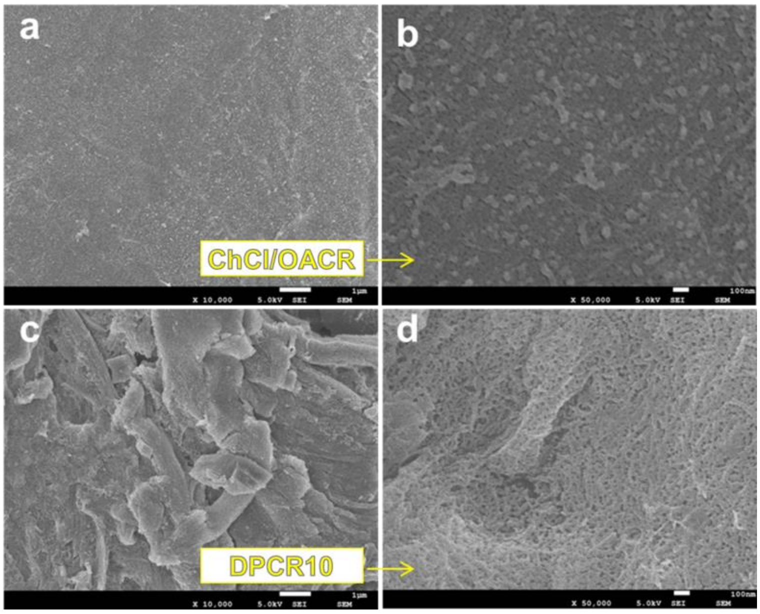

Supplementary Figure 51. SEM images of ChCl/OA DES fractionated CR at low magnification (a) and high magnification (b), showing obvious lignin particles formation on the surface; DP-DES10 fractionated CR at low magnification (c) and high magnification (d), the cell structure of DPCR10 was fibrillated and without formation of lignin particles.

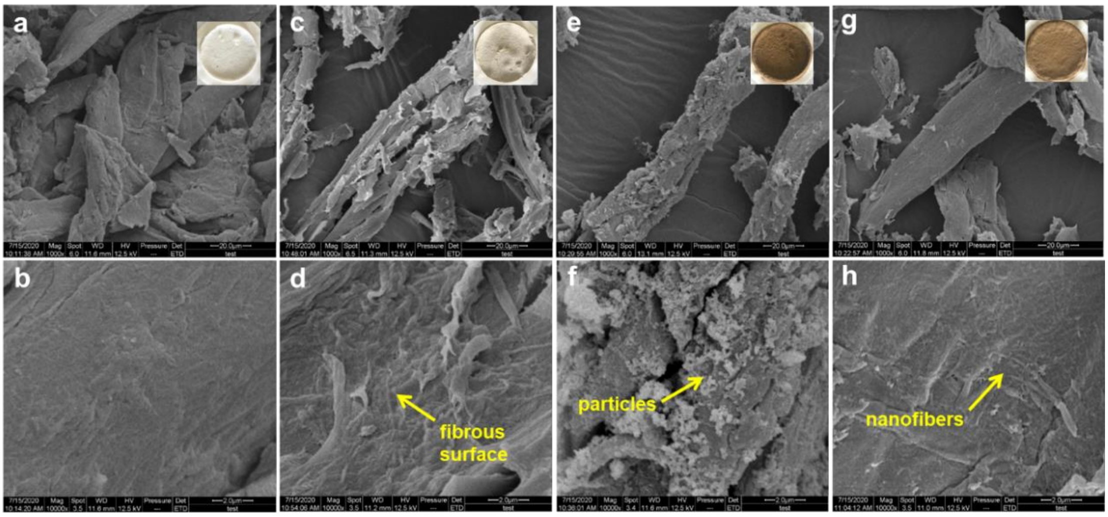

Supplementary Figure 52. SEM images of MCC solid residues obtained with different treatments and additives. **Upper row**: low magnification, **Bottom row**: high magnification. (**a and b):** MCC (0.45 g MCC) treatment in ChCl/OA DES; (**c and d):** MCC and xylan (0.45 g MCC and 0.35 g xylan) treatment in ChCl/OA DES;(**e and f):** MCC+xylan+lignin (0.45 g MCC, 0.35 g xylan and 0.2 g lignin treatment in ChCl/OA DES; (**g and h):**MCC+xylan+lignin (0.45 g MCC, 0.35 g xylan and 0.2 g lignin) treatment in DP-DES10; **Inset:** digital pictures of the solid residues; the recovery of the solid residues and treatment conditions (120 °C, 30min for ChCl/OA DES and 100 °C, 24 h for DP-DES10) follow the same procedure as the lignocellulose fractionation process.

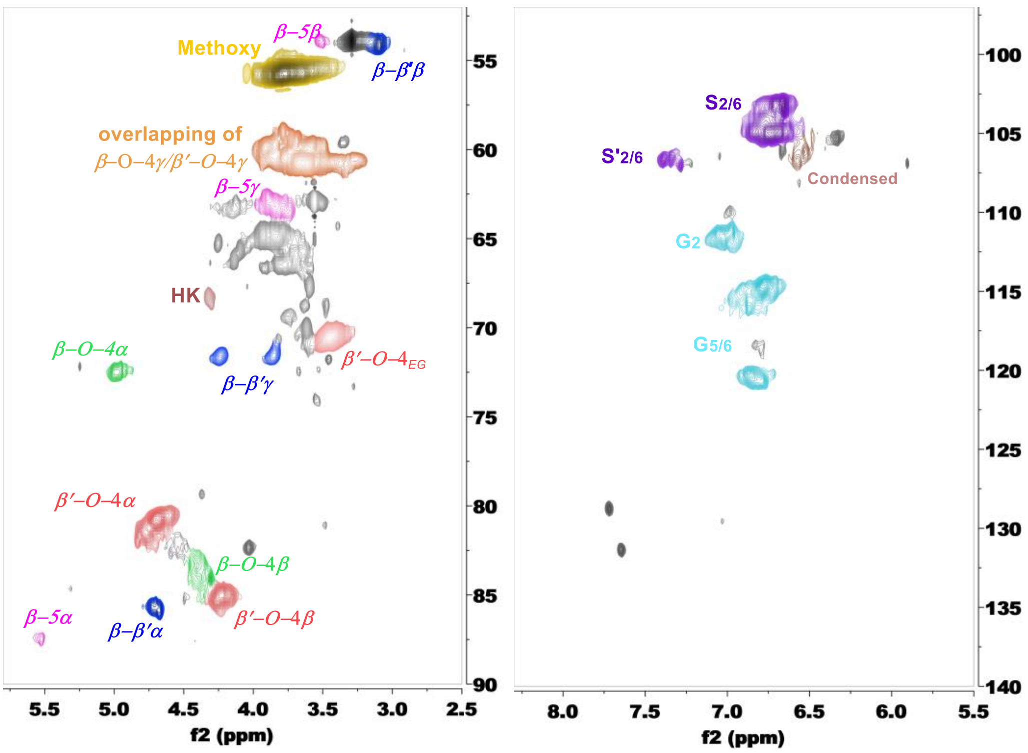

Supplementary Figure 53. 2D HSQC NMR of the lignin (reproducibility experiments) obtained in 44% yield after fractionation with DP-DES10. From the NMR measurement the following parameters are concluded: total β-O-4 content of 52.9 per 100 aromatic units (β'-O-4: 44.7/100Ar and β-O-4: 8.2/100Ar).

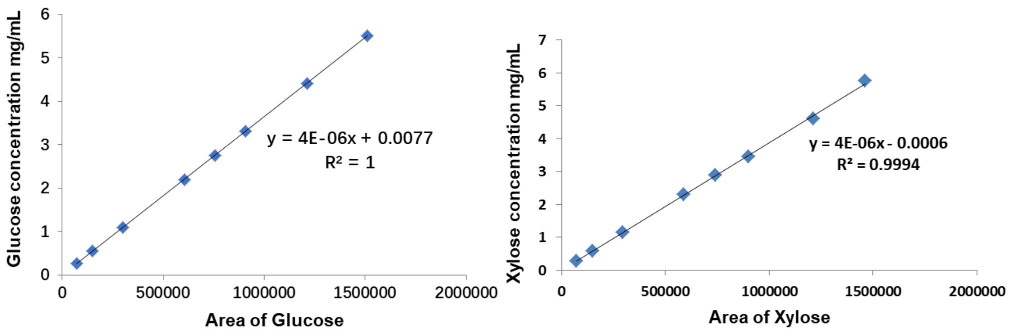

Supplementary Figure 54. Calibration curves of standard glucose and xylose for the yield calculation by HPLC

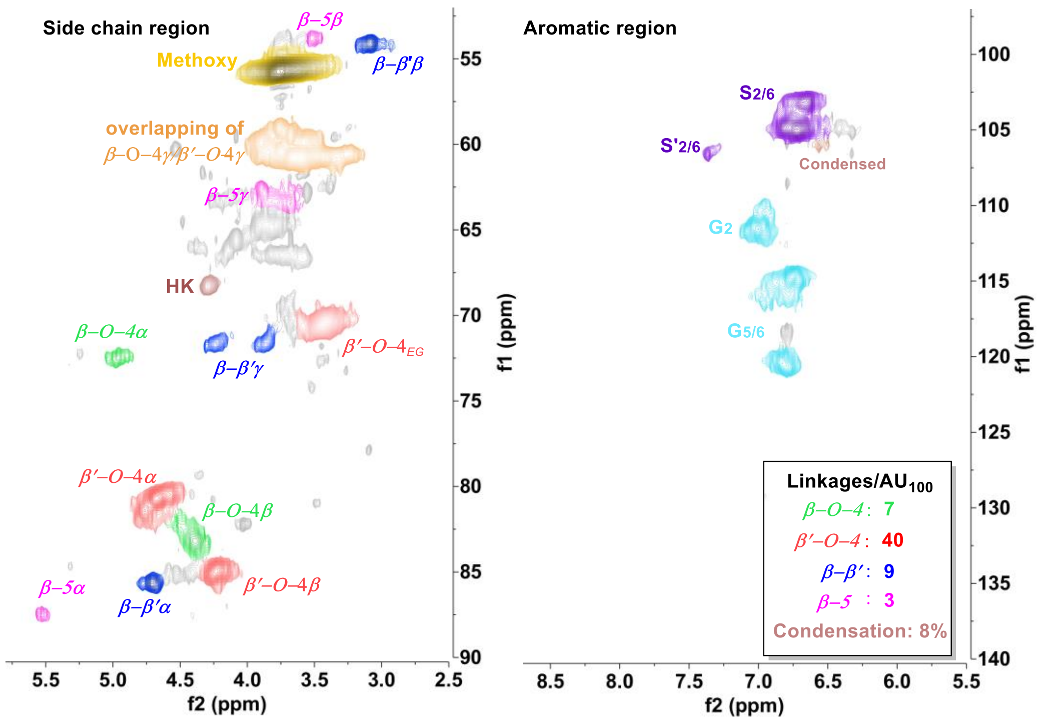

Supplementary Figure 55. 2D HSQC NMR analysis of lignin from EG pulping with OA. Extra lignocellulose fractionation experiment was performed with only EG and OA, following the same reaction conditions as with DP-DES10 (100 °C, 24h). Under these conditions the lignin yield was 18.9%, much lower than what we obtained with the DES systems, clearly showing the benefit of the original DES system. From the NMR measurement the following parameters are concluded: total β-O-4 content of 47 per 100 aromatic units (β'-O-4: 40/100Ar and β-O-4: 7/100Ar).

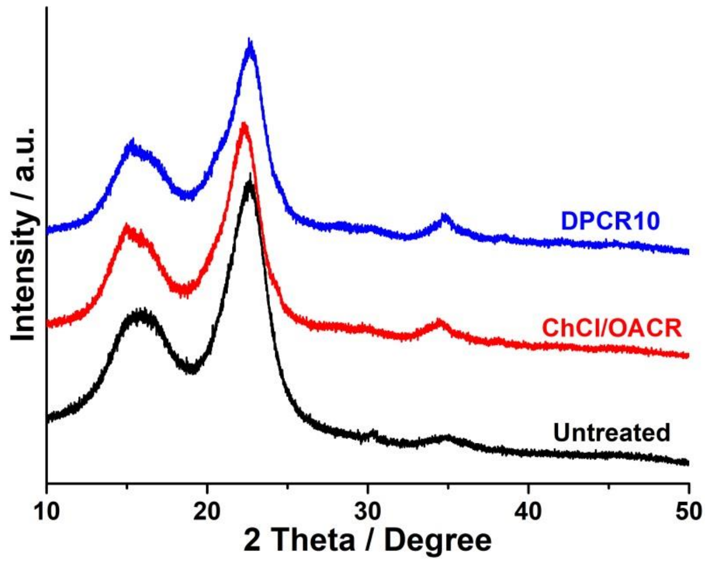

Supplementary Figure 56. XRD curves of untreated wood, ChCl/OACR and DPCR10.

Supplementary Tables

Supplementary Table 1. Price of ternary DES in this work and conventional organic solvent for lignocellulose treatment

Chemicals[a]	Price $/Ton	Purity %
ChCl	400-700	98
OA	780-850	99.6
EG	780-900	99.8
Methanol	400-800	99
Ethanol	900-1190	99
Tetrahydrofuran	1700-2200	99.5
1,4-Dioxane	2850-3500	99.5
**DP-DES**	**600-804**	-
**CS-DES**	**642-811**	-

a: All the price of the chemicals was from Alibaba.com.

Supplementary Table 2. 1a reactions in cleavage/stabilization DES (**CS-DES**) at various temperatures.

Entry	Temperature / °C	Conversion / % **1a**	Selectivity / %				
			**guaiacol**	**6aa**	**5a**	**3a**	**4aa**
1	100	56	26	21	0.3	20	54
2	120	>99	71	67	<1	<1	<1
3	140	>99	59	54	5	0	0

General reaction conditions: 25 mg of **1a**, **CS-DES** prepared with (ChCl 4.2 g, 30 mmol: EG (**2a**) 3.6 g, 60 mmol: OA 3.8 g, 30 mmol), 20 min. Conversion and selectivity were determined by GC-FID using octadecane as an internal standard.

Supplementary Table 3. 1a reactions in cleavage/stabilization DES (**CS-DES**) with various amounts of EG.

Entry	EG amount / mol	Conversion / % **1a**	Selectivity / %				
			**guaiacol**	**6aa**	**5a**	**3a**	**4aa**
1	0.01	>99	63	22	<1	<1	<1
2	0.03	>99	62	51	<1	<1	<1
3	0.06	>99	71	67	<1	<1	<1
4	0.09	>99	61	61	<1	<1	<1

General reaction conditions: 25 mg of **1a**, **CS-DES** prepared with (ChCl 4.2 g, 30 mmol: EG 0.6-5.4 g, 10-90 mmol: OA 3.8 g, 30 mmol), 120 °C, 20 min. Conversion and selectivity were determined by GC-FID using octadecane as an internal standard.

Supplementary Table 4. 1a reactions in cleavage/stabilization DES (**CS-DES**) at various times.

Entry	Time / min	Conversion / % **1a**	Selectivity / %				
			**guaiacol**	**6aa**	**5a**	**3a**	**4aa**
1	5	85	9	6	<1	11	80
2	10	88	26	23	<1	11	63
3	20	>99	71	67	<1	<1	<1
4	30	>99	65	59	<1	<1	<1

General reaction conditions: 25 mg of **1a**, **CS-DES** prepared with (ChCl 4.2 g, 30 mmol: EG 3.6 g, 60 mmol: OA 3.8 g, 30 mmol), 120 °C. Conversion and selectivity were determined by GC-FID using octadecane as an internal standard.

Supplementary Table 5. 1a reactions in derivatization/protection DES (**DP-DES**) at various temperatures.

Entry	Temperature / °C	Conversion / % **1a**	Selectivity / %				
			**guaiacol**	**6aa**	**5a**	**3a**	**4aa**
1	80	94	6	0	3.6	2	95
2	100	>99	21.7	0	20	12	66

General reaction conditions: 50 mg of **1a**, **DP-DES** prepared with (ChCl 4.2 g,30 mmol; EG 3.6 g, 60 mmol; 1 wt% OA 0.08 g, 0.6 mmol), 24 h. Conversion and selectivity were determined by GC-FID using octadecane as an internal standard.

Supplementary Table 6. 1a reactions in derivatization/protection DES (**DP-DES**) with various amounts of OA.

Entry	OA amount / wt%	Conversion / % **1a**	Selectivity / %				
			**guaiacol**	**6aa**	**5a**	**3a**	**4aa**
1	1	77	<1	0	1.6	6	94
2	2	91	4	0	3	9.6	84
3	4	96	<1	0	8	10.3	81
4	7	96	8	0	11	9.4	71
5	10	94	7	0	12	10.7	70

General reaction conditions: 50 mg of **1a**, **DP-DES** prepared with (ChCl 4.2 g, 30 mmol: EG 3.6 g, 60 mmol: 1-10wt% OA), 80 °C, 6 h. Conversion and selectivity were determined by GC-FID using octadecane as an internal standard.

Supplementary Table 7. 1a reactions in derivatization/protection DES (**DP-DES**) at various times.

Entry	Time / h	Conversion / % **1a**	Selectivity / %				
			**guaiacol**	**6aa**	**5a**	**3a**	**4aa**
1	1	25	0	0	0	6	94
2	3	55	0	0	0	6	99
3	6	77	<1	0	1.6	6	94
4	12	85	5	0	2.3	4	93
5	24	94	6	0	3.6	2	95

General reaction conditions: 50 mg of **1a**, DP-DES prepared with (ChCl 4.2 g, 30 mmol: EG 3.6 g, 60 mmol: 1 wt% OA 0.08 g, 0.6 mmol), 80 °C. Conversion and selectivity were determined by GC-FID using octadecane as an internal standard.

Supplementary Table 8. Different characteristics of the prepared DES (both measured and literature values)

DES	Viscosity [cP] (literature values)	D_{ChCl} (literature data)	D_{ChCl} (measured)
ChCl:OA (1:1)	450[9]		$0.7 \cdot 10^{-11}$ m^2/s
ChCl:EG (1:2)	37[35]	$2.62 \cdot 10^{-11}$ m^2/s[35]	$2.6 \cdot 10^{-11}$ m^2/s
ChCl:OA:EG (1:1:2)	68[9]	No data	$0.9 \cdot 10^{-11}$ m^2/s

Supplementary Table 9. Comparison of viscosities, diffusion coefficients of ChCl and radii of the diffusing molecule for the three studied DESs

**DES**	**Viscosity** [cP] (literature values)	**D_{ChCl} measured** [m^2/s]	**r** [m]
ChCl:OA	450[9]	$0.7 \cdot 10^{-11}$ m^2/s	$69.3 \cdot 10^{-12}$
ChCl:EG	37[35]	$2.6 \cdot 10^{-11}$ m^2/s	$226.9 \cdot 10^{-12}$
ChCl:OA:EG	68[9]	$0.9 \cdot 10^{-11}$ m^2/s	$356.6 \cdot 10^{-12}$

Supplementary Table 10. The electron density (ρ) and Laplacian density ($\nabla^2\rho$) for the ChCl/OA and ChCl/EG at the bond critical points (BCPs) calculated at the B3LYP/6-311+G** level.

DES	Hydrogen bonds	P (0.002-0.035)	$\nabla^2\rho$ (0.024-0.139)
ChCl/OA	Cl-...HO	0.0476	0.0656
	Cl-...HC	0.0029	0.0079
	Cl-...HC	0.0046	0.0124
	Cl-...HC	0.0067	0.0184
	OH...HC	0.0103	0.0365
ChCl/EG	CH...HC	0.0029	0.0099
	Cl-...HO	0.0295	0.0699
	Cl-...HC	0.0072	0.0194
	CH...HC	0.0003	0.0013

Supplementary Table 11. Summary of pH values of **DP-DES10** in various stages of recycling and other DES.

Entry	DES	pH[a]	Comment on conditions
**1**	DP10	1.06	Before recycling
**2**	RDP10	2.57	After recycling
**3**	RDP10	1.07	Addition of 107mg **OA** to recycled DES
**4**	RDP10	2.51	The DES was recovered by adding OA, reused and recycled
**5**	RDP10	1.59	Control reaction by mixing the DES and lignocellulose, then directly recycled the DES following the recycling procedure
**6**	DP20	0.9	-
**7**	ChCl/OA	0.62	-
**8**	CS	0.74	-

The pH values were determined by dissolving 1 mL DES in 9mL water by OHAUS ST3100 pH meter according to literature procedures.[55] [a]Average value of 3 parallel experiments reported.

Supplementary Table 12. "Estimated purity" of DES and recycled DES calculated by semi-quantification of ^{1}H NMR.

DES	Calculation	Estimated Purity %
DP10	^{1}H NMR	100[a]
RDP10	^{1}H NMR	99.8[b]
Recycled re-adjusted RDP10[c]	^{1}H NMR	98.4[b]

a: The esters from the DES components were assigned as shown in Supplementary Figures 51-53, it is assumed that the "estimated purity" of freshly prepared DP-DES10 is 100%; **b:** the "estimated purity" of DES according to the ^{1}H NMR is calculated: proton of all the unknown impurities/total protons in recycled DES×100%; **c:** the RDP-DES10 was re-adjusted by adding OA and EG and used for next fractionation, the re-adjusted RDP-DES10 was recycled to determine the "estimated purity".

Supplementary Table 13. Assignment of ^{13}C-^{1}H cross-signals in the HSQC NMR spectra.[38]

**^{13}C-^{1}H cross-signals**	**^{1}H range/^{13}C range**
β-O-4α	(4.76-5.10)/(73-77.5)
β'-O-4α	(4.46-4.77)/(79.9-82.8)
β-O-4β and β'-O-4β	(4.03-4.23)/(84.1-86.4)
β-O-4γ and β'-O-4γ	(3.21-3.97)/(58.6-61.7)
β'-O-4_{EG}	(3.22-3.5)/(69.2-72.3)
β-5α	(5.42-5.63)/(88-92)
β-5β	(3.36-3.56)/(53-54.5)
β-5γ	(3.50-4.00)/(62-64.5)
β-βα	(4.59-4.77)/(86.5-89.5)
β-ββ	(2.98-3.20)/(55.5-59)
β-βγ	(3.75-3.96)/(72.5-76) and (4.10-4.31)/(72.5-76)
HKγ	(4.20-4.30)/(66-68)
$S_{2/6}$	(6.48-6.90)/(104-109)
$S'_{2/6}$	(7.17-7.50)/(105-109)
$S_{condensed}$	(6.35-6.65)/(106-109)
G_2	(6.78-7.14)/(111.5-116)
G_5	(6.48-7.06)/(115-120.5)
G_6	(6.65-6.96)/(120.5-124.5)

Supplementary Table 14. Relative abundance of the compounds identified after pyrolysis of DPL10 and ChCl/OAL

**Compound code[a]**	**Retention time/min**	**DPL10**	**ChCl/OAL**
		**Relative abundance/%**	**Relative abundance/%**
**1**	10.6	0.1	0.2
**2**	11.6	0.2	1.4
**3**	11.9	0.6	0.9
**4**	12.1	2.2	7.1
**5**	13.6	4.0	8.5
**6**	14.5	4.6	11.9
**7**	14.6	2.2	2.9
**8**	15.0	4.0	2.3
**9**	15.4	6.5	17.2
**10**	15.9	3.1	1.5
**11**	16.3	11.9	20.1
**12**	16.7	3.9	2.3
**13**	17.0	4.2	4.5
**14**	17.3	9.3	4.6
**15**	17.6	5.3	2.4
**16**	18.1	10.4	1.0
**17**	18.4	13.6	3.0
**18**	18.7	11.2	1.2
**19**	19.3	2.5	7.0

a: the compounds were identified by comparing the mass spectra with those of the Wiley and NIST libraries.

Supplementary Table 15. Examples of monomers yield from catalytic lignocellulose or lignin hydrogenolysis in the literature.

Lignin substrate	Catalyst, Solvent	Conditions °C, h	Aromatic monomers yield wt%	Reference
**DPL10 (up to 50% yield)**	Ru/C, MeOH (H_2)	220, 18	24 [distinct aromatics]	This work
DES lignin from switchgrass[14]	Pyrolysis (He)	500, -	7-12 [complex mixture]	Ref 22
DES extracted lignin from Sorghum [78% yield][45]	Ru/C, 2-propanol (N_2)	270, 1	9.9 [complex mixture]	Ref 23
Birch wood[46]	Ni/C, MeOH (N_2)	200, 6	20-32	Ref 24
Birch wood[47]	Ru/C, MeOH (H_2)	250, 6	52	Ref 25
Birch wood[48]	Ni/C, MeOH (Ar)	200, 6	48.6	Ref 26
Propionaldehyde protected birch lignin[49]	Ru/C, dioxane (H_2)	250, 15	41.6	Ref 27
Birch organosolv lignin (10% yield)[50]	NiAu, H_2O (H_2)	170, 12	14	Ref 28
Organosolv bagasse lignin[51]	[bSmim][HSO4], water (Air)	250, 0.5	14.5	Ref 29
Organosolv lignin[52]	Ni/MSN-Al, MeOH (H_2)	220, 5	30.8	Ref 30
Sugarcane bagasse[53]	Ni/ZrP, isopropanol (H_2)	260, 4	15.1	Ref 31
M. Giganteus and Pine[54]	Pd/C, dioxane (H_2)	200, 24	9-15	Ref 32

Supplementary Table 16. Composition analysis of CRs after fractionation in **CS-DES** or **DP-DES**.

CRs	CR yield %[a]	Cellulose retention %[b]	Hemicellulose rentention %[c]	Delignification %[d]	Lignin yield %[e]
DPCR10[f]	118[i]	98.9	nd	22	-
DPCR10	70.07 ± 7.47	97.7±1.50	39.28 ± 1.55	56.0±2.14[i]	40.0±3.5
RDPCR10	102[i]	96.2	nd	56	41
DPCR20	57.08 ± 0.76	91.9±0.87	30.95 ± 2.18	67.9±4.62[i]	53.3±10.7
CSCR120[g]	63.29 ± 2.06	94.7±1.42	41.79 ± 0.63	61.4±1.92	44
CSCR80[g]	61.75 ± 1.01	93.4±1.00	39.83 ± 0.64	68.5±3.55	43
ChCl/OACR[h]	52.78 ± 0.14	92±3.43	22.69 ± 2.15	49.6±3.68[i]	51

General condition: 2 g birch lignocellulose, ~32 g **DP-DES**, 100 °C for 24 h, CR and lignin yields were calculated by weight percentage to lignocellulose, lignin was isolated by THF extraction method. **a:** Weight of the recovered residue (cellulose residue, CR) as % of the initial lignocellulose input. **b:** Cellulose retention in the residue based on glucose yield after acid hydrolysis determined by HPLC. **c:** Hemicellulose retention based on analysis by HPAEC-PAD after acid hydrolysis (see more details in Supplementary Table 18). **d:** Removed lignin based on Klason lignin content of the solid residue. **e:**The yield of lignin was calculated to the Klason lignin content in birch after correcting by incorporation correction factor.[39] **f:** Reaction temperature at 80 °C; **g:** ~46 g **CS-DES** was used, 120 °C for 30 min; **h:** binary DES of ChCl/OA was used, 120 °C for 30 min. Cellulose and hemicellulose retention were calculated to the cellulose and hemicellulose content in untreated birch lignocellulose, lignin yield was calculated to Klason lignin content in untreated birch lignocellulose. **i:** CR yield exceeds 100% likely due to EG incorporation as also seen in Ref 41 in the main text.

Supplementary Table 17. Composition analysis of the DES fractionated CRs both in the lab of China and the Netherlands.

Data –Northeast Forestry University–birch wood (*Betula costata,* China*)*

CRs	CR yield %	Cellulose retention %	Hemicellulose retention %	Delignification %
**ChCl/OA CR**	53.5±1.43	92±3.43	14.3±0.66	49.6±3.68
**DPCR10**	67.8±0.80	97.7±1.50	43.9±1.63	48.8±1.83
**DPCR20**	59.5±0.44	91.9±0.87	27.0±4.25	74.0±1.62
**CSCR120**	59.7±2.60	94.7±1.42	27.5±1.83	61.4±1.92
**CSCR80**	52.7±1.04	93.4±1.00	17.0±0.47	68.5±3.55

Data – University of Groningen – from birch wood (Netherlands, Europe)

CRs	CR yield %	Cellulose retention %	Hemicellulose retention %	Delignification %
**ChCl/OA CR**	52.8±0.14	84.3±0.64	17.6±0.76	74.3±0.46
**DPCR10**	65.0±1.16	98.0±1.45	39.3±2.12	55.2±1.86
**DPCR20**	57.0±0.64	88.2±1.39	24.4±0.98	68.7±1.11
**CSCR120**	63.3±2.06	87.7±0.24	29.7±0.42	56.6±4.08
**CSCR80**	61.8±1.01	87.7±1.66	29.0±0.48	57.1±2.44

Supplementary Table 18. Hemicellulose retention and composition [% w/w] after various DES treatments.

	Birchwood raw	DPCR10	DPCR20	CSCR80	CSCR120	ChCl/OACR
**Yield & retention [%w/w] [a]**						
Yield residue [b]	100.00	70.07 ± 7.47	57.08 ± 0.76	61.75 ± 1.01	63.29 ± 2.06	52.78 ± 0.14
Yield hemicellulose in residue [c]	28.32 ± 0.28	16.11 ± 2.22	15.37 ± 1.70	18.26 ± 0.57	18.71 ± 0.35	12.18 ± 1.21
% Hemicellulose retention (residue) [d]	100.00	39.28 ± 1.55	30.95 ± 2.18	39.83 ± 0.64	41.79 ± 0.63	22.69 ± 2.15
**Xylan composition [%w/w]**						
Xylose	25.33 ± 0.31	15.69 ± 2.23	15.44 ± 2.02	17.92 ± 0.62	18.27 ± 0.41	12.14 ± 1.17
**Xylan substituents:**						
Galactose	1.72 ± 0.01	0.15 ± 0.01	0.00 ± 0.00	0.09 ± 0.06	0.11 ± 0.07	0.00 ± 0.00
Rhamnose	0.60 ± 0.00	0.22 ± 0.04	0.10 ± 0.02	0.22 ± 0.07	0.27 ± 0.08	0.04 ± 0.00
Arabinose	0.50 ± 0.01	0.00 ± 0.00	0.00 ± 0.00	0.00 ± 0.00	0.00 ± 0.00	0.00 ± 0.00
Glucuronic Acid	0.17 ± 0.01	0.05 ± 0.00	0.03 ± 0.00	0.04 ± 0.01	0.06 ± 0.01	0.00 ± 0.00
Mannose	0.00 ± 0.00	0.00 ± 0.00	0.00 ± 0.00	0.00 ± 0.00	0.00 ± 0.00	0.00 ± 0.00
**Degree of substitution:**						
Rha/Xyl[e]	2.4	1.4 ± 0.28	0.6 ± 0.02	1.2 ± 0.39	1.5 ± 0.04	0.3 ± 0.27
Ara/Xyl[e]	2.0	0.0 ± 0.00	0.0 ± 0.00	0.0 ± 0.00	0.0 ± 0.00	0.0 ± 0.00
Gal/Xyl[e]	6.8	0.9 ± 0.13	0.5 ± 0.37	0.5 ± 0.25	0.9 ±0.29	0.0 ± 0.00
GlcA/Xyl[e]	0.7	0.3 ± 0.05	0.3 ± 0.06	0.2 ± 0.04	0.3 ± 0.04	0.0 ± 0.00
Total substituents/Xyl[e]	11.8	2.7 ± 0.42	1.4 ± 0.53	1.9 ± 0.66	2.7 ±0.35	0.3 ± 0.27

Values ≤ 1 are considered traces. Data represents values from an average of 4-6 analysis runs.

[a] Weight percentage is based on dry matter of birchwood raw used for all DES treatments.

[b] Weight percentage of residue after DES treatments.

[c] Weight percentage of hemicellulose content in residue after DES treatments.

[d] Weight percentage of hemicellulose retention based on untreated birchwood raw.

[e] Ratio mol/100 mol; Rha- rhamnosyl. Ara – arabinosyl. Gal – galactosyl. GlcA – glucuronyl. Xyl- xylosyl. Man - mannose residues. Total substituents = Gal+ Rha + Ara + GlcA + Man

Supplementary Table 19. Glucose yield from enzymatic hydrolysis of CR.

Time / h	Glucose yield %					
	Untreated	ChCl/OACR	DPCR10	CSCR120	DPCR20	CSCR80
6	13.8±1.00	17.7±1.14	49.9±2.80	23.3±0.83	43.3±1.68	35.3±1.01
12	15.5±1.03	30.5±1.71	67.5±3.49	38.2±2.04	58.6±2.58	51.5±1.31
24	18.0±1.34	41.9±3.28	79.8±1.40	53.4±1.81	72.1±2.16	69.7±1.66
48	21.0±1.93	56.7±4.58	88.1±1.20	73.5±2.35	82.5±2.45	79.0±1.38
72	21.6±2.01	59.1±5.46	96.5±3.54	77.0±3.35	91.6±0.89	84.4±2.04
96	23.1±2.05	58.8±5.01	95.9±2.12	80.1±1.77	92.5±1.63	87.8±2.77

Glucose yield was calculated to the cellulose content in corresponding CRs and determined by HPLC. The yields were average values from a set of at least 3 experiments showing standard deviations.

Supplementary Table 20. Xylose yield from enzymatic hydrolysis of CR.

Time / h	Xylose yield %					
	Untreated	ChCl/OACR	DPCR10	CSCR120	DPCR20	CSCR80
6	5.2±0.17	28.2±1.07	45.0±3.71	41.1±1.76	55.1±2.10	49.9±3.42
12	6.1±0.47	39.8±3.53	57.0±2.57	47.0±1.90	62.5±2.02	58.6±1.69
24	7.1±0.53	57.5±4.30	71.2±1.62	61.9±2.20	76.5±3.23	70.4±5.09
48	8.8±0.69	78.8±1.60	80.9±2.85	75.4±1.23	83.9±1.55	83.7±2.02

72	7.9±0.68	77.9±2.61	82.6±4.62	78.5±0.38	84.5±2.21	86.0±1.58
96	9.6±0.68	75.3±1.40	86.7±1.53	78.6±1.89	83.9±1.44	84.9±0.94

Xylose yield was calculated to the hemicellulose content in corresponding CRs and determined by HPLC. The yields were average values from a set of at least 3 experiments showing standard deviations.

Supplementary Table 21 Surface area and pore volumes determinted by BET

Samples	BET surface area / m^2/g	BJH volume of pores / cm^2/g
Untreated wood	0.62	nd[a]
ChCl/OACR	1.68	0.0029
DPCR10	1.89	0.0036

a: Not detectable

Supplementary References

1. Bussi, G., Donadio, D. & Parrinello, M. Canonical Sampling Through Velocity Rescaling. *The Journal of Chemical Physics* **126**, 14101 (2007).
2. Martonák R. *et al.* Predicting Crystal Structures: The Parrinello-Rahman Method Revisited. *Physical Review Letters* **90**, 75503 (2003).
3. Hess, B., Bekker, H., Berendsen, H. & Fraaije, J. LINCS: A linear constraint solver for molecular simulations. *Journal of Chemical Theory & Computation* **4**, 1463–1472 (2008).
4. Ma, Y., Xia, Q., Liu, Y., Chen, W. & Yu, H. Production of Nanocellulose Using Hydrated Deep Eutectic Solvent Combined with Ultrasonic Treatment. *ACS Omega* **4**, 8539–8547 (2019).
5. Xia, Q. *et al.* Multiple hydrogen bond coordination in three-constituent deep eutectic solvents enhances lignin fractionation from biomass. *Green Chemistry* **20**, 2711–2721 (2018).
6. Sluiter, A. *et al.* Determination of structural carbohydrates and lignin in Biomass. *Laboratory analytical procedure* 1–16 (2018).
7. Gosselink, R. J. A. *et al.* Lignin depolymerisation in supercritical carbon dioxide/acetone/water fluid for the production of aromatic chemicals. *Bioresource Technology* **106**, 173–177 (2012).
8. Wen, J. L., Sun, S. L., Xue, B. L. & Sun, R. C. Quantitative structures and thermal properties of birch lignins after ionic liquid pretreatment. *Journal of Agricultural and Food Chemistry* **61**, 635–645 (2013).
9. Tang, W., Li, G., Chen, B., Zhu, T. & Row, K. H. Evaluating ternary deep eutectic solvents as novel media for extraction of flavonoids from Ginkgo biloba. *Separation Science and Technology* **52**, 91–99 (2017).
10. Jiang, Z. M. *et al.* Green and efficient extraction of different types of bioactive alkaloids using deep eutectic solvents. *Microchemical Journal* **145**, 345–353 (2019).
11. Chen, Z., Jacoby, W. A. & Wan, C. Ternary deep eutectic solvents for effective biomass deconstruction at high solids and low enzyme loadings. *Bioresource*

Technology **279**, 281–286 (2019).

12. Ji, Q. et. al. Efficient removal of lignin from vegetable wastes by ultrasonic and microwave-assisted treatment with ternary deep eutectic solvent *Industrial Crops and Products* **149**, 112357 (2020).
13. Xue, B., Yang, Y., Tang, R., Xue, D. & Li, X. Efficient dissolution of lignin in novel ternary deep eutectic solvents and its application in polyurethane. *International Journal of Biological Macromolecules* **164**, 480–488 (2020).
14. Chen, Z., Bai, X., Lusi, A., Zhang, H. & Wan, C. Insights into Structural Changes of Lignin Toward Tailored Properties during Deep Eutectic Solvent Pretreatment. *ACS Sustainable Chemistry & Engineering* **8**, 9783–9793 (2020).
15. Jiang, J., Carrillo-Enríquez, N. C., Oguzlu, H., Han, X. & Jiang, F. High Production Yield and More Thermally Stable Lignin-Containing Cellulose Nanocrystals Isolated Using a Ternary Acidic Deep Eutectic Solvent. *ACS Sustainable Chemistry & Engineering* **8**, 7182–7191 (2020).
16. Saputra, R., Walvekar, R. & Khalid, M. Synthesis and thermophysical properties of ethylammonium chloride-glycerol-ZnCl2 ternary deep eutectic solvent - ScienceDirect. *Journal of Molecular Liquids* **310**, 113232 (2020).
17. Farajzadeh, M. A., Abbaspour, M., Kazemian, R. & Mogaddam, M. Preparation of a new three-component deep eutectic solvent and its use as an extraction solvent in dispersive liquid–liquid microextraction of pesticides in green tea and herbal distillates. *Journal of the Science of Food and Agriculture* **100**, 1904–1912 (2020).
18. Fu, N., Liu, X., Li, L., Tang, B. & Row, K. H. Ternary choline chloride/caffeic acid/ethylene glycol deep eutectic solvent as both a monomer and template in a molecularly imprinted polymer. *Journal of Separation Science* **40**, 2286–2291 (2017).
19. Supek, E., Mako, P. & Gbicki, J. Theoretical and Economic Evaluation of Low-Cost Deep Eutectic Solvents for Effective Biogas Upgrading to Bio-Methane. *Energies* **13**, 3379 (2020).
20. Jian, S. *et al.* One-pot integrated biofuel production using low-cost

biocompatible protic ionic liquids. *Green Chemistry* **19**, 3152–3163 (2017).

21. Zang, G., Shah, A. & Wan, C. Techno-economic analysis of coproduction of 2,3-butanediol, furfural, and technical lignin via biomass processing based on deep eutectic solvent pretreatment. *Biofuels Bioproducts and Biorefining* **14**, 326–343 (2020).
22. Wang, S., Yin, K., Zhang, Y. & Liu, H. Glycerol Hydrogenolysis to Propylene Glycol and Ethylene Glycol on Zirconia Supported Noble Metal Catalysts. *ACS Catalysis* **3**, 2112–2121 (2013).
23. Dapsens, P. Y., Mondelli, C. & Pérez-Ramírez, J. Biobased chemicals from conception toward industrial reality: Lessons learned and to be learned. *ACS Catalysis* **2**, 1487–1499 (2012).
24. Zhao, G., Zheng, M., Zhang, J., Wang, A. & Zhang, T. Catalytic conversion of concentrated glucose to ethylene glycol with semicontinuous reaction system. *Industrial and Engineering Chemistry Research* **52**, 9566–9572 (2013).
25. Wang, A. & Zhang, T. One-Pot Conversion of Cellulose to Ethylene Glycol with Multifunctional Tungsten-Based Catalysts. *Accounts of Chemical Research* **46**, 1377–1386 (2013).
26. Kozlovsky, R. A., Shvets, V. F. & Makarov, M. G. A Kinetic Model of the Choline Chloride Synthesis. *Org.Proc.Res.Dev* **3**, 357–362 (1999).
27. Lu, C., Zhang, L., Yang, G. & Chen, Z. Task Specific Onium Salt as Soluble Support in Multicomponent Synthesis of 4-Aryl-2-amino-3-ethoxycarbonyl-naphthopyrans. *Chinese Journal of Chemistry* **28**, 2469–2473 (2011).
28. Chen, R. R. & Yuan, C. G. Green synthesis of choline chloride. *Modern Chemical Industry* **32**, 59–61 (2012).
29. Wang, A. & Zhang, T. One-Pot Conversion of Cellulose Tungsten-Based Catalysts. **46**, 1377–1386 (2013).
30. Dawange, M., Galkin, M. V. & Samec, J. S. M. Selective aerobic benzylic alcohol oxidation of lignin model compounds: Route to aryl ketones. *ChemCatChem* **7**, 401–404 (2015).

31. Buendia, J., Mottweiler, J. & Bolm, C. Preparation of diastereomerically pure dilignol model compounds. *Chemistry - A European Journal* **17**, 13877–13882 (2011).
32. Deuss, P. J. *et al.* Aromatic Monomers by in Situ Conversion of Reactive Intermediates in the Acid-Catalyzed Depolymerization of Lignin. *Journal of the American Chemical Society* **137**, 7456–7467 (2015).
33. Rodriguez, N. R., Bruinhorst, A., Kollau, L., Kroon, M. C. & Binnemans, K. Degradation of Deep-Eutectic Solvents Based on Choline Chloride and Carboxylic Acids. *ACS Sustainable Chemistry & Engineering* **7**, 11521-11528 (2019).
34. Florindo, C., Oliveira, F. S., Rebelo, L., Fernandes, A. M. & Marrucho, I. M. Insights into the Synthesis and Properties of Deep Eutectic Solvents Based on Cholinium Chloride and Carboxylic Acids. *ACS Sustainable Chemistry* **2**, 2416–2425 (2014).
35. D'Agostino, C., Harris, R., Abbott, A., Gladden, L. & Mantle, M. Molecular motion and ion diffusion in choline chloride based deep eutectic solvents studied by 1H pulsed field gradient NMR spectroscopy. *Physical Chemistry Chemical Physics* **13**, 21383–21391 (2011).
36. Thorat, G. M., Jadhav, H. S., Roy, A., Chung, W. J. & Seo, J. G. Dual role of deep eutectic solvent as a solvent and template for the synthesis of octahedral cobalt vanadate for oxygen evolution reaction. *ACS Sustainable Chemistry & Engineering* **6**, 16255-16266 (2018).
37. Du, C., Zhao, B., Chen, X. B., Birbilis, N. & Yang, H. Effect of water presence on choline chloride-2urea ionic liquid and coating platings from the hydrated ionic liquid. *Scientific Reports* **6**, 29225 (2016).
38. Zijlstra, D. S. *et al.* Extraction of lignin with high β-O-4 content by mild ethanol extraction and its effect on the depolymerization yield. *Journal of Visualized Experiments* **2019**, 1–12 (2019).
39. Zijlstra, D. S., Analbers, C. A., de Korte, J., Wilbers, E. & Deuss, P. J. Efficient mild organosolv lignin extraction in a flow-through setup yielding

lignin with high β-O-4 content. *Polymers* **11**, 14–17 (2019).

40. Cen, Q. L. S. W. Y. Z. L. Mechanism study of wood lignin pyrolysis by using TG–FTIR analysis. *Journal of Analytical and Applied Pyrolysis* **82**, 170–177 (2008).
41. Gírio, F. M. *et al.* Hemicelluloses for fuel ethanol: A review. *Bioresource Technology* **101**, 4775–4800 (2010).
42. Naidu, D. S., Hlangothi, S. P. & John, M. J. Bio-based products from xylan: A review. *Carbohydrate Polymers* **179**, 28–41 (2018).
43. Deuss, P. J. *et al.* Metal Triflates for the Production of Aromatics from Lignin. *ChemSusChem* **9**, 2974–2981 (2016).
44. Deuss, P. J. *et al.* Phenolic acetals from lignins of varying compositions: Via iron(iii) triflate catalysed depolymerisation. *Green Chemistry* **19**, 2774–2782 (2017).
45. Lalitendu, D. *et al.* Characterization and Catalytic Transfer Hydrogenolysis of Deep Eutectic Solvent Extracted Sorghum Lignin to Phenolic Compounds. *ACS Sustainable Chemistry & Engineering* **6**, 10408-10420 (2018).
46. Klein, I., Saha, B. & Abu-Omar, M. M. Lignin depolymerization over Ni/C catalyst in methanol, a continuation: Effect of substrate and catalyst loading. *Catalysis Science and Technology* **5**, 3242–3245 (2015).
47. Van, D. *et al.* Reductive lignocellulose fractionation into soluble lignin-derived phenolic monomers and dimers and processable carbohydrate pulps. *Energy & Environmental Science* **8**, 1748-1763 (2015).
48. Qi, S. *et al.* Lignin depolymerization (LDP) in alcohol over nickel-based catalysts via a fragmentation–hydrogenolysis process. *Energy & Environmental Science* **6**, 994–1007 (2013).
49. Wu *et al.* Protection Group Effects During α,γ-Diol Lignin Stabilization Promote High-Selectivity Monomer Production. *Angew. Chem. Int. Ed.* **130**, 1370–1374 (2018).
50. Zhang, J. *et al.* Highly efficient, NiAu-catalyzed hydrogenolysis of lignin into phenolic chemicals. *Green Chemistry* **16**, 2432–2437 (2014).

51. Li, Y., Cai, Z., Liao, M., Long, J. & Li, X. Catalytic depolymerization of organosolv sugarcane bagasse lignin in cooperative ionic liquid pairs. *Catalysis Today* **298**, 168–174 (2017).
52. Si, X., Chen, J., Lu, F., Liu, X. & Xu, J. Immobilized Ni Clusters in Mesoporous Aluminum Silica Nanospheres for Catalytic Hydrogenolysis of Lignin. *ACS Sustainable Chemistry & Engineering* **7**, 19034–19041 (2019).
53. Ma, H. *et al.* Selective depolymerization of lignin catalyzed by nickel supported on zirconium phosphate. *Green Chemistry* **21**, 658–668 (2019).
54. Gao, F., Webb, J. D., Sorek, H., Wemmer, D. E. & Hartwig, J. F. Fragmentation of Lignin Samples with Commercial Pd/C under Ambient Pressure of Hydrogen. *ACS Catalysis* **6**, 7385–7392 (2016).
55. Shen, X. J. *et al.* Facile fractionation of lignocelluloses by biomass-derived deep eutectic solvent (DES) pretreatment for cellulose enzymatic hydrolysis and lignin valorization. *Green Chemistry* **21**, 275–283 (2019).
